# Pentafluorophenyl Copper–Biarylsulfoxide Complexes: Synthesis and Photoreactivity

**DOI:** 10.3390/molecules29143332

**Published:** 2024-07-16

**Authors:** Valentin Magné, Romaric Lenk, Sonia Mallet-Ladeira, Eddy Maerten, David Madec

**Affiliations:** 1 Laboratoire Hétérochimie Fondamentale et Appliquée (UMR 5069), Université de Toulouse, CNRS, 118 Route de Narbonne, CEDEX 09, 31062 Toulouse, France; romaric.lenk@univ-tlse3.fr (R.L.); eddy.maerten@univ-tlse3.fr (E.M.); 2 Institut de Chimie de Toulouse (UAR 2599), 118 Route de Narbonne, CEDEX 09, 31062 Toulouse, France; sonia.ladeira@univ-tlse3.fr

**Keywords:** biarylsulfoxide complexes, copper(I) complexes, photoreactivity

## Abstract

Pentafluorophenyl copper(I)–biarylsulfoxide complexes, existing as [Cu(C_6_F_5_)]_4_L_2_, both in solution and in the solid state, were prepared and thoroughly characterized. Subsequently, the photochemistry of the complexes was explored, showing inherent photoreactivity of the biarylsulfoxide moiety within the coordination sphere of the copper. Photoinduced cross-coupling reactions between the anthryl moiety of bis-anthracenylsulfoxide and pentafluorobenzene, and synthesis of Cu_2_O (cuprite), were demonstrated.

## 1. Introduction

The sulfoxide function is ubiquitous nowadays in chemistry, as illustrated per the well-known dimethylsulfoxide, which is widely used in medicine as a bio-compatible solvent for biologically relevant molecules [1,2,3,4], or in synthetic chemistry as a solvent presenting advantageous properties such as high polarity, high boiling point, and low toxicity.

**Scheme 1:**
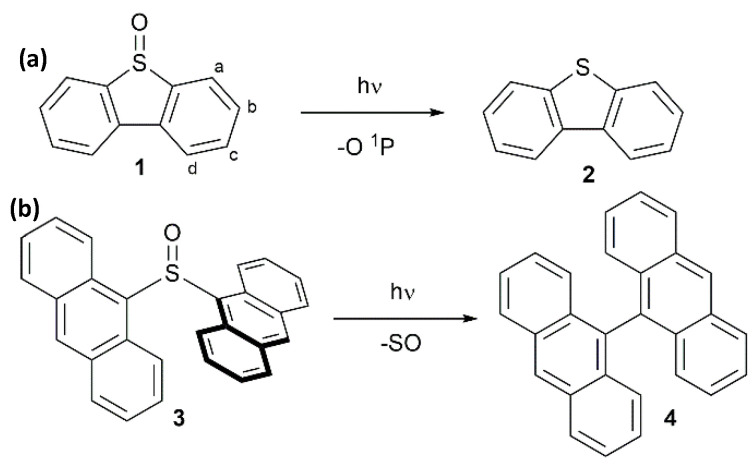
Known photoreactive biarylsulfoxide derivatives. (**a**) Deoxygenation of DBTO. (**b**) SO extrusion of Anthra_2_SO.

Interestingly, some peculiar biarylsulfoxides scaffolds exhibit photoreactivity. For example, it has been known since 1973 that dibenzothiophene-*S*-oxide (DBTO **1**) and its derivatives undergo S–O bond cleavage under UV-light irradiation, releasing the parent photostable dibenzothiophene (DBT **2**) and atomic oxygen (see Scheme 1a) [5,6,7]. Remarkably, some biarylsulfoxides, such as bis-anthracenylsulfoxide (Anthra_2_SO **3**), can undergo a fundamentally different type of photoreactivity, exhibiting extrusion of SO forming bianthryl **4** quantitatively (see Scheme 1b) under UV light [8].

On the other hand, biarylsulfoxides are often disregarded as ligands. Indeed, their weak coordination behavior often implies using a multidentate ligand to ensure that the sulfoxide enters the coordination sphere of the metal. However, those ligands have proved to coordinate via η^1^*-S* or η^1^*-O* bonding with numerous metals [9,10], including palladium, ruthenium, and rhodium, and have found applications in different catalytic processes, mostly exploiting their stereogenicity for the synthesis of chiral compounds [11,12].

Organocopper(I) compounds are known to form aggregates in solution, from species containing two to eight members up to a polymeric structure [13,14]. Interestingly, the addition of bulky and/or chelating ligands allowed breaking the clusters, leading to smaller, well-defined aggregates [15,16], ultimately providing monomeric species [17,18]. In the present study, pentafluorophenyl copper Cu(C_6_F_5_) **5** was chosen as an interesting candidate for the synthesis of copper(I)–biarylsulfoxide complexes because of its versatility. Indeed, this species, existing as a tetramer both in solution and in the solid state [19], has been reported to present different coordination behaviors depending on the nature and stoichiometry of the ligand used [20]. 

Thus, starting from the versatility of pentafluorophenyl copper Cu(C_6_F_5_), from the properties of sulfoxides in coordination chemistry, and from the photoreactivity of bis–arylsulfoxides, we describe in this study the combination of these complementary parameters through the synthesis of new bis-arylsulfoxide–copper(I) complexes and their inherent reactivity under irradiation.

## 2. Results and Discussion

### 2.1. Synthesis and Photoreactivity of [Cu(C_6_F_5_)]_4_(DBTO)_2_

**Figure 1:**
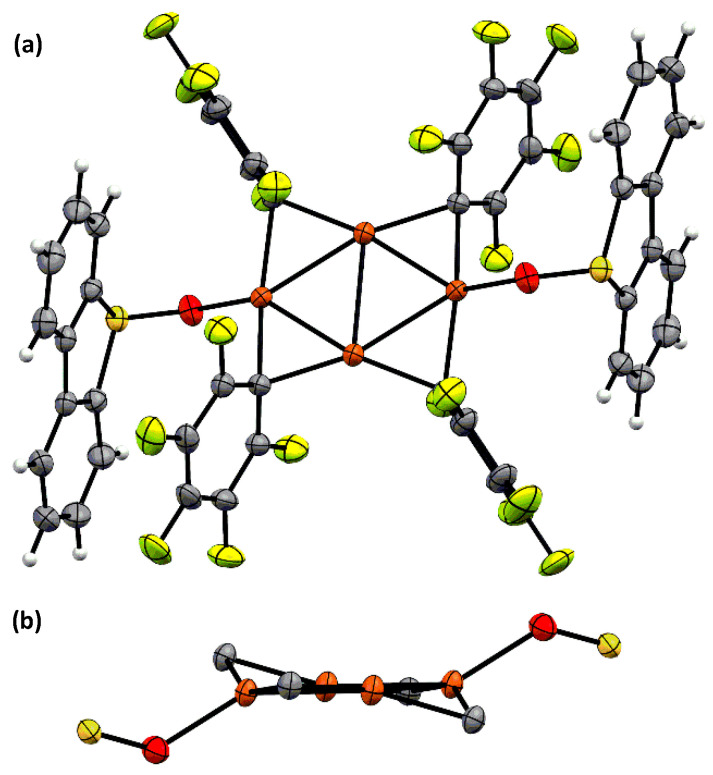
(**a**) ORTEP view of X-ray structure of [Cu(C_6_F_5_)]_4_(DBTO)_2_ **6** at 50% probability. (**b**) View of the Cu_4_ ring coordination structure.

The highly electron-deficient metal core of **5** coordinates to arenes such as toluene, yielding the tetrameric copper cluster [Cu(C_6_F_5_)]_4_(toluene)_2_ [21]. Moreover, in 2012, Jäkle et al. demonstrated the versatility of the coordination of **5** with pyridine, obtaining at will the [Cu(C_6_F_5_)]_4_(pyridine)_2_ tetramer and the Cu(C_6_F_5_)(pyridine) monomer [17]. Interestingly, this study is supported by a systematic NMR investigation of the chemical shifts of both the fluorine and the protons of the organocopper complexes, which provide a strong basis for the present work. The present studies were thus started by mixing equimolar quantities of DBTO **1** and Cu(C_6_F_5_) **5**. Pleasingly, only one set of ^19^F and ^1^H NMR signals was observed, with both showing a shift from starting materials, indicative of the formation of a new species. Crystals were then grown by slow evaporation and revealed by X-ray diffraction the tetrameric copper aggregate [Cu(C_6_F_5_)]_4_(DBTO)_2_ **6**, coordinated solely by two DBTO ligands (see Figure 1). The copper tetramer is nearly perfectly planar, with an elongated structure characteristic of tetranuclear copper species coordinated with two donor ligands. Pentafluorophenyl groups present interesting behavior: two are π-bonding with the DBTO moiety and lie in the plane of the Cu_4_ ring, whereas the other two are located above and under the Cu_4_ plane.

**Figure 2:**
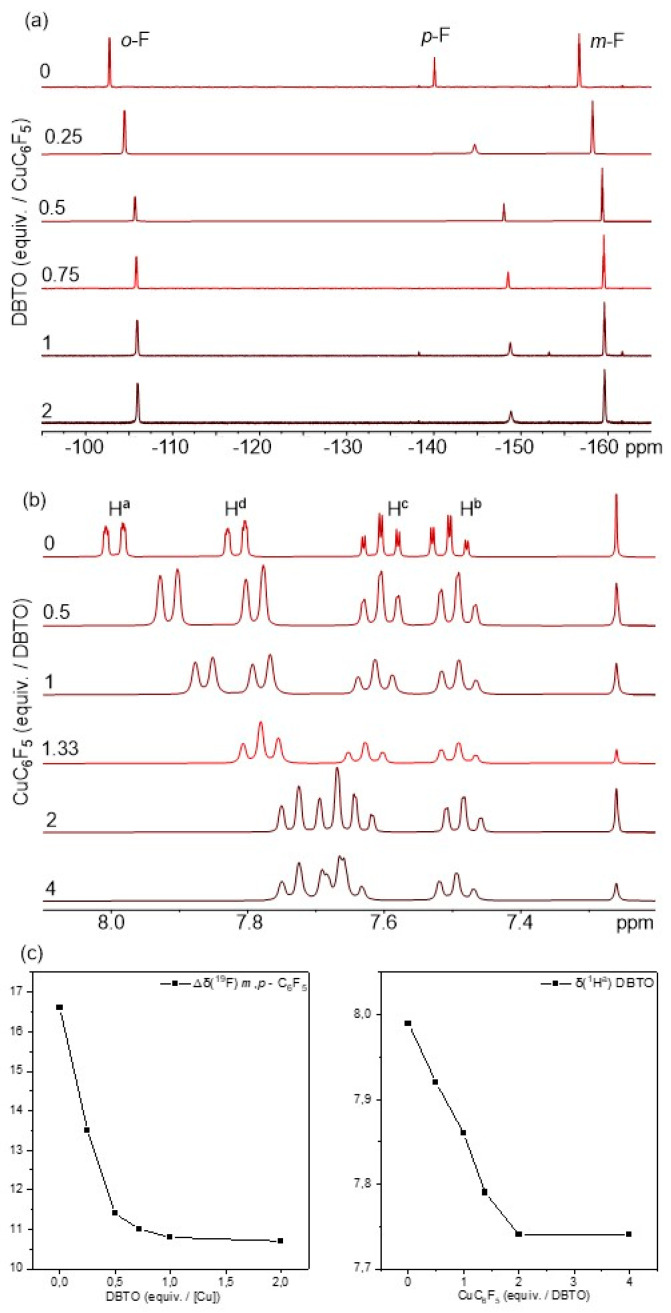
(**a**) ^19^F NMR spectra for the titration of the complexation of **5** with DBTO. (**b**) ^1^H NMR spectra for the titration of the complexation of DBTO with **5**. (**c**) NMR data plot for the titration of the formation of the complex **6**.

The use of DMSO as a ligand has been known to yield the monomeric complex Cu(C_6_F_5_)(DMSO) [21], with the observed metal/ligand ratio in its crystal state rather unexpected. This result prompted us to investigate its stoichiometry in solution. Similarly, to the case of Jäkle and coworkers with pyridine ligands, only one set of signals is observed by NMR, regardless of the equivalents of DBTO used (0.25 to 2 equiv. per CuC_6_F_5_), clearly stating the presence of a dynamic coordination equilibrium [17]. Moreover, both proton and fluorine NMR presented a noticeable shift depending on the equivalents of ligand used (see Figure 2a,b). A titration was thus carried out, plotting both the difference between the ^19^F shift of the fluorine *para* and the *meta* of the C_6_F_5_ moiety, Δδ(^19^F)*m*,*p*-C_6_F_5_, characteristic of the aggregation state of the copper complex [17,21] and the ^1^H shift of the proton H^a^ of DBTO, δ(^1^H^a^) DBTO (see Figure 2c).

Δδ(^19^F)*m*,*p*-C_6_F_5_ quickly diminishes upon adding up to 0.5 equivalents of DBTO, clearly showing the formation of **6**, and evolves only sparingly when more ligand is added. Known values of Δδ(^19^F)*m*,*p*-C_6_F_5_ above 10 ppm reported in the literature are consistent with [Cu(C_6_F_5_)]_4_L_2_ type complexes in solution, thus suggesting that **6** is preferentially formed in solution. More importantly, the compound does not undergo breakup of the tetramer, even upon adding excess ligand. On the other hand, the data obtained upon analyzing the ^1^H shifts also revealed a clear tendency, providing a regular shift upfield upon adding up to 2 equivalents of Cu(C_6_F_5_) **5**, upon which **6** is formed, whereas further additions led to a plateau, suggesting that the monoligated complex [Cu(C_6_F_5_)]_4_(DBTO) formation is unfavored in these conditions. It is noteworthy that the shift towards the upfield region is not consistent with what was observed in the case of pyridine derivatives, but can be rationalized thanks to the X-ray diffraction structure, showing that H^a^ lie in the shielding region of a C_6_F_5_ substituent.

Finally, a variable temperature NMR was recorded between −30 and 50 °C using a Cu(C_6_F_5_)/DBTO ratio of 1:1 (see S2 Supplementary Information). Interestingly, lowering the temperature provided the same Δδ(^19^F)*m*,*p*-C_6_F_5_ value as that obtained when mixing Cu(C_6_F_5_)/DBTO in a ratio of 4:2, whereas heating the solution led to lower values. These data thus suggest again that the complex stoichiometry exhibits a dynamic equilibrium with a preferential ratio of 4:2 and that the equilibrium can shift slightly towards the incorporation of additional ligands upon warming in solution.

### 2.2. Synthesis and Photoreactivity of Complexes [Cu(C_6_F_5_)]_4_(Tol_2_SO)_2_ and [Cu(C_6_F_5_)]_4_(Anthra_2_SO)_2_

**Figure 3:**
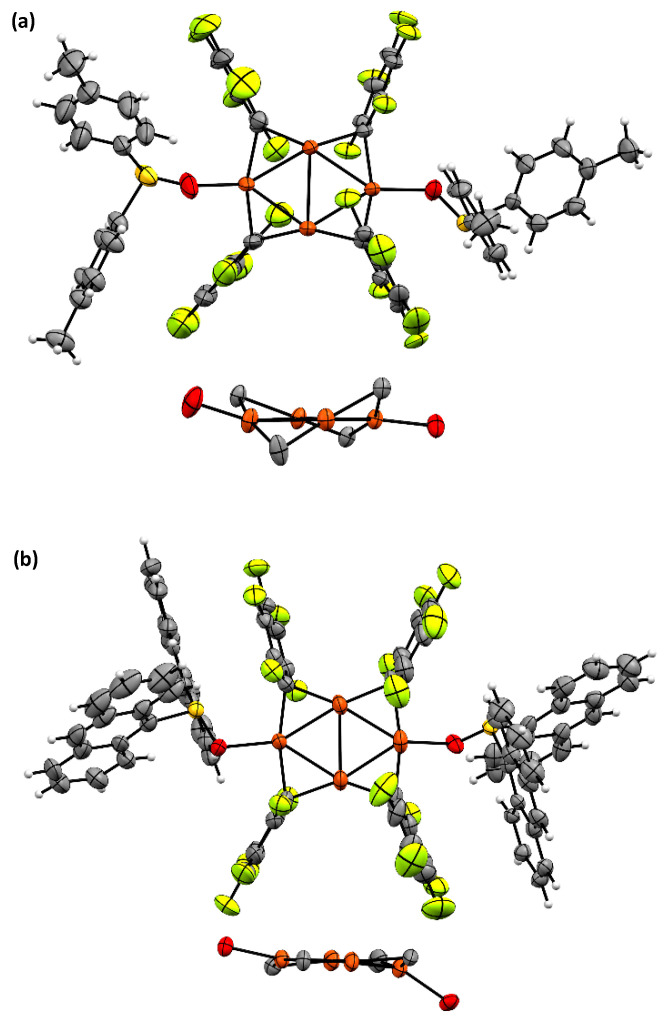
ORTEP view of X-ray structure of (**a**) [Cu(C_6_F_5_)]_4_(Tol_2_SO)_2_ **7** and (**b**) [Cu(C_6_F_5_)]_4_(Anthra_2_SO)_2_ **8** at 50% probability, and their corresponding view of the Cu_4_ ring coordination structure. Three cocrystallized molecules of benzene were omitted for clarity in complex **8**.

With the aim to further study the photoreactivity of biarylsulfoxide–CuC_6_F_5_, two new complexes, [Cu(C_6_F_5_)]_4_(Tol_2_SO)_2_ **7** and [Cu(C_6_F_5_)]_4_(Anthra_2_SO)_2_ **8**, were synthesized, using, respectively, bis-*p*-tolylsulfoxide (Tol_2_SO) and the photoreactive bis-anthracenylsulfoxide **3**. Pleasingly, X-ray diffraction analysis shown that even the strongly sterically demanding bis-anthracenylsulfoxide ligand yielded similar coordination behavior to DBTO and Tol_2_SO (see Figure 3).

In order to start probing the photoreactivity of the biarylsulfoxides within the coordination sphere of the copper, a quick survey of the UV-vis absorbance of the corresponding complexes was undertaken, showing a bathochromic shift up to the visible region of the main absorption features of the ligands (see S3 Supporting Information), thus encouraging us to study the photoreactivity using blue light (460 nm).

**Scheme 2:**
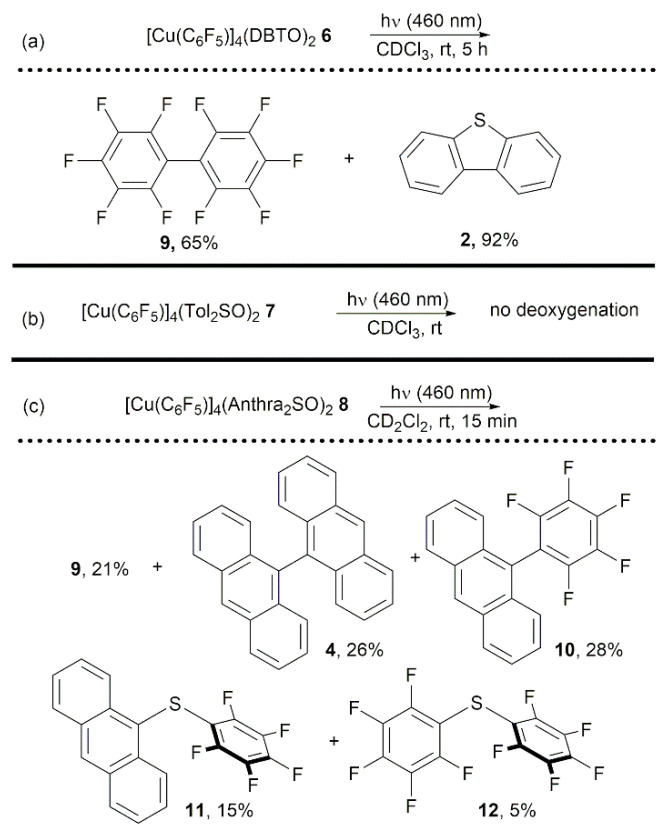
(**a**–**c**) Photoreactivity of biarylsulfoxide–copper(I) complexes **6**–**8**.

Pleasingly, the DBTO complex **6**, prepared in situ in a CuC_6_F_5_/DBTO 4:2 stoichiometry, exhibited photoreactivity (see Scheme 2a). Indeed, upon irradiating **6** with a blue light LED for 5 h, DBT **2** was cleanly furnished in 92% conversion, alongside 65% of (C_6_F_5_)_2_ **9** obtained as the sole product observed in ^19^F NMR, and Cu_2_O (see S5 Supporting Information). The deoxygenation process is in line with what is known for DBTO upon irradiation with UV light but can be promoted at a much energetically weaker wavelength thanks to the copper coordination. It is noteworthy that DBTO is unreactive in the same reaction conditions, whereas **5** undergoes slow photolysis yielding **9** in only 6% conversion. Importantly, when the solution containing a 1:1 stoichiometry was irradiated, 48% of DBT was obtained while 43% DBTO remained in solution, clearly stating that **6** is the photoreactive species of the reaction media. Encouraged by these results and hoping to extend the deoxygenation process to another biarylsulfoxide, the photoreactivity of **7** was investigated. However, no deoxygenated product was observed using blue light, and only the slow formation of **9** was observed (see Scheme 2b).

Finally, a solution of complex **8** was irradiated under blue light and yielded an unexpected range of products, with a full conversion obtained within 15 min of irradiation (see Scheme 2c). Firstly, as expected, bianthryl **4** and decafluorobiphenyl **9** were obtained in 26% and 21% NMR yields, respectively. Surprisingly, two other products arising from a cross coupling between C_6_F_5_ and both the anthryl and thioanthryl moiety, identified in ^1^H and ^19^F NMR as **10** and **11**, were obtained in 28% and 15% NMR yields^‡^, respectively. Furthermore, **12**, arising from an unexpected sulfur transfer of the bis-anthracenylsulfoxide to the C_6_F_5_ moiety, was formed in a 5% yield.

## 3. Materials and Methods

### 3.1. Reagents and Solvents

Unless otherwise noted, reagents were purchased from commercial suppliers and used directly without further purification. Unless indicated, technical grade solvents were purchased from commercial suppliers and used without further purification. CDCl_3_ and CD_2_Cl_2_ were dried and kept over activated 4 Å molecular sieves and degassed by the freeze–pump–thaw technique. All water was deionized before use. Unless stated, all reactions were carried out in Schlenk glassware under an inert atmosphere using either a standard Schlenk-line technique or an argon-filled glovebox. “Room temperature” can vary between 18 °C and 25 °C. 

9,9′-Dianthryl sulfoxide [8] and 9-(anthracen-9-yldisulfanyl)anthracene [22] were prepared according to reported procedures.

### 3.2. Analysis and Characterization 

Analytical thin layer chromatography (TLC) was performed on Merck aluminum-backed silica gel 60 F254 plates. Developed TLC plates were visualized by ultraviolet (UV) irradiation (254 nm). Column chromatography was carried out using Merk silica gel 60 Å, 220–440 mesh. Fourier transform infrared spectrometry (FTIR) was carried out using a Cary 630 FTIR using an attenuated total reflection (ATR) attachment and peaks were reported in terms of the frequency of absorption (cm^–1^). ^1^H, ^13^C, and ^19^F NMR spectra were recorded on Brucker Avance II 300 MHz, Avance III HD 400 MHz, and Avance I and II 500 MHz spectrometers (Brucker, Karlsruhe, Germany). Chemical shifts were expressed in parts per million (ppm), with residual solvent signals as an internal reference (^1^H and ^13^C{^1^H}). ^19^F NMR chemical shifts were reported in ppm relative to CFCl_3_. Coupling constants (*J*) were given in Hertz (Hz). The ^1^H NMR spectra were reported as follows: δ (multiplicity, coupling constant *J*, number of protons). Single-crystal X-ray data were collected at low temperature (193(2)K) on a Bruker APEX II Quazar diffractometer equipped with a 30 W air-cooled microfocus source (6) or on a Bruker D8 VENTURE diffractometer equipped with a PHOTON III detector (7 and 8), using MoKα radiation (λ = 0.71037 Å). The structures were solved by intrinsic phasing method [23] and refined by the full-matrix least-squares method on F2 [24]. All non-H atoms were refined with anisotropic displacement parameters and all the hydrogen atoms were refined isotropically at calculated positions using a riding model.

### 3.3. Synthesis of Copper–Biarylsulfoxide Complexes

[Cu(C_6_F_5_)]_4_(DBTO)_2_ **6**:


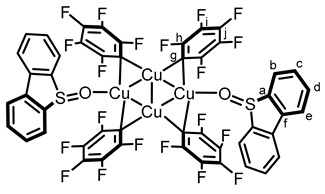


Dibenzothiophene-*S*-oxide (10.1 mg, 0.05 mmol) and pentafluorophenyl copper (23.0 mg, 0.1 mmol) were weighted in an amber-glass vial before adding CDCl_3_ (1 mL) and stirring for 5 min, providing the pure complex in quantitative yield. Slow evaporation of chloroform at RT provided crystals suitable for X-ray diffraction.

^1^H NMR (CDCl_3_, 600 MHz) δ_H_ 7.74 (d, *J* = 7.8 Hz, 4H, C_b_-H), 7.71 (d, *J* = 7.7 Hz, 4H, C_e_-H), 7.64 (td, *J* = 7.5, 1.1 Hz, 4H, C_d_-H), 7.49 (td, *J* = 7.5, 1.1 Hz, 4H, C_c_-H). ^13^C{^1^H} NMR (CDCl_3_, 151 MHz) δ_C_ 152.4 (dd, *J_C-F_* = 233.9, 23.8 Hz, C_h_), 142.4 (dd, *J_C-F_* = 253.8, 14.3 Hz, C_j_), 142.1 (C_a_), 136.7 (C_f_), 136.3 (dddd, *J_C-F_* = 256.6, 25.0, 12.7, 5.9 Hz, C_i_), 133.5 (C_d_), 130.1 (C_c_), 127.4 (C_b_), 122.1 (C_e_), 103.7 (t, *J_C-F_* = 57.2 Hz, C_g_). ^19^F NMR (CDCl_3_, 282 MHz) δ_F_ −105.73 (dd, *J* = 30.4, 11.1 Hz), −148.18 (t, *J* = 20.5 Hz), −159.41 (td, *J* = 20.6, 20.2, 10.2 Hz). FTIR (neat) νmax/cm^−1^ 3051, 2956, 2920, 2851, 1627, 1498, 1444, 1431, 1331, 1256, 1126, 1066, 1053, 1023, 988, 954, 753, 712.

[Cu(C_6_F_5_)]_4_(Tol_2_SO)_2_ **7**:


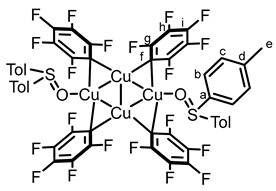


Bis-*p*-tolyl sulfoxide (11.5 mg, 0.05 mmol) and pentafluorophenyl copper (23.0 mg, 0.1 mmol) were weighted in an amber-glass vial before adding CDCl_3_ (1 mL) and stirring for 5 min, providing the pure complex in quantitative yield. Layering the chloroform solution with pentane yielded crystals suitable for X-ray diffraction upon standing.

^1^H NMR (CDCl_3_, 500 MHz) δ_H_ 7.21 (d, *J* = 7.8 Hz, 8H, C_b_-H), 7.15 (d, *J* = 8.4 Hz, 8H, C_c_-H), 2.36 (s, 12H, C_e_-H). ^13^C{^1^H} NMR (CDCl_3_, 126 MHz) δ_C_ 152.6 (dd, *J_C-F_* = 233.9, 24.2 Hz, C_g_), 143.0 (C_a_), 142.5 (d, *J_C-F_* = 253.9 Hz, C_i_), 139.0 (C_d_), 136.6 (ddd, *J_C-F_* = 256.9, 30.8, 12.9 Hz, C_h_), 130.4 (C_c_), 124.8 (C_b_), 104.4 (t, *J_C-F_* = 58.7 Hz, C_f_), 21.5 (C_e_). ^19^F NMR (CDCl_3_, 282 MHz) δ_F_ −105.42 (d, *J* = 20.2 Hz, C_g_-F), −149.06 (t, *J* = 19.9 Hz, C_i_-F), −160.03 (ddt, *J* = 28.2, 18.0, 9.0 Hz, C_h_-F). FTIR (neat) νmax/cm^−1^ 2961, 2928, 2853, 1631, 1599, 1496, 1448, 1431, 1332, 1258, 1068, 1053, 982, 954, 805, 756, 704.

[Cu(C_6_F_5_)]_4_(Anthra_2_SO)_2_ **8**:


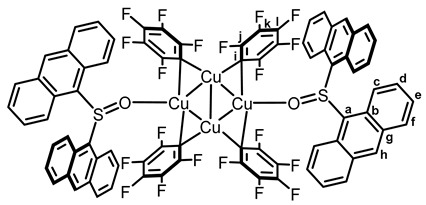


9,9′-Dianthryl sulfoxide (20.1 mg, 0.05 mmol) and pentafluorophenyl copper (23.0 mg, 0.1 mmol) were weighted in an amber-glass vial before adding CD_2_Cl_2_ (2 mL) and stirring for 5 min, providing the pure complex in quantitative yield. Applying this exact protocol in CDCl_3_ led to precipitation of the complex, whereas using C_6_D_6_ provided crystals suitable for X-ray diffraction upon standing.

^1^H NMR (CD_2_Cl_2_, 600 MHz) δ_H_ 8.80 (dd, *J* = 9.0, 0.5 Hz, 8H, C_c_-H), 8.55 (s, 4H, C_h_-H), 7.99 (ddd, *J* = 8.4, 0.8, 0.8 Hz, 8H, C_f_-H), 7.45 (ddd, *J* = 8.2, 6.6, 1.0 Hz, 8H, C_e_-H), 7.40 (ddd, *J* = 8.8, 6.6, 1.4 Hz, 8H, C_d_-H). ^13^C{^1^H} NMR (CD_2_Cl_2_, 151 MHz) δ_C_ 152.8 (dd, *J_C-F_* = 234.6, 24.1 Hz, C_j_), 142.5 (d, *J_C-F_* = 252.1 Hz, C_l_), 136.7 (ddd, *J_C-F_* = 255.5, 28.5, 11.7 Hz, C_k_), 134.1, 131.5, 130.9, 130.1 (C + C_a_), 128.7, 126.0, 122.6, 104.5 (broad, C_i_). ^19^F NMR (CD_2_Cl_2_, 282 MHz) δ_F_ −106.08 (d, *J* = 20.5 Hz, C_j_-F), −149.42 (t, *J* = 17.7 Hz, C_l_-F), −160.44 (ddt, *J* = 27.3, 16.8, 8.6 Hz, C_k_-F). FTIR (neat) νmax/cm^−1^ 3082, 3051, 2956, 2924, 2853, 1625, 1497, 1446, 1431, 1331, 1258, 1068, 1053, 990, 954, 898, 775, 729.

### 3.4. Photochemistry

The LEDs used were high-power Vision-EL (5W, λ = 460 ± 10 nm, 410 lm). Photochemistry experiments were carried out on an NMR scale. NMR conversions were obtained using 4,4′-difluoro-1,1′-biphenyl as internal standard allowing for quantitative ^1^H et ^19^F NMR, using in both cases a relaxation delay (d1) of 20 seconds.

Prior to the photochemistry studies, it was checked that the internal standard 4,4′-difluoro-1,1′-biphenyl was not interfering with the coordination behavior of the biarylsulfoxide copper complexes. Indeed, it was shown by Jäkle et al. that CuC_6_F_5_ can coordinate to aromatic hydrocarbons [21]. The NMR shifts of the complexes with and without the internal standard were compared, showing no difference. We thus assumed that 4,4′-difluoro-1,1′-biphenyl could be used. Moreover, the photoreactivity was investigated with and without 4,4′-difluoro-1,1′-biphenyl, showing comparable results and thus excluding an impact of the internal standard on the photoreactivity.

#### 3.4.1. Irradiation of [Cu(C_6_F_5_)]_4_(DBTO)_2_ **6**

A freshly prepared 0.1 M solution of [Cu(C_6_F_5_)]_4_(DBTO)_2_ **6** in CDCl_3_ was placed in a Young tap NMR tube in an argon-filled glovebox. After adding 4,4′-difluoro-1,1′-biphenyl (19.0 mg, 0.1 mmol, 1.0 equiv.), the tube was sealed and removed from the glovebox before recording a t = 0 NMR data point. The NMR tube was then irradiated with a blue LED for 6 h, at which point full consumption of the complex was observed. Analysis of the NMR data showed 65% of (C_6_F_5_)_2_ **9** as the sole product in ^19^F NMR and 92% of DBT as the sole product in ^1^H NMR.

#### 3.4.2. Irradiation of [Cu(C_6_F_5_)]_4_(Tol_2_SO)_2_ **7**

Irradiation of a solution of [Cu(C_6_F_5_)]_4_(Tol_2_SO)_2_ **7** in CDCl_3_ led only to a slow degradation of the CuC_6_F_5_ moiety, furnishing 6% of (C_6_F_5_)_2_ **9** after 5 h of irradiation and no noticeable change in ^1^H NMR.

#### 3.4.3. Irradiation of [Cu(C_6_F_5_)]_4_(Anthra_2_SO)_2_ **8**

A freshly prepared 0.05M solution of [Cu(C_6_F_5_)]_4_(Anthra_2_SO)_2_ **8** in CD_2_Cl_2_ was placed in a Young tap NMR tube in an argon-filled glovebox. After adding 4,4′-difluoro-1,1′-biphenyl (19.0 mg, 0.1 mmol, 1.0 equiv.), the tube was sealed and removed from the glovebox before recording a t = 0 NMR data point. The NMR tube was then irradiated with a blue LED for 15 min, at which point full consumption of the complex was observed. Analysis of the ^19^F NMR data showed 21% of (C_6_F_5_)_2_ **9** [25], 28% of **10**, 15% of **11**, and 5% of **12**. The ^1^H NMR data revealed the products **10** and **11**, as well as 26% of the expected bianthryl **4**. 

### 3.5. X-ray Data

CCDC 2360088 (**6**), CCDC 2360089 (**7**), and CCDC 2360090 (**8**) contain the supplementary crystallographic data for this paper. These data can be obtained free of charge from the Cambridge Crystallographic Data Centre via www.ccdc.cam.ac.uk/data_request/cif (accessed on 11 July 2024).

## 4. Conclusions

In summary, three new biarylsulfoxide–copper(I) complexes are presented. These complexes present a dynamic coordination behavior, of which [Cu(C_6_F_5_)]_4_L_2_ is the main species both in solution and in the solid state. Finally, the photochemical properties of the biarylsulfoxides within the coordination sphere of the copper have been evaluated, allowing (1) the S–O bond cleavage of DBTO under visible light and (2) access to unprecedented cross-coupling reactions between the C_6_F_5_ moiety of the organocopper and the photoreactive Anthra_2_SO. Finally, we want to emphasize that the present method allows for the controlled photoinduced synthesis of Cu_2_O, which we believe may find applications in material science, as this compound is well known to exhibit interesting optical and electrical properties [26,27]. 

## Data Availability

Data are available on request from the authors.

## Supplementary Materials

The following supporting information can be downloaded at https://www.mdpi.com/article/10.3390/molecules29143332/s1: S1 Stoichiometry titration study data. S2 Variable-temperature ^19^F NMR of a solution containing a 1:1 Cu(C_6_F_5_)/DBTO ratio. S3 UV-vis absorbance of complexes **6–8**. S4 Photolysis of complex **8**. S5 X-ray diffraction studies. S6 NMR Spectra. S7 IR Spectra.
