# Supplementary material for: Pentafluorophenyl Copper–Biarylsulfoxide Complexes: Synthesis and Photoreactivity
Source: Molecules. 2024 Jul 16;29(14):3332. doi: 10.3390/molecules29143332 (PMC11280416; doi:10.3390/molecules29143332)

Pentafluorophenyl Copper-Biarylsulfoxide complexes: Synthesis and Photoreactivity

Valentin Magné,*[1] Romaric Lenk,[1] Sonia Mallet-Ladeira,[2] Eddy Maerten,[1] David Madec*[1]

[1] Laboratoire Hétérochimie Fondamentale et Appliquée (UMR 5069), Université de Toulouse, CNRS,
118 Route de Narbonne, CEDEX 09, 31062 Toulouse, France.

[2] Institut de Chimie de Toulouse (UAR 2599), 118 Route de Narbonne, CEDEX 09, 31062 Toulouse, France.

*Correspondence: valentin.magne@univ-tlse3.fr ; david.madec@univ-tlse3.fr

Table of Contents

General methods 3

S1 Stoichiometry titration study 4

S2 Variable-temperature ^{19}F NMR of a solution containing a 1:1 $Cu(C_6F_5)$:DBTO ratio. 5

S3 UV-vis absorbance of complexes **6**-**8** 6

S4 Photolysis of complex 8: 8

S5 X-ray diffraction studies 11

S6 NMR Spectra 65

S7 IR Spectra 78

General methods

Reagents and Solvents

Unless otherwise noted, reagents were purchased from commercial suppliers and used directly without further purification. Unless indicated, technical grade solvents were purchased from commercial suppliers and used without further purification. $CDCl_3$ and CD_2Cl_2 were dried and kept over activated 4Å molecular sieves, and degassed by freeze pump thaw technique. All water was deionised before use. Unless stated, all reactions were carried out in Schlenk glassware under inert atmosphere using either standard Schlenk-line technique or an argon-filled glovebox. 'Room temperature' can vary between 18 °C and 25 °C.

9,9′-Dianthryl sulfoxide[1] and 9-(anthracen-9-yldisulfanyl)anthracene[2] were prepared according to reported procedures.

Photochemistry

The LEDs used are high-power Vision-EL (5W, λ = 460±10 nm, 410 lm). Photochemistry experiments were carried on NMR scale using 4,4′-difluoro-1,1′-biphenyl as an internal standard for both ^{1}H and ^{19}F NMR, thus allowing for quantitative ^{1}H et ^{19}F NMR using in both case a relaxation delay (d1) of 20 seconds.

Analysis and Characterization

Analytical Thin Layer Chromatography (TLC) was performed on Merck aluminium-backed silica gel 60 F254 plates. Developed TLC plates were visualized by ultraviolet (UV) irradiation (254 nm). Column chromatography was carried using Merk silica gel 60 Å, 220 - 440 mesh. Fourier Transform Infrared Spectrometry (FTIR) was carried out using a Cary 630 FTIR using an Attenuated Total Reflection (ATR) attachment and peaks are reported in terms of frequency of absorption (cm^{-1}). High Resolution Mass Spectrometry HRMS were acquired using a *GCT Premier CAB109* TOF mass spectrometer equipped with DCI-CH_4 ionization. HRMS data were quoted to four decimal places (0.1 mDa). All NMR spectra were recorded on either a Bruker AV 300, Bruker AV 500 or Bruker AV 600 and are internally referenced to residual solvent signals ($CDCl_3$ is referenced at δ 7.26 and 77.16 for ^{1}H and ^{13}C NMR respectively, CD_2Cl_2 is referenced at δ 5.32 and 53.84 for ^{1}H and ^{13}C NMR respectively). All NMR chemical shifts (δ) were reported in parts per million (ppm) and coupling constants (*J*) are given in Hertz (Hz). The ^{1}H NMR spectra are reported as follows: δ (multiplicity, coupling constant *J*, number of protons.)

[1] Christensen, P.R.; Patrick, B.O.; Caron, É.; Wolf, M.O. Oxidation-State-Dependent Photochemistry of Sulfur-Bridged Anthracenes. *Angew. Chem. Int. Ed.* **2013**, *52*, 12946–12950. https://doi.org/10.1002/anie.201306236.

[2] Akihiko, I.; Norio, N.; Yasunaka, T. 5,9b-dihydro-5,9b-banzonaphtho[1,2-b] Chalcogenophene Derivative, and Production Method of the Same. JP2012041295A, 1 March 2013.

S1 Stoichiometry titration study

$CDCl_3$ solutions containing variable equivalents of dibenzothiophene-*S*-oxide were prepared in an argon filled glovebox before recording the corresponding ^{19}F and ^{1}H NMR.

^{19}F and ^{1}H NMR data for **figure 2** of the main document:

DBTO (equiv. / CuC_6F_5)	$\delta(^{19}F)$ *o*-C_6F_5	$\delta(^{19}F)$*p*-C_6F_5	$\delta(^{19}F)$*m*-C_6F_5	$\Delta\delta(^{19}F)$*m,p* -C_6F_5
0	-102,8	-140,07	-156,7	16,63
0,25	-104,5	-144,67	-158,23	13,56
0,5	-105,7	-148,04	-159,36	11,32
0,72	-105,8	-148,52	-159,5	10,98
1	-105,9	-148,77	-159,63	10,86
2	-106	-148,86	-159,64	10,78

CuC_6F_5 (equiv. / DBTO)	$\delta(^{1}H^{a})$ DBTO
0	7,99
0,5	7,92
1	7,86
1,33	7,79
2	7,74
4	7,74

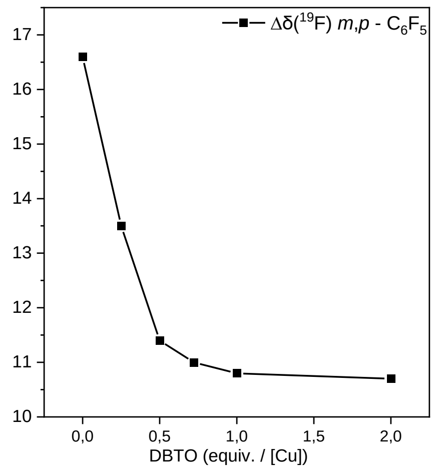

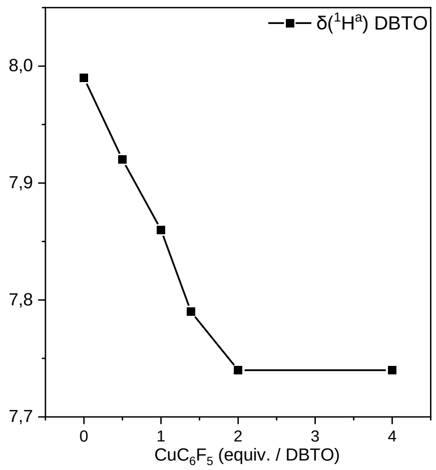

S2 Variable-temperature ^{19}F NMR of a solution containing a 1:1 $Cu(C_6F_5)$:DBTO ratio.

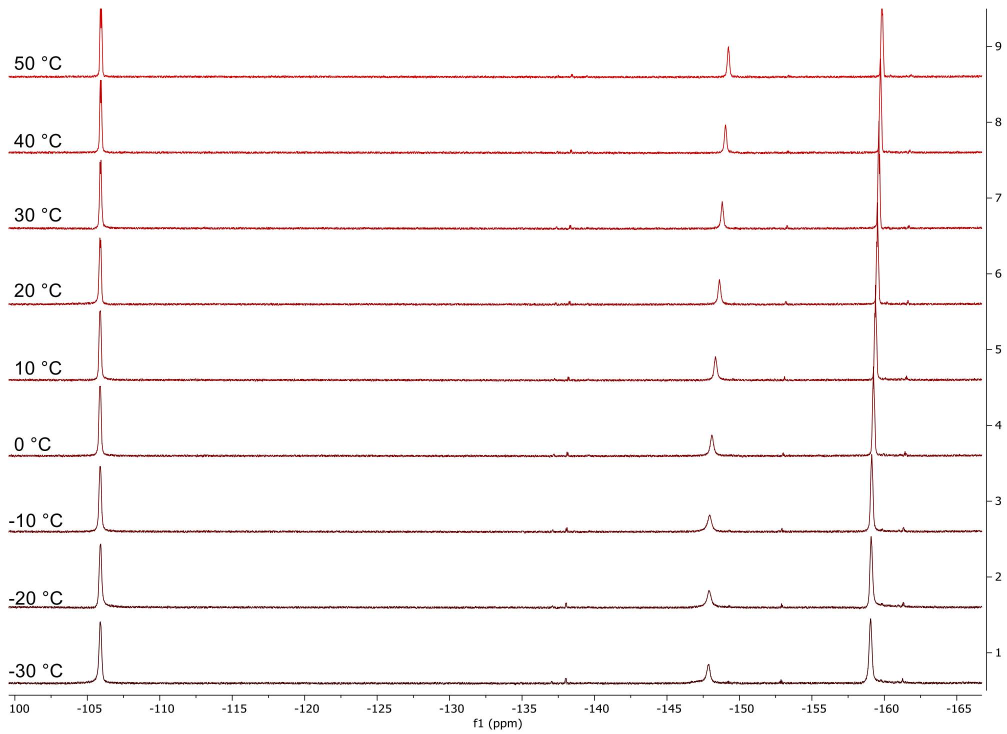

Temp (°C)	δ(Fo)	δ(Fp)	δ(Fm)	$\Delta\delta(^{19}F)$*m*,*p*-C_6F_5
-30	-105,93	-147,87	-159,05	11,2
-20	-105,9	-147,91	-159,09	11,2
-10	-105,89	-147,94	-159,13	11,2
0	-105,89	-148,1	-159,25	11,2
10	-105,88	-148,35	-159,39	11
20	-105,86	-148,62	-159,53	10,9
30	-105,91	-148,82	-159,62	10,8
40	-105,93	-149,04	-159,74	10,7
50	-105,94	-149,24	-159,84	10,6

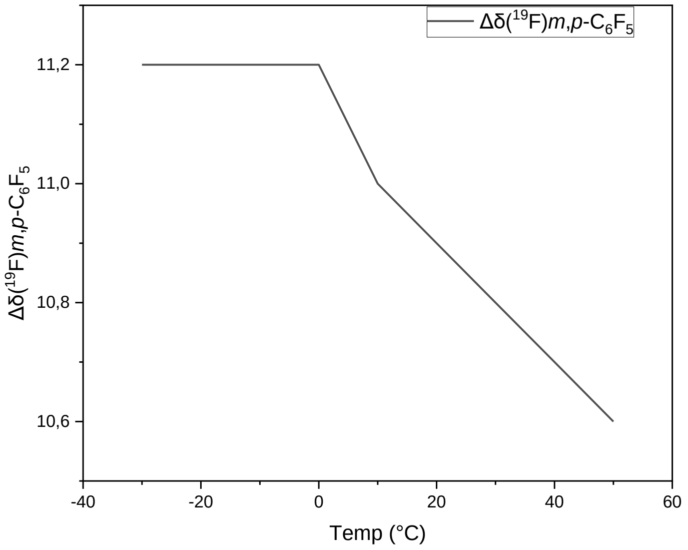

S3 UV-vis absorbance of complexes **6-8**

<u>UV data:</u>

Copper biarylsulfoxide complexes underwent hydrolysis under diluted conditions, the decision was thus taken to use concentrated solutions, revealing the foot of the absorption peak. All data presented here was recorded in dry and degassed DCM in quartz cuvettes, prepared in an argon-filled glovebox.

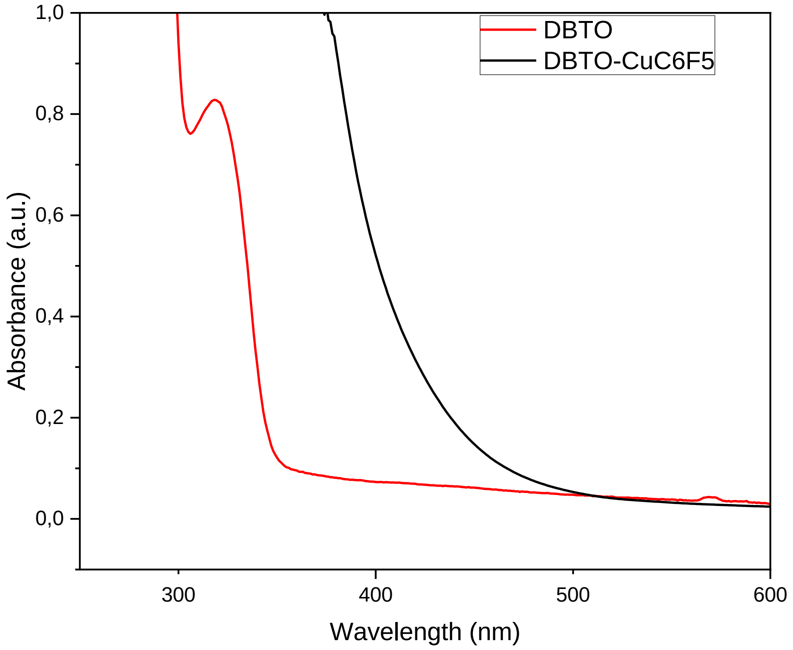

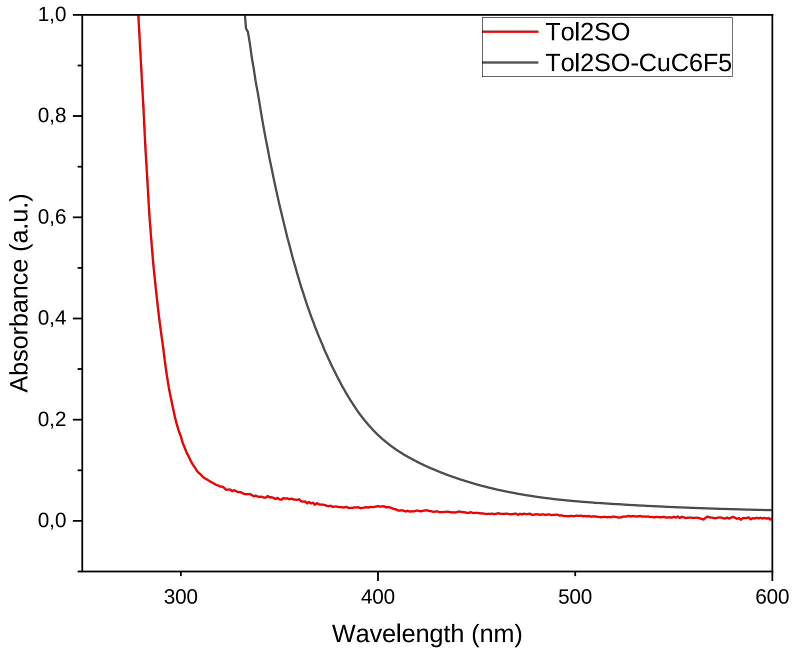
Tol2SO
Tol2SO-CuC6F5
Absorbance (a.u.)
1,0
0,8
0,6
0,4
0,2
0,0
300
400
500
600
Wavelength (nm)

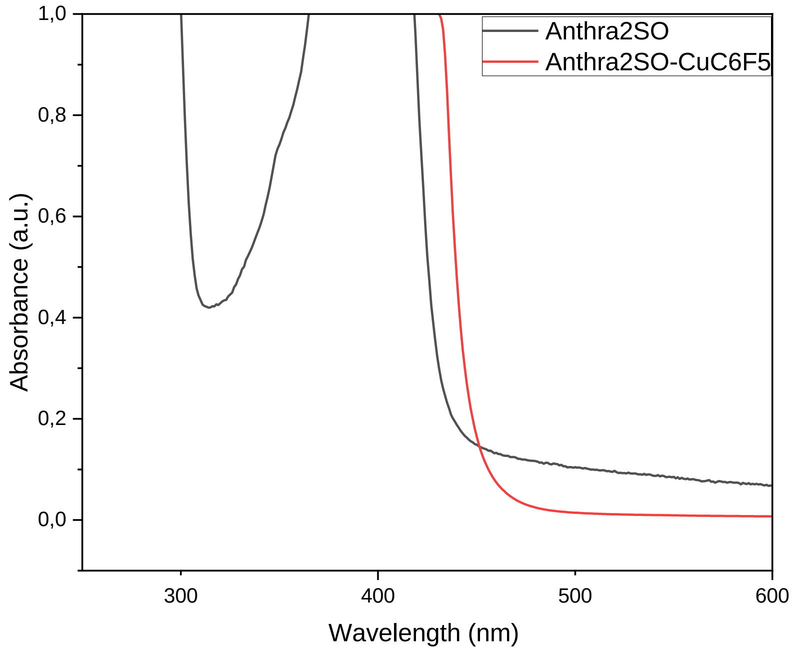
Anthra2SO
Anthra2SO-CuC6F5
Absorbance (a.u.)
1,0
0,8
0,6
0,4
0,2
0,0
300
400
500
600
Wavelength (nm)

S4 Photolysis of complex 8:

In order to ascertain the identification of the products arising from the photolysis of complex **8**, **4** was compared with existing data, **12** was bought, and **9**, **10**, and **11** were independently synthesized and their NMR in CD_2Cl_2 recorded.

9,9'-Bianthryl 4:

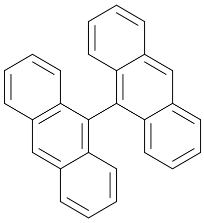

Reported by Wolf *et al.*.[1]

Perfluoro-1,1'-biphenyl 9:

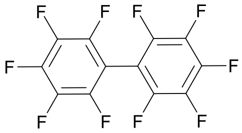

A clean ^{19}F NMR spectrum in CD_2Cl_2 was obtained irradiating a solution of **6** in CD_2Cl_2. Data consistent with the literature.[3]
^{19}F NMR (CD_2Cl_2, 282 MHz) δ_F -137.97 (dd, *J* = 21.2, 5.2 Hz), -150.78 (t, *J* = 20.8 Hz), -161.24 (td, *J* = 20.4, 19.7, 5.4 Hz).

9-(perfluorophenyl)anthracene 10:

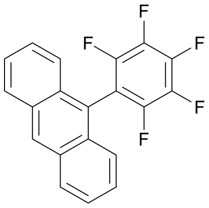

*n*BuLi (0.86 mL, 2.14 mmol, 2.5M, 1.1 equiv.) was added dropwise to a solution of 9-bromoanthracene (500 mg, 1.94 mmol, 1 equiv.) in Et_2O (10 mL) at 0°C. After stirring for 5 minutes, perfluorobenzene (0.25 mL, 2.14 mmol, 1.1 equiv.) was added in one portion and the reaction mixture was stirred overnight at room temperature. Water was added and the aqueous was extracted thrice with DCM, the combined organic layers were dried over $MgSO_4$ and evaporated to dryness. The crude mixture was filtered over silica eluting with 2%DCM/Pentane and the resulting solid was sublimed under high vacuum (0.1 mbar). 20 -> 80 °C: impurity + a little bit of desired product. 80 -> 100 °C: yields pure desired product (80 mg, 0.23 mmol, 12%).
^{1}H NMR ($CDCl_3$, 600 MHz) δ_H 8.64 (s, 1H), 8.13 – 8.08 (m, 2H), 7.56 – 7.44 (m, 6H). **^{13}C{^{1}H} NMR** ($CDCl_3$, 151 MHz) δ_C 145.1 (d, J_{C-F} = 247.9 Hz), 141.4 (dt, J_{C-F} = 255.3, 13.9 Hz), 138.1 (dt, J_{C-F} = 252.9, 13.1 Hz), 131.4, 130.7, 129.7, 129.1, 127.2, 125.7, 124.8, 119.8, 112.7 (t, J_{C-F} = 20.0 Hz). **^{19}F NMR** (CD_2Cl_2, 282 MHz) δ -139.04 – -139.23 (m), -154.47 – -154.70 (m), -162.11 – -162.40 (m).

[3] Hofer, M., Nevado, C. *Tetrahedron*, **2013**, 69, 5751-5757. https://doi.org/10.1016/j.tet.2013.04.029.

Anthracen-9-yl(perfluorophenyl)sulfane 11:

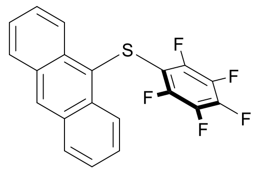

*n*BuLi (0.2 mL, 0.5 mmol, 2.5M, 1.0 equiv.) was added dropwise to a solution of iodopentafluorobenzene (80 µL, 0.6 mmol, 1.2 equiv.) in Et_2O (10 mL) kept at -90°C. The temperature was risen up to -60°C over 1 hour then cooled again to -80°C before adding 9-(anthracen-9-yldisulfanyl)anthracene (209 mg, 0.5 mmol, 1.0 equiv.) portionwise under a flux of argon. The temperature was slowly risen up to room temperature overnight at which point aqueous NaOH 2M was added (20 mL). The crude mixture was extracted thrice with DCM, dried over $MgSO_4$, and evaporated to dryness. The crude mixture was then purified over silica gel chromatography (100% Pentane) yielding the desired product (135 mg, 0.36 mmol, 72%).
Caution: pentafluorobenzene lithium is known to be explosive at temperatures above -40°C, this species thus must be handled with care.
^{1}H NMR (CD_2Cl_2, 600 MHz) δ_H 8.85 (dd, *J* = 8.9, 0.9 Hz, 2H), 8.60 (s, 1H), 8.06 (ddt, *J* = 8.4, 1.4, 0.7 Hz, 2H), 7.65 (ddd, *J* = 8.9, 6.5, 1.3 Hz, 2H), 7.54 (ddd, *J* = 8.4, 6.5, 1.1 Hz, 2H). **^{13}C{^{1}H} NMR** (CD_2Cl_2, 151 MHz) δ_C 147.4 (dddd, $J_{C\text{-}F}$ = 246.9, 11.4, 9.2, 4.1 Hz), 141.1 (dtt, $J_{C\text{-}F}$ = 253.8, 13.4, 5.0 Hz), 139.2 – 137.1 (m), 134.84, 132.2, 131.4, 129.5, 128.0, 126.4, 125.9, 123.8, 111.7 (td, *J* = 20.0, 4.5 Hz). **^{19}F NMR** (CD_2Cl_2, 282 MHz) δ_F -133.86 – -134.40 (m), -154.98 (tt, *J* = 20.7, 1.9 Hz), -161.54 – -162.35 (m).

bis(perfluorophenyl)sulfane 12:

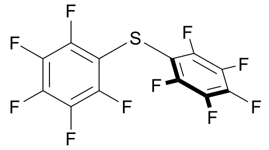

Commercial sample, **^{19}F NMR** (CD_2Cl_2, 282 MHz) δ_F -132.45 (ddt, *J* = 18.0, 6.7, 3.3 Hz), -150.56 (tt, *J* = 20.8, 3.4 Hz), -160.57 – -160.84 (m).

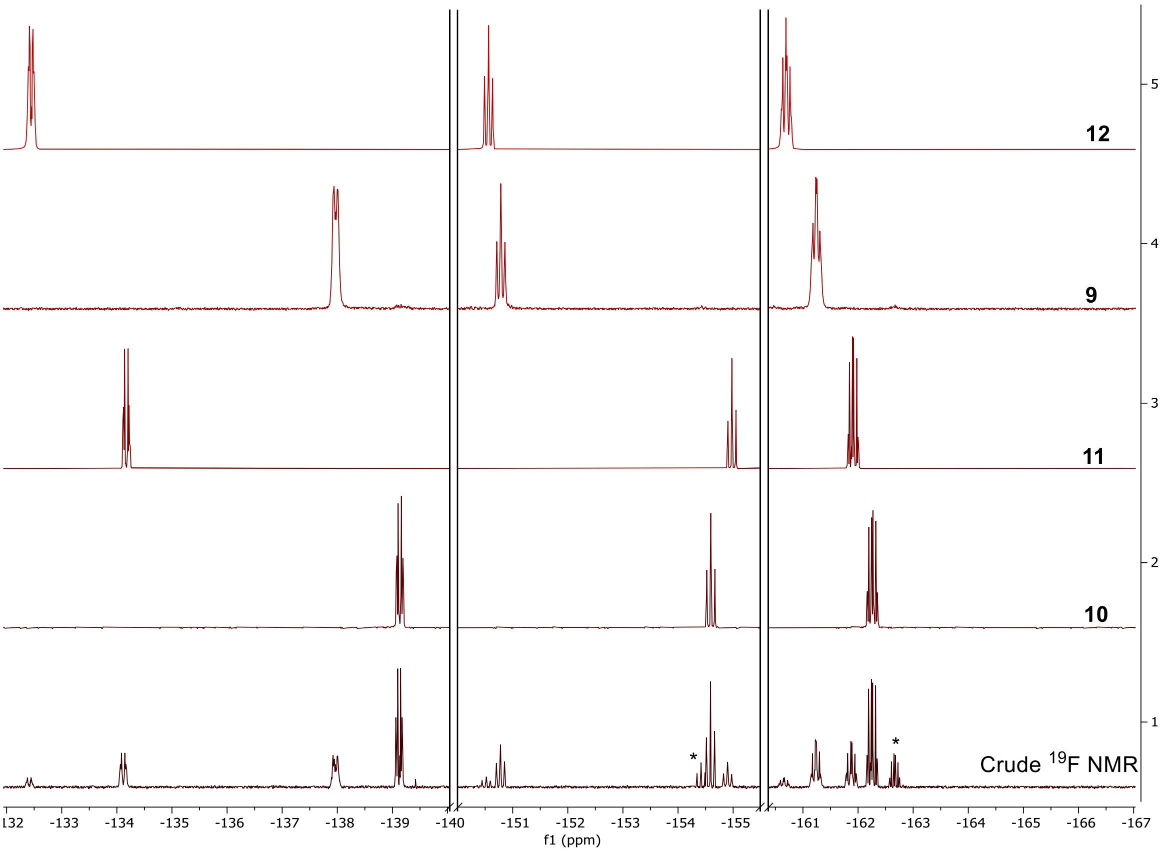

^{19}F NMR spectrum of the crude reaction mixture after irradiating **8** with a blue LED for 15 minutes (bottom) overlaid with the ^{19}F NMR of pure samples of **10**, **11**, **9**, and **12**. Peaks marked with an asterisk (*) are trace amounts of HC_6F_5 arising from partial hydrolysis of the $[Cu(C_6F_5)]_4$, which are already present prior to irradiation.

S5 X-ray diffraction studies

Cu$_2$O powder diffraction:

Subsequently to the irradiation of a freshly prepared 0.1M solution of [Cu(C$_6$F$_5$)]$_4$(DBTO)$_2$ **6** in CDCl$_3$, the reaction mixture was brought in a glovebox before being transferred in a vial and diluted with dry and degassed CHCl$_3$ (2 mL). A greenish solid slowly sedimented over time. The supernatant was removed and the resulting powder was washed following the same protocol twice before drying under high vacuum yielding a free-flowing powder. This product was then transferred in a powder Xray capillary and sealed under argon before recording powder Xray data.

Graphics

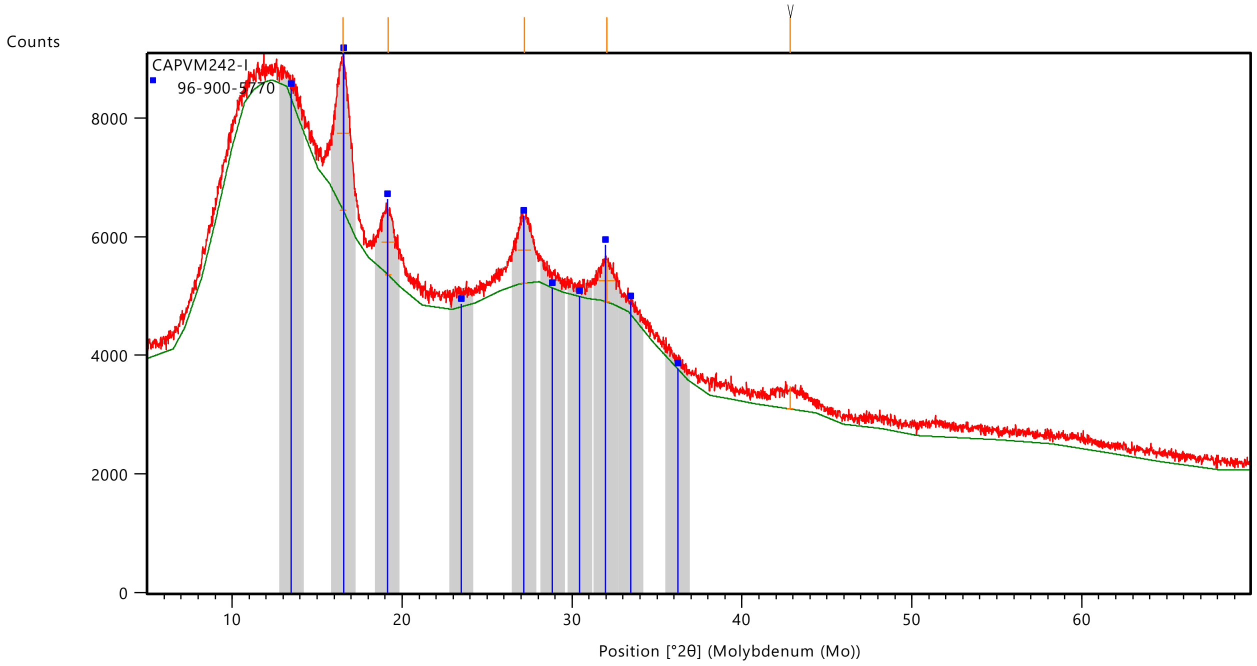

Peak List

Pos. [°2θ]	Height [cts]	d-spacing [Å]	Rel. Int. [%]
16,5269	2582,48	2,47260	100,00
19,1653	1083,71	2,13476	41,96
27,1795	1117,34	1,51244	43,27
32,0504	707,64	1,28731	27,40
42,8393	380,11	0,97115	14,72

Pattern List

Visible	Ref.Code	Score	Compound Name	Displ.[°2θ]	Scale Fac.	Chem. Formula
*	96-900-5770	94	Cuprite	0,000	0,973	Cu4.00 O2.00

Xray structure of $[Cu(C_6F_5)]_4(DBTO)_2$ **6**

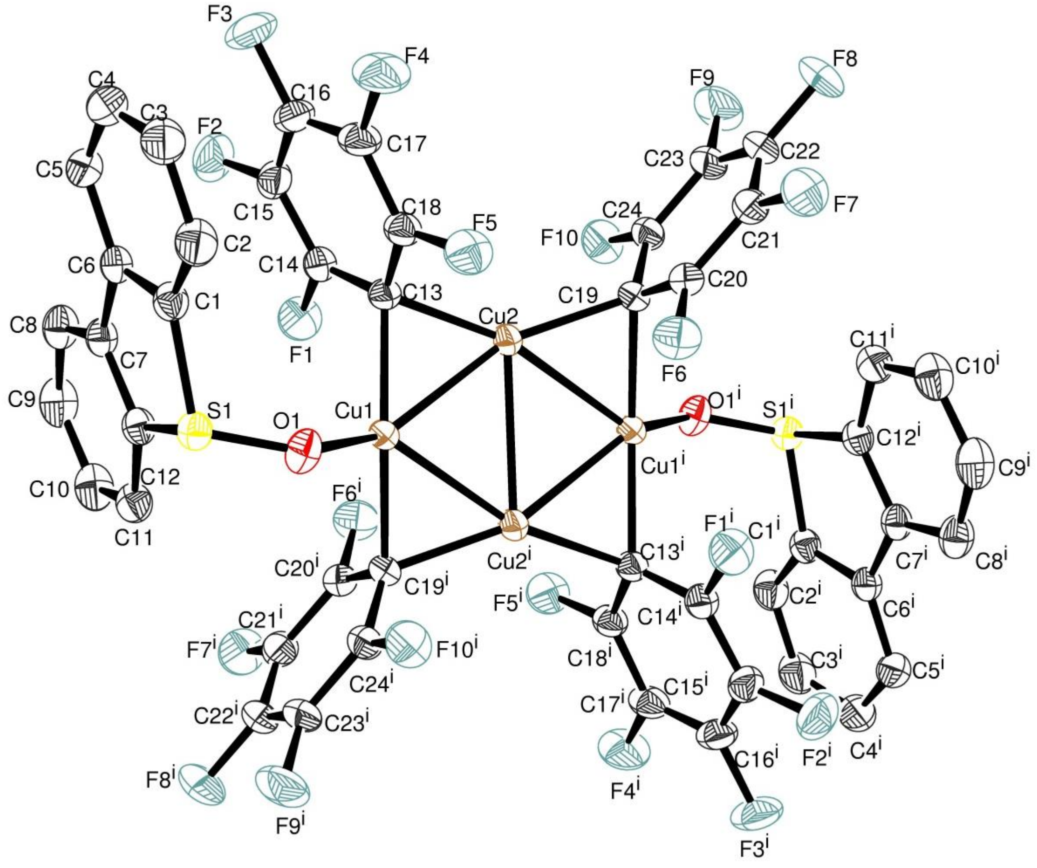

Figure S2 : Asymmetric Unit

Table S1. Crystal data and structure refinement for **6**.

Identification code	**6**
Empirical formula	C48 H16 Cu4 F20 O2 S2
Formula weight	1322.93
Temperature	193(2) K
Wavelength	0.71073 A
Crystal system, space group	Triclinic, P -1
Unit cell dimensions	a = 9.596(2) A alpha = 61.783(5) deg.
	b = 11.6757(14) A beta = 75.949(9) deg.
	c = 11.8142(15) A gamma = 75.121(8) deg.
Volume	1115.8(3) A^3

Z, Calculated density 1, 1.969 Mg/m^3

Absorption coefficient 2.101 mm^-1

F(000) 648

Crystal size 0.20 x 0.20x 0.160 mm

Theta range for data collection 2.580 to 37.689 deg.

Limiting indices -16<=h<=15, -19<=k<=19, -19<=l<=20

Reflections collected / unique 75804 / 11085 [R(int) = 0.0443]

Completeness to theta = 25.242 99.7 %

Refinement method Full-matrix least-squares on F^2

Data / restraints / parameters 11085 / 0 / 343

Goodness-of-fit on F^2 1.058

Final R indices [I>2sigma(I)] R1 = 0.0240, wR2 = 0.0690

R indices (all data) R1 = 0.0279, wR2 = 0.0711

Largest diff. peak and hole 0.464 and -0.545 e.A^-3

Table S2. Atomic coordinates (x 10^4) and equivalent isotropic displacement parameters (A^2 x 10^3) for **6**.
U(eq) is defined as one third of the trace of the orthogonalized Uij tensor.

	x	y	z	U(eq)
C(1)	-81(1)	2145(1)	7240(1)	26(1)
C(2)	-931(1)	2484(1)	6294(1)	36(1)
C(3)	-1083(2)	1485(1)	6024(1)	43(1)
C(4)	-406(2)	193(1)	6688(1)	41(1)
C(5)	448(1)	-140(1)	7639(1)	33(1)
C(6)	619(1)	848(1)	7915(1)	26(1)
C(7)	1450(1)	739(1)	8866(1)	25(1)
C(8)	2355(1)	-349(1)	9655(1)	33(1)
C(9)	3075(1)	-201(1)	10460(1)	38(1)
C(10)	2894(1)	1001(1)	10501(1)	37(1)
C(11)	1970(1)	2094(1)	9734(1)	31(1)
C(12)	1282(1)	1939(1)	8918(1)	25(1)
C(13)	3701(1)	2938(1)	5423(1)	24(1)
C(14)	4244(1)	1635(1)	6213(1)	27(1)
C(15)	3944(1)	558(1)	6168(1)	33(1)
C(16)	3102(1)	770(1)	5266(1)	36(1)
C(17)	2542(1)	2039(1)	4446(1)	34(1)
C(18)	2834(1)	3083(1)	4552(1)	28(1)
C(19)	6302(1)	5004(1)	2687(1)	24(1)
C(20)	5400(1)	5870(1)	1750(1)	27(1)
C(21)	5641(1)	6013(1)	488(1)	33(1)
C(22)	6841(1)	5247(1)	116(1)	37(1)
C(23)	7759(1)	4354(1)	1007(1)	35(1)
C(24)	7474(1)	4240(1)	2260(1)	28(1)
Cu(1)	3241(1)	4170(1)	6274(1)	23(1)
Cu(2)	5121(1)	4212(1)	4434(1)	25(1)
F(1)	5100(1)	1378(1)	7089(1)	41(1)
F(2)	4464(1)	-682(1)	6978(1)	50(1)
F(3)	2831(1)	-264(1)	5193(1)	54(1)
F(4)	1739(1)	2231(1)	3569(1)	53(1)
F(5)	2247(1)	4308(1)	3748(1)	42(1)
F(6)	4203(1)	6634(1)	2061(1)	40(1)
F(7)	4742(1)	6879(1)	-373(1)	50(1)
F(8)	7095(1)	5365(1)	-1094(1)	58(1)
F(9)	8918(1)	3604(1)	642(1)	58(1)
F(10)	8400(1)	3341(1)	3083(1)	42(1)
O(1)	1020(1)	4275(1)	6835(1)	31(1)
S(1)	110(1)	3241(1)	7835(1)	25(1)

Table S3. Bond lengths [A] and angles [deg] for **6**.

C(1)-C(2)	1.3896(14)
C(1)-C(6)	1.4054(13)
C(1)-S(1)	1.7889(10)
C(2)-C(3)	1.3941(17)
C(2)-H(2)	0.9500
C(3)-C(4)	1.392(2)
C(3)-H(3)	0.9500
C(4)-C(5)	1.3981(17)
C(4)-H(4)	0.9500
C(5)-C(6)	1.3931(14)
C(5)-H(5)	0.9500
C(6)-C(7)	1.4712(13)
C(7)-C(8)	1.3942(13)
C(7)-C(12)	1.3971(13)
C(8)-C(9)	1.3957(17)
C(8)-H(8)	0.9500
C(9)-C(10)	1.3913(18)
C(9)-H(9)	0.9500
C(10)-C(11)	1.3975(15)
C(10)-H(10)	0.9500
C(11)-C(12)	1.3899(13)
C(11)-H(11)	0.9500
C(12)-S(1)	1.7874(9)
C(13)-C(14)	1.3947(13)
C(13)-C(18)	1.3993(12)
C(13)-Cu(2)	2.0149(9)
C(13)-Cu(1)	2.0252(9)
C(14)-F(1)	1.3517(12)
C(14)-C(15)	1.3866(14)
C(15)-F(2)	1.3463(13)
C(15)-C(16)	1.3837(18)
C(16)-F(3)	1.3432(12)
C(16)-C(17)	1.3814(18)
C(17)-F(4)	1.3406(13)
C(17)-C(18)	1.3844(13)
C(18)-F(5)	1.3517(12)
C(19)-C(24)	1.3958(13)
C(19)-C(20)	1.3984(13)
C(19)-Cu(2)	1.9989(9)
C(19)-Cu(1)#1	2.0565(8)
C(20)-F(6)	1.3524(12)
C(20)-C(21)	1.3851(13)
C(21)-F(7)	1.3449(13)
C(21)-C(22)	1.3845(17)
C(22)-F(8)	1.3347(12)
C(22)-C(23)	1.3828(17)
C(23)-F(9)	1.3477(13)
C(23)-C(24)	1.3855(13)
C(24)-F(10)	1.3462(12)

Cu(1)-O(1)	2.0624(8)
Cu(1)-Cu(2)	2.4550(4)
Cu(1)-Cu(2)#1	2.4764(4)
Cu(2)-Cu(2)#1	2.6809(3)
O(1)-S(1)	1.5195(8)

C(2)-C(1)-C(6)	122.32(9)
C(2)-C(1)-S(1)	124.60(8)
C(6)-C(1)-S(1)	112.83(7)
C(1)-C(2)-C(3)	117.73(11)
C(1)-C(2)-H(2)	121.1
C(3)-C(2)-H(2)	121.1
C(4)-C(3)-C(2)	120.79(10)
C(4)-C(3)-H(3)	119.6
C(2)-C(3)-H(3)	119.6
C(3)-C(4)-C(5)	121.09(10)
C(3)-C(4)-H(4)	119.5
C(5)-C(4)-H(4)	119.5
C(6)-C(5)-C(4)	118.88(10)
C(6)-C(5)-H(5)	120.6
C(4)-C(5)-H(5)	120.6
C(5)-C(6)-C(1)	119.17(9)
C(5)-C(6)-C(7)	128.76(9)
C(1)-C(6)-C(7)	112.06(8)
C(8)-C(7)-C(12)	118.85(9)
C(8)-C(7)-C(6)	129.31(9)
C(12)-C(7)-C(6)	111.82(8)
C(7)-C(8)-C(9)	118.96(10)
C(7)-C(8)-H(8)	120.5
C(9)-C(8)-H(8)	120.5
C(10)-C(9)-C(8)	121.41(10)
C(10)-C(9)-H(9)	119.3
C(8)-C(9)-H(9)	119.3
C(9)-C(10)-C(11)	120.29(10)
C(9)-C(10)-H(10)	119.9
C(11)-C(10)-H(10)	119.9
C(12)-C(11)-C(10)	117.63(10)
C(12)-C(11)-H(11)	121.2
C(10)-C(11)-H(11)	121.2
C(11)-C(12)-C(7)	122.82(9)
C(11)-C(12)-S(1)	123.72(8)
C(7)-C(12)-S(1)	113.46(7)
C(14)-C(13)-C(18)	114.43(8)
C(14)-C(13)-Cu(2)	118.00(6)
C(18)-C(13)-Cu(2)	107.79(6)
C(14)-C(13)-Cu(1)	114.62(6)
C(18)-C(13)-Cu(1)	121.08(7)
Cu(2)-C(13)-Cu(1)	74.84(3)
F(1)-C(14)-C(15)	116.92(9)
F(1)-C(14)-C(13)	119.51(8)
C(15)-C(14)-C(13)	123.57(9)
F(2)-C(15)-C(16)	119.80(10)

F(2)-C(15)-C(14)	121.14(11)
C(16)-C(15)-C(14)	119.06(10)
F(3)-C(16)-C(17)	120.16(11)
F(3)-C(16)-C(15)	119.62(11)
C(17)-C(16)-C(15)	120.21(9)
F(4)-C(17)-C(16)	119.60(10)
F(4)-C(17)-C(18)	121.69(11)
C(16)-C(17)-C(18)	118.71(9)
F(5)-C(18)-C(17)	116.99(9)
F(5)-C(18)-C(13)	119.03(8)
C(17)-C(18)-C(13)	123.97(10)
C(24)-C(19)-C(20)	114.11(8)
C(24)-C(19)-Cu(2)	121.20(7)
C(20)-C(19)-Cu(2)	110.65(6)
C(24)-C(19)-Cu(1)#1	116.35(7)
C(20)-C(19)-Cu(1)#1	113.75(6)
Cu(2)-C(19)-Cu(1)#1	75.26(3)
F(6)-C(20)-C(21)	116.11(9)
F(6)-C(20)-C(19)	119.56(8)
C(21)-C(20)-C(19)	124.34(9)
F(7)-C(21)-C(22)	119.78(9)
F(7)-C(21)-C(20)	121.44(10)
C(22)-C(21)-C(20)	118.78(9)
F(8)-C(22)-C(23)	120.56(11)
F(8)-C(22)-C(21)	119.89(11)
C(23)-C(22)-C(21)	119.54(9)
F(9)-C(23)-C(22)	119.32(9)
F(9)-C(23)-C(24)	120.92(11)
C(22)-C(23)-C(24)	119.76(9)
F(10)-C(24)-C(23)	116.72(9)
F(10)-C(24)-C(19)	119.85(8)
C(23)-C(24)-C(19)	123.43(9)
C(13)-Cu(1)-C(19)#1	154.23(4)
C(13)-Cu(1)-O(1)	102.45(3)
C(19)#1-Cu(1)-O(1)	99.63(3)
C(13)-Cu(1)-Cu(2)	52.39(2)
C(19)#1-Cu(1)-Cu(2)	113.76(3)
O(1)-Cu(1)-Cu(2)	141.20(2)
C(13)-Cu(1)-Cu(2)#1	117.95(3)
C(19)#1-Cu(1)-Cu(2)#1	51.32(2)
O(1)-Cu(1)-Cu(2)#1	132.15(2)
Cu(2)-Cu(1)-Cu(2)#1	65.862(11)
C(19)-Cu(2)-C(13)	140.78(3)
C(19)-Cu(2)-Cu(1)	157.08(3)
C(13)-Cu(2)-Cu(1)	52.77(3)
C(19)-Cu(2)-Cu(1)#1	53.43(3)
C(13)-Cu(2)-Cu(1)#1	165.79(2)
Cu(1)-Cu(2)-Cu(1)#1	114.138(11)
C(19)-Cu(2)-Cu(2)#1	107.04(3)
C(13)-Cu(2)-Cu(2)#1	109.97(3)
Cu(1)-Cu(2)-Cu(2)#1	57.453(10)
Cu(1)#1-Cu(2)-Cu(2)#1	56.685(10)

S(1)-O(1)-Cu(1)	131.27(4)
O(1)-S(1)-C(12)	107.77(4)
O(1)-S(1)-C(1)	111.60(4)
C(12)-S(1)-C(1)	89.58(4)

Symmetry transformations used to generate equivalent atoms:
#1 -x+1,-y+1,-z+1

Table S4. Anisotropic displacement parameters (A^2 x 10^3) for **6**.
The anisotropic displacement factor exponent takes the form:
-2 pi^2 [h^2 a*^2 U11 + ... + 2 h k a* b* U12]

	U11	U22	U33	U23	U13	U12
C(1)	24(1)	30(1)	25(1)	-10(1)	-3(1)	-8(1)
C(2)	37(1)	41(1)	31(1)	-10(1)	-10(1)	-12(1)
C(3)	49(1)	54(1)	32(1)	-16(1)	-9(1)	-23(1)
C(4)	49(1)	48(1)	36(1)	-21(1)	2(1)	-26(1)
C(5)	37(1)	32(1)	33(1)	-16(1)	2(1)	-14(1)
C(6)	24(1)	28(1)	24(1)	-10(1)	1(1)	-8(1)
C(7)	22(1)	26(1)	24(1)	-9(1)	0(1)	-5(1)
C(8)	28(1)	28(1)	33(1)	-7(1)	-4(1)	-2(1)
C(9)	29(1)	39(1)	34(1)	-5(1)	-10(1)	-3(1)
C(10)	31(1)	46(1)	31(1)	-10(1)	-10(1)	-10(1)
C(11)	28(1)	36(1)	29(1)	-13(1)	-4(1)	-10(1)
C(12)	21(1)	28(1)	23(1)	-10(1)	-2(1)	-5(1)
C(13)	26(1)	27(1)	24(1)	-13(1)	-1(1)	-9(1)
C(14)	27(1)	29(1)	26(1)	-12(1)	0(1)	-7(1)
C(15)	34(1)	27(1)	36(1)	-15(1)	7(1)	-9(1)
C(16)	35(1)	40(1)	46(1)	-30(1)	10(1)	-18(1)
C(17)	32(1)	49(1)	37(1)	-28(1)	1(1)	-16(1)
C(18)	28(1)	34(1)	27(1)	-16(1)	-2(1)	-8(1)
C(19)	29(1)	26(1)	20(1)	-12(1)	0(1)	-6(1)
C(20)	31(1)	26(1)	26(1)	-13(1)	-4(1)	-6(1)
C(21)	46(1)	29(1)	25(1)	-10(1)	-11(1)	-9(1)
C(22)	57(1)	37(1)	21(1)	-16(1)	0(1)	-14(1)
C(23)	45(1)	32(1)	28(1)	-19(1)	5(1)	-5(1)
C(24)	34(1)	25(1)	23(1)	-12(1)	-1(1)	-3(1)
Cu(1)	26(1)	25(1)	19(1)	-11(1)	0(1)	-8(1)
Cu(2)	28(1)	29(1)	22(1)	-12(1)	3(1)	-11(1)
F(1)	45(1)	41(1)	38(1)	-12(1)	-18(1)	-4(1)
F(2)	60(1)	26(1)	52(1)	-13(1)	2(1)	-5(1)
F(3)	56(1)	53(1)	76(1)	-48(1)	12(1)	-28(1)
F(4)	54(1)	76(1)	54(1)	-41(1)	-13(1)	-23(1)
F(5)	48(1)	41(1)	38(1)	-15(1)	-20(1)	-2(1)
F(6)	34(1)	39(1)	46(1)	-22(1)	-8(1)	4(1)
F(7)	67(1)	44(1)	38(1)	-10(1)	-28(1)	-5(1)
F(8)	93(1)	64(1)	24(1)	-25(1)	0(1)	-19(1)
F(9)	69(1)	51(1)	47(1)	-33(1)	9(1)	7(1)
F(10)	44(1)	35(1)	37(1)	-13(1)	-10(1)	8(1)

O(1)	25(1)	26(1)	36(1)	-8(1)	-4(1)	-5(1)
S(1)	19(1)	27(1)	27(1)	-12(1)	-2(1)	-3(1)

Xray structure of $[Cu(C_6F_5)]_4(Tol_2SO)_2$ **7**

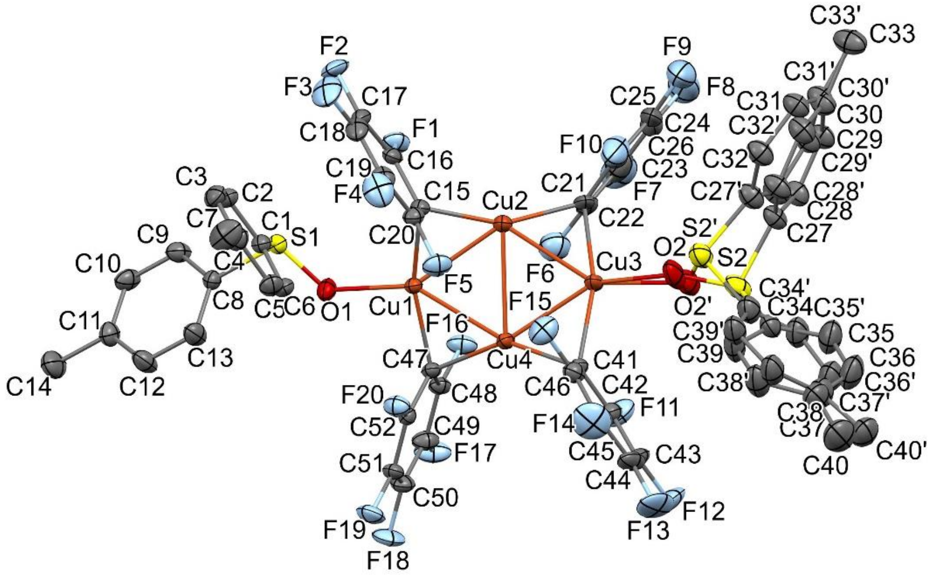

Figure S2 : Asymmetric Unit

Table S1. Crystal data and structure refinement for **7**.

Identification code	**7**
Empirical formula	C52 H28 Cu4 F20 O2 S2
Formula weight	1383.06
Temperature	193(2) K
Wavelength	0.71073 A
Crystal system, space group	Triclinic, P -1
Unit cell dimensions	a = 10.322(4) A alpha = 82.900(8) deg.
	b = 16.367(5) A beta = 73.207(12) deg.
	c = 16.807(4) A gamma = 73.320(13) deg.
Volume	2601.4(14) A^3
Z, Calculated density	2, 1.766 Mg/m^3
Absorption coefficient	1.806 mm^-1
F(000)	1368
Crystal size	0.160 x 0.080 x 0.060 mm
Theta range for data collection	2.760 to 29.334 deg.
Limiting indices	-14<=h<=14, -22<=k<=22, -23<=l<=23
Reflections collected / unique	86679 / 14193 [R(int) = 0.0980]
Completeness to theta = 25.242	99.9 %
Refinement method	Full-matrix least-squares on F^2
Data / restraints / parameters	14193 / 618 / 869
Goodness-of-fit on F^2	1.020
Final R indices [I>2sigma(I)]	R1 = 0.0566, wR2 = 0.1241
R indices (all data)	R1 = 0.1177, wR2 = 0.1496
Largest diff. peak and hole	1.107 and -0.651 e.A^-3

Table S2. Atomic coordinates (x 10^4) and equivalent isotropic displacement parameters (A^2 x 10^3) for **7**.

U(eq) is defined as one third of the trace of the orthogonalized Uij tensor.

	x	y	z	U(eq)
C(1)	1568(4)	5300(3)	2312(3)	36(1)
C(2)	498(5)	5579(3)	1920(3)	42(1)
C(3)	810(5)	5818(3)	1083(3)	48(1)
C(4)	2184(5)	5769(3)	618(3)	48(1)
C(5)	3248(5)	5494(3)	1030(3)	48(1)
C(6)	2945(5)	5252(3)	1873(3)	40(1)
C(7)	2514(7)	5994(5)	-300(4)	77(2)
C(8)	662(4)	5988(3)	3831(3)	37(1)
C(9)	-646(5)	6253(3)	4393(3)	48(1)
C(10)	-1020(5)	7043(4)	4732(3)	53(1)
C(11)	-133(5)	7570(3)	4531(3)	50(1)
C(12)	1182(6)	7282(3)	3973(4)	59(2)
C(13)	1565(5)	6503(3)	3623(4)	57(1)
C(14)	-534(7)	8441(4)	4888(4)	77(2)
C(15)	2969(4)	2957(3)	2415(3)	33(1)
C(16)	1517(4)	3062(3)	2621(3)	40(1)
C(17)	725(5)	3361(3)	2055(4)	52(1)
C(18)	1376(6)	3557(3)	1246(4)	57(2)
C(19)	2775(6)	3461(3)	1006(3)	50(1)
C(20)	3543(4)	3175(3)	1587(3)	37(1)
C(21)	4615(5)	637(3)	3177(4)	53(1)

C(22)	4150(5)	515(4)	4100(4)	58(2)
C(23)	3670(7)	-140(5)	4453(5)	81(2)
C(24)	3566(7)	-743(4)	4032(7)	89(3)
C(25)	3938(7)	-692(4)	3175(7)	82(2)
C(26)	4467(6)	32(4)	2766(6)	78(2)
C(41)	8020(4)	1574(3)	2473(3)	35(1)
C(42)	9031(5)	1310(3)	2916(3)	46(1)
C(43)	10440(5)	1209(4)	2568(4)	62(2)
C(44)	10916(5)	1391(4)	1748(5)	71(2)
C(45)	9965(6)	1676(4)	1268(4)	60(2)
C(46)	8561(5)	1750(3)	1642(3)	44(1)
C(47)	5384(4)	3466(3)	3678(3)	33(1)
C(48)	5267(4)	3344(3)	4523(3)	36(1)
C(49)	5829(5)	3758(3)	4956(3)	46(1)
C(50)	6581(5)	4317(3)	4527(3)	46(1)
C(51)	6736(5)	4472(3)	3689(3)	39(1)
C(52)	6142(4)	4052(3)	3286(3)	32(1)
Cu(1)	3853(1)	3348(1)	3204(1)	30(1)
Cu(2)	4120(1)	1874(1)	2841(1)	36(1)
Cu(4)	6207(1)	2380(1)	3054(1)	33(1)
F(1)	839(3)	2879(2)	3411(2)	56(1)
F(2)	-675(3)	3453(2)	2293(3)	81(1)
F(3)	610(4)	3837(2)	693(3)	94(1)
F(4)	3410(4)	3661(2)	217(2)	80(1)
F(5)	4933(3)	3116(2)	1316(2)	56(1)
F(6)	4199(4)	1110(3)	4563(2)	82(1)
F(7)	3212(5)	-204(3)	5311(3)	116(2)
F(8)	3081(5)	-1400(3)	4393(4)	131(2)
F(9)	3810(5)	-1264(3)	2732(4)	132(2)
F(10)	4828(4)	88(3)	1931(3)	99(1)
F(11)	8645(3)	1122(2)	3740(2)	76(1)

F(12)	11382(4)	941(3)	3023(3)	102(2)
F(13)	12282(3)	1315(3)	1372(3)	110(2)
F(14)	10414(4)	1868(3)	449(2)	106(2)
F(15)	7672(3)	2006(2)	1148(2)	67(1)
F(16)	4526(3)	2797(2)	4981(2)	53(1)
F(17)	5636(3)	3631(2)	5783(2)	68(1)
F(18)	7144(4)	4730(2)	4927(2)	69(1)
F(19)	7482(3)	5025(2)	3272(2)	61(1)
F(20)	6316(3)	4230(2)	2459(2)	49(1)
O(1)	2536(3)	4507(2)	3579(2)	45(1)
S(1)	1141(1)	4971(1)	3394(1)	37(1)
Cu(3)	6505(1)	932(1)	2736(1)	39(1)
S(2)	8690(2)	-969(1)	2443(1)	61(1)
O(2)	7785(5)	-133(3)	2141(3)	72(1)
C(27)	8017(6)	-1791(3)	2253(3)	47(1)
C(28)	7463(6)	-2269(4)	2924(4)	51(1)
C(29)	6817(7)	-2873(4)	2798(4)	55(1)
C(30)	6698(7)	-2974(4)	2026(4)	51(1)
C(31)	7298(7)	-2487(4)	1362(4)	58(1)
C(32)	7947(7)	-1894(4)	1471(4)	55(1)
C(33)	5982(9)	-3617(5)	1893(4)	70(2)
C(34)	10333(6)	-1141(4)	1651(4)	57(1)
C(35)	11359(8)	-1896(5)	1618(5)	84(2)
C(36)	12653(8)	-1956(5)	1034(6)	91(2)
C(37)	12941(7)	-1302(5)	504(5)	77(2)
C(38)	11867(7)	-584(4)	508(5)	84(2)
C(39)	10561(7)	-502(4)	1089(5)	78(2)
C(40)	14365(8)	-1364(6)	-131(6)	108(3)
S(2')	8014(8)	-552(5)	1783(6)	57(2)
O(2')	7793(17)	-339(10)	2687(7)	60(3)
C(27')	7542(16)	-1528(7)	1856(7)	55(2)

C(28')	7340(30)	-1964(10)	2613(8)	53(3)
C(29')	6750(30)	-2657(11)	2720(8)	54(3)
C(30')	6520(20)	-2960(9)	2063(9)	55(3)
C(31')	6800(30)	-2524(11)	1296(9)	55(3)
C(32')	7260(30)	-1794(10)	1197(8)	57(3)
C(33')	5830(30)	-3687(14)	2185(15)	58(6)
C(34')	9893(9)	-905(10)	1393(8)	67(3)
C(35')	10703(12)	-1495(19)	1830(14)	74(3)
C(36')	12159(12)	-1700(20)	1509(13)	78(3)
C(37')	12798(11)	-1411(17)	754(12)	82(3)
C(38')	11967(12)	-820(20)	336(13)	77(3)
C(39')	10517(12)	-539(16)	679(13)	70(3)
C(40')	14358(15)	-1770(30)	330(20)	86(7)

Table S3. Bond lengths [A] and angles [deg] for **7**.

C(1)-C(6)	1.384(6)
C(1)-C(2)	1.385(6)
C(1)-S(1)	1.796(5)
C(2)-C(3)	1.382(7)
C(2)-H(2)	0.9500
C(3)-C(4)	1.393(7)
C(3)-H(3)	0.9500
C(4)-C(5)	1.402(7)
C(4)-C(7)	1.504(7)
C(5)-C(6)	1.392(7)
C(5)-H(5)	0.9500
C(6)-H(6)	0.9500
C(7)-H(7A)	0.9800
C(7)-H(7B)	0.9800
C(7)-H(7C)	0.9800
C(8)-C(13)	1.375(7)
C(8)-C(9)	1.390(6)
C(8)-S(1)	1.783(4)
C(9)-C(10)	1.385(7)
C(9)-H(9)	0.9500
C(10)-C(11)	1.378(7)
C(10)-H(10)	0.9500
C(11)-C(12)	1.397(7)
C(11)-C(14)	1.514(7)
C(12)-C(13)	1.374(7)
C(12)-H(12)	0.9500
C(13)-H(13)	0.9500
C(14)-H(14A)	0.9800

C(14)-H(14B)	0.9800
C(14)-H(14C)	0.9800
C(15)-C(20)	1.392(6)
C(15)-C(16)	1.401(6)
C(15)-Cu(2)	2.008(4)
C(15)-Cu(1)	2.059(4)
C(16)-F(1)	1.352(5)
C(16)-C(17)	1.384(7)
C(17)-F(2)	1.352(5)
C(17)-C(18)	1.376(8)
C(18)-F(3)	1.345(6)
C(18)-C(19)	1.350(8)
C(19)-F(4)	1.345(6)
C(19)-C(20)	1.387(6)
C(20)-F(5)	1.353(5)
C(21)-C(26)	1.341(8)
C(21)-C(22)	1.492(8)
C(21)-Cu(2)	1.993(5)
C(21)-Cu(3)	2.050(5)
C(22)-C(23)	1.313(9)
C(22)-F(6)	1.339(7)
C(23)-C(24)	1.328(10)
C(23)-F(7)	1.383(8)
C(24)-F(8)	1.321(8)
C(24)-C(25)	1.379(11)
C(25)-F(9)	1.322(8)
C(25)-C(26)	1.458(10)
C(26)-F(10)	1.343(9)
C(41)-C(46)	1.373(6)
C(41)-C(42)	1.393(6)
C(41)-Cu(4)	2.010(4)

C(41)-Cu(3)	2.044(4)
C(42)-F(11)	1.349(6)
C(42)-C(43)	1.371(7)
C(43)-C(44)	1.350(9)
C(43)-F(12)	1.350(6)
C(44)-F(13)	1.345(6)
C(44)-C(45)	1.390(9)
C(45)-F(14)	1.348(7)
C(45)-C(46)	1.379(7)
C(46)-F(15)	1.357(6)
C(47)-C(48)	1.385(6)
C(47)-C(52)	1.394(6)
C(47)-Cu(4)	2.015(4)
C(47)-Cu(1)	2.027(4)
C(48)-F(16)	1.364(5)
C(48)-C(49)	1.384(6)
C(49)-F(17)	1.344(5)
C(49)-C(50)	1.372(7)
C(50)-F(18)	1.346(5)
C(50)-C(51)	1.369(7)
C(51)-F(19)	1.354(5)
C(51)-C(52)	1.382(6)
C(52)-F(20)	1.354(5)
Cu(1)-O(1)	2.040(3)
Cu(1)-Cu(4)	2.4525(11)
Cu(1)-Cu(2)	2.4806(10)
Cu(2)-Cu(3)	2.4719(12)
Cu(2)-Cu(4)	2.6455(11)
Cu(4)-Cu(3)	2.4076(10)
O(1)-S(1)	1.523(3)
Cu(3)-O(2)	2.033(4)

Cu(3)-O(2')	2.119(17)
S(2)-O(2)	1.539(5)
S(2)-C(27)	1.780(5)
S(2)-C(34)	1.800(6)
C(27)-C(32)	1.370(8)
C(27)-C(28)	1.370(8)
C(28)-C(29)	1.407(8)
C(28)-H(28)	0.9500
C(29)-C(30)	1.372(7)
C(29)-H(29)	0.9500
C(30)-C(31)	1.389(7)
C(30)-C(33)	1.518(8)
C(31)-C(32)	1.383(8)
C(31)-H(31)	0.9500
C(32)-H(32)	0.9500
C(33)-H(33A)	0.9800
C(33)-H(33B)	0.9800
C(33)-H(33C)	0.9800
C(34)-C(39)	1.346(9)
C(34)-C(35)	1.372(9)
C(35)-C(36)	1.396(10)
C(35)-H(35)	0.9500
C(36)-C(37)	1.350(10)
C(36)-H(36)	0.9500
C(37)-C(38)	1.364(9)
C(37)-C(40)	1.531(9)
C(38)-C(39)	1.399(8)
C(38)-H(38)	0.9500
C(39)-H(39)	0.9500
C(40)-H(40A)	0.9800
C(40)-H(40B)	0.9800

C(40)-H(40C)	0.9800
S(2')-O(2')	1.539(5)
S(2')-C(27')	1.780(6)
S(2')-C(34')	1.800(6)
C(27')-C(32')	1.370(8)
C(27')-C(28')	1.370(8)
C(28')-C(29')	1.407(8)
C(28')-H(28')	0.9500
C(29')-C(30')	1.372(7)
C(29')-H(29')	0.9500
C(30')-C(31')	1.389(7)
C(30')-C(33')	1.518(8)
C(31')-C(32')	1.383(8)
C(31')-H(31')	0.9500
C(32')-H(32')	0.9500
C(33')-H(33D)	0.9800
C(33')-H(33E)	0.9800
C(33')-H(33F)	0.9800
C(34')-C(39')	1.346(9)
C(34')-C(35')	1.372(9)
C(35')-C(36')	1.396(10)
C(35')-H(35')	0.9500
C(36')-C(37')	1.350(10)
C(36')-H(36')	0.9500
C(37')-C(38')	1.364(9)
C(37')-C(40')	1.531(10)
C(38')-C(39')	1.399(9)
C(38')-H(38')	0.9500
C(39')-H(39')	0.9500
C(40')-H(40D)	0.9800
C(40')-H(40E)	0.9800

C(40')-H(40F)	0.9800

C(6)-C(1)-C(2)	120.7(4)
C(6)-C(1)-S(1)	120.6(3)
C(2)-C(1)-S(1)	118.7(3)
C(3)-C(2)-C(1)	119.6(4)
C(3)-C(2)-H(2)	120.2
C(1)-C(2)-H(2)	120.2
C(2)-C(3)-C(4)	121.4(4)
C(2)-C(3)-H(3)	119.3
C(4)-C(3)-H(3)	119.3
C(3)-C(4)-C(5)	118.0(5)
C(3)-C(4)-C(7)	120.8(5)
C(5)-C(4)-C(7)	121.2(5)
C(6)-C(5)-C(4)	121.0(4)
C(6)-C(5)-H(5)	119.5
C(4)-C(5)-H(5)	119.5
C(1)-C(6)-C(5)	119.3(4)
C(1)-C(6)-H(6)	120.4
C(5)-C(6)-H(6)	120.4
C(4)-C(7)-H(7A)	109.5
C(4)-C(7)-H(7B)	109.5
H(7A)-C(7)-H(7B)	109.5
C(4)-C(7)-H(7C)	109.5
H(7A)-C(7)-H(7C)	109.5
H(7B)-C(7)-H(7C)	109.5
C(13)-C(8)-C(9)	120.4(4)
C(13)-C(8)-S(1)	121.0(3)
C(9)-C(8)-S(1)	118.6(4)
C(10)-C(9)-C(8)	118.7(5)
C(10)-C(9)-H(9)	120.7

C(8)-C(9)-H(9)	120.7
C(11)-C(10)-C(9)	122.0(4)
C(11)-C(10)-H(10)	119.0
C(9)-C(10)-H(10)	119.0
C(10)-C(11)-C(12)	118.0(4)
C(10)-C(11)-C(14)	122.5(5)
C(12)-C(11)-C(14)	119.4(5)
C(13)-C(12)-C(11)	120.9(5)
C(13)-C(12)-H(12)	119.6
C(11)-C(12)-H(12)	119.6
C(12)-C(13)-C(8)	120.1(4)
C(12)-C(13)-H(13)	119.9
C(8)-C(13)-H(13)	119.9
C(11)-C(14)-H(14A)	109.5
C(11)-C(14)-H(14B)	109.5
H(14A)-C(14)-H(14B)	109.5
C(11)-C(14)-H(14C)	109.5
H(14A)-C(14)-H(14C)	109.5
H(14B)-C(14)-H(14C)	109.5
C(20)-C(15)-C(16)	113.7(4)
C(20)-C(15)-Cu(2)	113.7(3)
C(16)-C(15)-Cu(2)	116.8(3)
C(20)-C(15)-Cu(1)	111.8(3)
C(16)-C(15)-Cu(1)	120.4(3)
Cu(2)-C(15)-Cu(1)	75.17(14)
F(1)-C(16)-C(17)	117.7(4)
F(1)-C(16)-C(15)	119.1(4)
C(17)-C(16)-C(15)	123.2(5)
F(2)-C(17)-C(18)	120.1(5)
F(2)-C(17)-C(16)	120.4(5)
C(18)-C(17)-C(16)	119.5(4)

F(3)-C(18)-C(19)	120.2(6)
F(3)-C(18)-C(17)	119.6(5)
C(19)-C(18)-C(17)	120.2(5)
F(4)-C(19)-C(18)	120.3(5)
F(4)-C(19)-C(20)	120.4(5)
C(18)-C(19)-C(20)	119.3(5)
F(5)-C(20)-C(19)	116.5(4)
F(5)-C(20)-C(15)	119.3(4)
C(19)-C(20)-C(15)	124.2(4)
C(26)-C(21)-C(22)	113.6(6)
C(26)-C(21)-Cu(2)	123.1(5)
C(22)-C(21)-Cu(2)	110.3(4)
C(26)-C(21)-Cu(3)	114.7(5)
C(22)-C(21)-Cu(3)	114.5(3)
Cu(2)-C(21)-Cu(3)	75.37(17)
C(23)-C(22)-F(6)	120.6(7)
C(23)-C(22)-C(21)	121.4(6)
F(6)-C(22)-C(21)	117.9(4)
C(22)-C(23)-C(24)	123.8(8)
C(22)-C(23)-F(7)	118.8(8)
C(24)-C(23)-F(7)	117.3(8)
F(8)-C(24)-C(23)	123.4(10)
F(8)-C(24)-C(25)	116.5(7)
C(23)-C(24)-C(25)	120.1(7)
F(9)-C(25)-C(24)	122.1(7)
F(9)-C(25)-C(26)	120.7(9)
C(24)-C(25)-C(26)	117.3(7)
C(21)-C(26)-F(10)	118.6(6)
C(21)-C(26)-C(25)	123.7(8)
F(10)-C(26)-C(25)	117.7(6)
C(46)-C(41)-C(42)	113.6(4)

C(46)-C(41)-Cu(4)	117.0(3)
C(42)-C(41)-Cu(4)	115.6(3)
C(46)-C(41)-Cu(3)	115.1(3)
C(42)-C(41)-Cu(3)	117.0(4)
Cu(4)-C(41)-Cu(3)	72.87(14)
F(11)-C(42)-C(43)	115.8(5)
F(11)-C(42)-C(41)	119.9(4)
C(43)-C(42)-C(41)	124.3(5)
C(44)-C(43)-F(12)	118.2(5)
C(44)-C(43)-C(42)	119.7(5)
F(12)-C(43)-C(42)	122.1(6)
F(13)-C(44)-C(43)	122.5(6)
F(13)-C(44)-C(45)	118.2(7)
C(43)-C(44)-C(45)	119.3(5)
F(14)-C(45)-C(46)	120.7(6)
F(14)-C(45)-C(44)	120.4(5)
C(46)-C(45)-C(44)	118.9(5)
F(15)-C(46)-C(41)	118.7(4)
F(15)-C(46)-C(45)	117.1(5)
C(41)-C(46)-C(45)	124.2(5)
C(48)-C(47)-C(52)	113.6(4)
C(48)-C(47)-Cu(4)	113.8(3)
C(52)-C(47)-Cu(4)	108.5(3)
C(48)-C(47)-Cu(1)	120.9(3)
C(52)-C(47)-Cu(1)	118.1(3)
Cu(4)-C(47)-Cu(1)	74.71(14)
F(16)-C(48)-C(49)	116.2(4)
F(16)-C(48)-C(47)	119.3(4)
C(49)-C(48)-C(47)	124.5(4)
F(17)-C(49)-C(50)	119.8(4)
F(17)-C(49)-C(48)	121.4(4)

C(50)-C(49)-C(48)	118.8(4)
F(18)-C(50)-C(51)	119.6(4)
F(18)-C(50)-C(49)	120.4(4)
C(51)-C(50)-C(49)	120.0(4)
F(19)-C(51)-C(50)	119.6(4)
F(19)-C(51)-C(52)	121.3(4)
C(50)-C(51)-C(52)	119.1(4)
F(20)-C(52)-C(51)	117.0(4)
F(20)-C(52)-C(47)	118.9(4)
C(51)-C(52)-C(47)	124.0(4)
C(47)-Cu(1)-O(1)	94.39(15)
C(47)-Cu(1)-C(15)	157.99(17)
O(1)-Cu(1)-C(15)	104.69(15)
C(47)-Cu(1)-Cu(4)	52.41(12)
O(1)-Cu(1)-Cu(4)	146.36(9)
C(15)-Cu(1)-Cu(4)	107.09(12)
C(47)-Cu(1)-Cu(2)	114.19(12)
O(1)-Cu(1)-Cu(2)	147.87(10)
C(15)-Cu(1)-Cu(2)	51.48(12)
Cu(4)-Cu(1)-Cu(2)	64.86(3)
C(21)-Cu(2)-C(15)	155.71(17)
C(21)-Cu(2)-Cu(3)	53.37(13)
C(15)-Cu(2)-Cu(3)	145.80(12)
C(21)-Cu(2)-Cu(1)	146.17(17)
C(15)-Cu(2)-Cu(1)	53.34(11)
Cu(3)-Cu(2)-Cu(1)	113.05(3)
C(21)-Cu(2)-Cu(4)	102.38(13)
C(15)-Cu(2)-Cu(4)	101.89(12)
Cu(3)-Cu(2)-Cu(4)	56.01(3)
Cu(1)-Cu(2)-Cu(4)	57.06(2)
C(41)-Cu(4)-C(47)	143.60(16)

C(41)-Cu(4)-Cu(3)	54.22(12)
C(47)-Cu(4)-Cu(3)	158.02(12)
C(41)-Cu(4)-Cu(1)	156.59(13)
C(47)-Cu(4)-Cu(1)	52.88(11)
Cu(3)-Cu(4)-Cu(1)	116.41(3)
C(41)-Cu(4)-Cu(2)	108.07(12)
C(47)-Cu(4)-Cu(2)	108.19(11)
Cu(3)-Cu(4)-Cu(2)	58.35(2)
Cu(1)-Cu(4)-Cu(2)	58.09(3)
S(1)-O(1)-Cu(1)	129.22(18)
O(1)-S(1)-C(8)	104.82(19)
O(1)-S(1)-C(1)	105.7(2)
C(8)-S(1)-C(1)	98.9(2)
O(2)-Cu(3)-C(41)	95.02(18)
O(2)-Cu(3)-C(21)	103.0(2)
C(41)-Cu(3)-C(21)	161.97(19)
C(41)-Cu(3)-O(2')	99.4(4)
C(21)-Cu(3)-O(2')	96.9(4)
O(2)-Cu(3)-Cu(4)	147.66(13)
C(41)-Cu(3)-Cu(4)	52.91(12)
C(21)-Cu(3)-Cu(4)	109.09(15)
O(2')-Cu(3)-Cu(4)	146.3(4)
O(2)-Cu(3)-Cu(2)	138.28(15)
C(41)-Cu(3)-Cu(2)	113.61(12)
C(21)-Cu(3)-Cu(2)	51.26(14)
O(2')-Cu(3)-Cu(2)	146.6(4)
Cu(4)-Cu(3)-Cu(2)	65.65(3)
O(2)-S(2)-C(27)	105.3(3)
O(2)-S(2)-C(34)	103.6(3)
C(27)-S(2)-C(34)	101.3(3)
S(2)-O(2)-Cu(3)	132.3(3)

C(32)-C(27)-C(28)	121.1(5)
C(32)-C(27)-S(2)	120.9(4)
C(28)-C(27)-S(2)	117.8(4)
C(27)-C(28)-C(29)	118.8(5)
C(27)-C(28)-H(28)	120.6
C(29)-C(28)-H(28)	120.6
C(30)-C(29)-C(28)	121.5(5)
C(30)-C(29)-H(29)	119.3
C(28)-C(29)-H(29)	119.3
C(29)-C(30)-C(31)	117.7(5)
C(29)-C(30)-C(33)	121.4(5)
C(31)-C(30)-C(33)	120.8(5)
C(32)-C(31)-C(30)	121.7(6)
C(32)-C(31)-H(31)	119.1
C(30)-C(31)-H(31)	119.1
C(27)-C(32)-C(31)	119.2(5)
C(27)-C(32)-H(32)	120.4
C(31)-C(32)-H(32)	120.4
C(30)-C(33)-H(33A)	109.5
C(30)-C(33)-H(33B)	109.5
H(33A)-C(33)-H(33B)	109.5
C(30)-C(33)-H(33C)	109.5
H(33A)-C(33)-H(33C)	109.5
H(33B)-C(33)-H(33C)	109.5
C(39)-C(34)-C(35)	119.6(6)
C(39)-C(34)-S(2)	118.5(5)
C(35)-C(34)-S(2)	121.8(5)
C(34)-C(35)-C(36)	118.5(7)
C(34)-C(35)-H(35)	120.7
C(36)-C(35)-H(35)	120.7
C(37)-C(36)-C(35)	122.9(7)

C(37)-C(36)-H(36)	118.6
C(35)-C(36)-H(36)	118.6
C(36)-C(37)-C(38)	117.1(7)
C(36)-C(37)-C(40)	122.9(7)
C(38)-C(37)-C(40)	119.8(8)
C(37)-C(38)-C(39)	121.2(7)
C(37)-C(38)-H(38)	119.4
C(39)-C(38)-H(38)	119.4
C(34)-C(39)-C(38)	120.3(6)
C(34)-C(39)-H(39)	119.9
C(38)-C(39)-H(39)	119.9
C(37)-C(40)-H(40A)	109.5
C(37)-C(40)-H(40B)	109.5
H(40A)-C(40)-H(40B)	109.5
C(37)-C(40)-H(40C)	109.5
H(40A)-C(40)-H(40C)	109.5
H(40B)-C(40)-H(40C)	109.5
O(2')-S(2')-C(27')	105.3(3)
O(2')-S(2')-C(34')	103.6(3)
C(27')-S(2')-C(34')	101.3(3)
S(2')-O(2')-Cu(3)	102.2(8)
C(32')-C(27')-C(28')	121.0(5)
C(32')-C(27')-S(2')	120.9(5)
C(28')-C(27')-S(2')	117.8(5)
C(27')-C(28')-C(29')	118.7(6)
C(27')-C(28')-H(28')	120.7
C(29')-C(28')-H(28')	120.7
C(30')-C(29')-C(28')	121.4(5)
C(30')-C(29')-H(29')	119.3
C(28')-C(29')-H(29')	119.3
C(29')-C(30')-C(31')	117.6(5)

C(29')-C(30')-C(33')	121.3(5)
C(31')-C(30')-C(33')	120.7(6)
C(32')-C(31')-C(30')	121.7(6)
C(32')-C(31')-H(31')	119.2
C(30')-C(31')-H(31')	119.2
C(27')-C(32')-C(31')	119.2(6)
C(27')-C(32')-H(32')	120.4
C(31')-C(32')-H(32')	120.4
C(30')-C(33')-H(33D)	109.5
C(30')-C(33')-H(33E)	109.5
H(33D)-C(33')-H(33E)	109.5
C(30')-C(33')-H(33F)	109.5
H(33D)-C(33')-H(33F)	109.5
H(33E)-C(33')-H(33F)	109.5
C(39')-C(34')-C(35')	119.6(6)
C(39')-C(34')-S(2')	118.5(5)
C(35')-C(34')-S(2')	121.8(6)
C(34')-C(35')-C(36')	118.5(7)
C(34')-C(35')-H(35')	120.8
C(36')-C(35')-H(35')	120.8
C(37')-C(36')-C(35')	122.8(7)
C(37')-C(36')-H(36')	118.6
C(35')-C(36')-H(36')	118.6
C(36')-C(37')-C(38')	117.1(7)
C(36')-C(37')-C(40')	122.9(7)
C(38')-C(37')-C(40')	119.8(8)
C(37')-C(38')-C(39')	121.2(7)
C(37')-C(38')-H(38')	119.4
C(39')-C(38')-H(38')	119.4
C(34')-C(39')-C(38')	120.2(6)
C(34')-C(39')-H(39')	119.9

C(38')-C(39')-H(39')	119.9
C(37')-C(40')-H(40D)	109.5
C(37')-C(40')-H(40E)	109.5
H(40D)-C(40')-H(40E)	109.5
C(37')-C(40')-H(40F)	109.5
H(40D)-C(40')-H(40F)	109.5
H(40E)-C(40')-H(40F)	109.5

Symmetry transformations used to generate equivalent atoms:

Table S4. Anisotropic displacement parameters (A^2 x 10^3) for **7**.

The anisotropic displacement factor exponent takes the form:

-2 pi^2 [h^2 a*^2 U11 + ... + 2 h k a* b* U12]

	U11	U22	U33	U23	U13	U12
C(1)	31(2)	35(2)	43(3)	-5(2)	-12(2)	-8(2)
C(2)	30(2)	45(3)	51(3)	-2(2)	-12(2)	-10(2)
C(3)	40(3)	48(3)	59(3)	8(2)	-23(2)	-8(2)
C(4)	43(3)	49(3)	50(3)	10(2)	-13(2)	-13(2)
C(5)	33(2)	51(3)	56(3)	-1(2)	-3(2)	-13(2)
C(6)	30(2)	40(3)	53(3)	-3(2)	-16(2)	-9(2)
C(7)	64(4)	104(5)	58(4)	30(4)	-13(3)	-27(4)
C(8)	30(2)	34(2)	46(3)	-10(2)	-11(2)	-4(2)
C(9)	39(3)	59(3)	50(3)	-17(2)	-5(2)	-18(2)
C(10)	31(2)	68(4)	56(3)	-27(3)	0(2)	-10(2)
C(11)	46(3)	44(3)	55(3)	-18(2)	-9(2)	-3(2)
C(12)	55(3)	49(3)	73(4)	-19(3)	2(3)	-26(3)
C(13)	36(3)	51(3)	74(4)	-24(3)	7(2)	-12(2)
C(14)	65(4)	61(4)	103(5)	-41(4)	-12(4)	-8(3)
C(15)	29(2)	32(2)	44(3)	-6(2)	-13(2)	-11(2)
C(16)	31(2)	36(2)	57(3)	-17(2)	-9(2)	-12(2)
C(17)	31(2)	43(3)	89(4)	-22(3)	-28(3)	-2(2)
C(18)	67(4)	50(3)	64(4)	-13(3)	-44(3)	-3(3)
C(19)	62(3)	49(3)	43(3)	-6(2)	-23(2)	-12(3)
C(20)	33(2)	43(3)	40(3)	-8(2)	-11(2)	-11(2)
C(21)	29(2)	37(3)	103(5)	2(3)	-29(3)	-15(2)
C(22)	27(2)	45(3)	101(5)	10(3)	-17(3)	-13(2)

C(23)	46(3)	86(5)	99(6)	19(4)	-19(4)	-8(3)
C(24)	58(4)	43(4)	178(9)	-3(5)	-53(5)	-11(3)
C(25)	47(3)	38(3)	175(8)	-14(4)	-45(5)	-12(3)
C(26)	46(3)	53(4)	149(7)	-25(4)	-45(4)	-7(3)
C(41)	22(2)	36(2)	48(3)	-9(2)	-6(2)	-9(2)
C(42)	34(2)	47(3)	58(3)	-14(2)	-14(2)	-8(2)
C(43)	27(2)	69(4)	93(5)	-27(3)	-21(3)	-4(2)
C(44)	24(2)	73(4)	113(5)	-46(4)	5(3)	-16(3)
C(45)	50(3)	65(4)	59(4)	-18(3)	14(3)	-28(3)
C(46)	40(3)	43(3)	50(3)	-10(2)	-5(2)	-15(2)
C(47)	23(2)	37(2)	42(2)	-7(2)	-7(2)	-11(2)
C(48)	27(2)	44(3)	39(2)	-8(2)	-3(2)	-14(2)
C(49)	40(3)	65(3)	38(3)	-14(2)	-6(2)	-21(2)
C(50)	41(3)	53(3)	54(3)	-16(2)	-17(2)	-18(2)
C(51)	33(2)	37(2)	53(3)	-6(2)	-11(2)	-18(2)
C(52)	25(2)	32(2)	38(2)	-6(2)	-10(2)	-5(2)
Cu(1)	24(1)	31(1)	38(1)	-7(1)	-10(1)	-8(1)
Cu(2)	29(1)	31(1)	52(1)	-4(1)	-13(1)	-10(1)
Cu(4)	23(1)	33(1)	45(1)	-11(1)	-7(1)	-7(1)
F(1)	37(2)	69(2)	58(2)	-11(2)	5(1)	-24(1)
F(2)	28(2)	88(3)	137(3)	-40(2)	-33(2)	-4(2)
F(3)	110(3)	92(3)	104(3)	-11(2)	-87(3)	-1(2)
F(4)	116(3)	82(3)	42(2)	-1(2)	-24(2)	-23(2)
F(5)	40(2)	78(2)	50(2)	-6(2)	1(1)	-25(2)
F(6)	69(2)	90(3)	86(3)	1(2)	-13(2)	-28(2)
F(7)	81(3)	126(4)	130(4)	53(3)	-24(3)	-40(3)
F(8)	92(3)	82(3)	221(6)	36(3)	-41(4)	-43(3)
F(9)	94(3)	86(3)	237(6)	-57(4)	-73(4)	-8(3)
F(10)	86(3)	92(3)	127(4)	-51(3)	-51(3)	5(2)
F(11)	59(2)	103(3)	66(2)	4(2)	-29(2)	-13(2)
F(12)	44(2)	128(4)	141(4)	-43(3)	-51(2)	9(2)

F(13)	24(2)	136(4)	158(4)	-63(3)	22(2)	-27(2)
F(14)	91(3)	134(4)	68(3)	-14(2)	35(2)	-46(3)
F(15)	60(2)	86(2)	50(2)	-1(2)	-15(2)	-12(2)
F(16)	52(2)	73(2)	44(2)	1(1)	-8(1)	-38(2)
F(17)	70(2)	113(3)	37(2)	-12(2)	-11(1)	-50(2)
F(18)	75(2)	81(2)	76(2)	-22(2)	-28(2)	-46(2)
F(19)	65(2)	56(2)	78(2)	7(2)	-24(2)	-44(2)
F(20)	54(2)	56(2)	45(2)	7(1)	-17(1)	-27(1)
O(1)	44(2)	41(2)	51(2)	-15(2)	-22(2)	4(2)
S(1)	34(1)	34(1)	43(1)	-7(1)	-12(1)	-7(1)
Cu(3)	25(1)	31(1)	63(1)	-10(1)	-6(1)	-10(1)
S(2)	63(1)	54(1)	65(1)	-23(1)	1(1)	-25(1)
O(2)	59(3)	41(2)	100(4)	-28(2)	11(2)	-9(2)
C(27)	54(3)	44(3)	43(3)	-8(2)	0(2)	-22(2)
C(28)	64(3)	49(3)	43(3)	2(2)	-13(2)	-21(3)
C(29)	64(3)	50(3)	51(3)	10(2)	-10(2)	-28(3)
C(30)	56(3)	49(3)	52(3)	2(2)	-14(2)	-20(2)
C(31)	73(3)	54(3)	46(3)	-4(2)	-9(3)	-24(3)
C(32)	73(3)	54(3)	43(3)	-2(2)	-7(2)	-32(3)
C(33)	95(5)	77(5)	57(4)	11(3)	-28(4)	-48(4)
C(34)	49(3)	40(3)	79(3)	-14(2)	-10(2)	-12(2)
C(35)	65(3)	62(3)	101(4)	6(3)	-13(3)	4(3)
C(36)	58(3)	75(4)	114(5)	-4(4)	-13(3)	11(3)
C(37)	49(3)	68(3)	105(4)	-15(3)	-8(3)	-10(3)
C(38)	62(3)	51(3)	114(4)	-4(3)	12(3)	-13(3)
C(39)	57(3)	40(3)	110(4)	-5(3)	9(3)	-3(3)
C(40)	56(4)	105(7)	142(7)	-29(5)	10(4)	-15(4)
S(2')	52(4)	48(4)	66(4)	-14(3)	-5(3)	-14(3)
O(2')	58(6)	47(6)	70(6)	-19(5)	-1(5)	-14(5)
C(27')	64(4)	50(4)	48(4)	-5(4)	-3(4)	-23(4)
C(28')	64(4)	51(4)	47(4)	-4(4)	-9(4)	-23(4)

C(29')	64(5)	51(5)	50(4)	3(4)	-11(4)	-24(4)
C(30')	65(5)	52(4)	50(4)	2(4)	-12(4)	-25(4)
C(31')	66(5)	54(5)	49(4)	-2(4)	-10(5)	-27(4)
C(32')	69(6)	54(5)	48(5)	-4(5)	-6(5)	-26(5)
C(33')	68(11)	61(10)	53(11)	3(9)	-17(10)	-32(8)
C(34')	55(4)	47(4)	87(4)	-11(4)	-2(4)	-10(4)
C(35')	57(4)	51(5)	95(5)	-9(5)	-3(5)	-3(5)
C(36')	56(4)	62(5)	101(5)	-8(4)	-9(4)	-3(4)
C(37')	54(4)	64(5)	111(5)	-9(5)	-2(4)	-6(5)
C(38')	55(5)	53(5)	106(5)	-8(5)	3(5)	-8(5)
C(39')	54(4)	44(5)	97(5)	-8(5)	1(5)	-9(5)
C(40')	48(7)	79(10)	116(11)	-8(10)	-7(8)	-8(9)

Xray structure of $[Cu(C_6F_5)]_4(Anthra_2SO)_2$ 8:

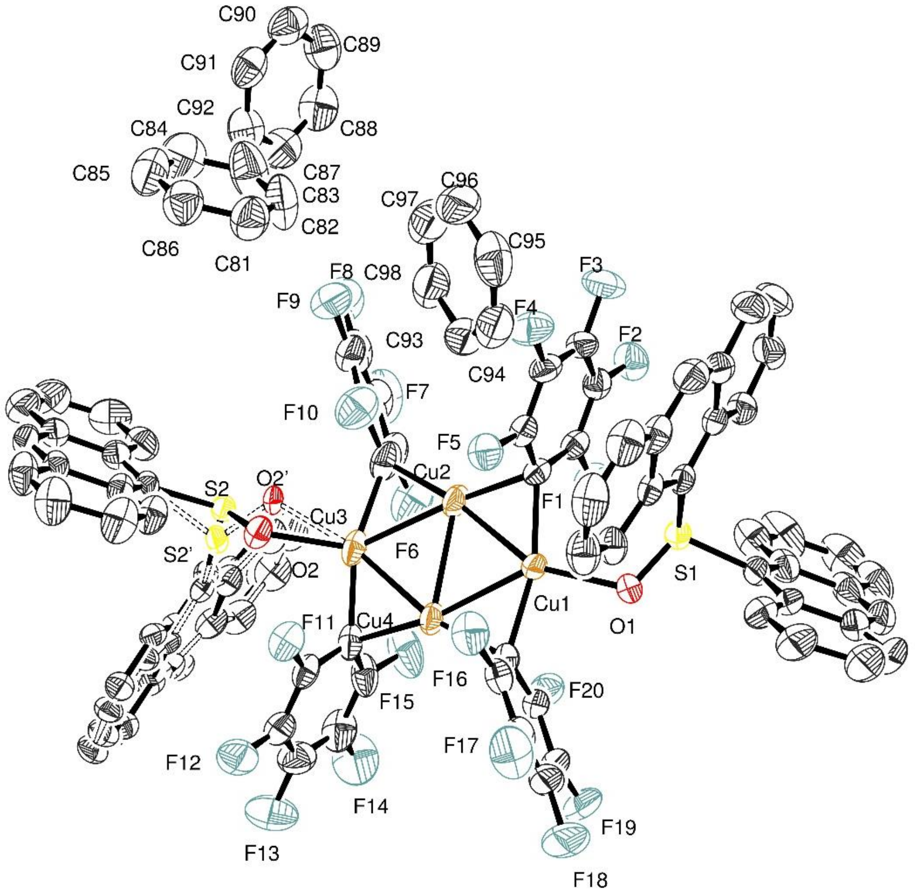

Figure S2 : Asymmetric Unit

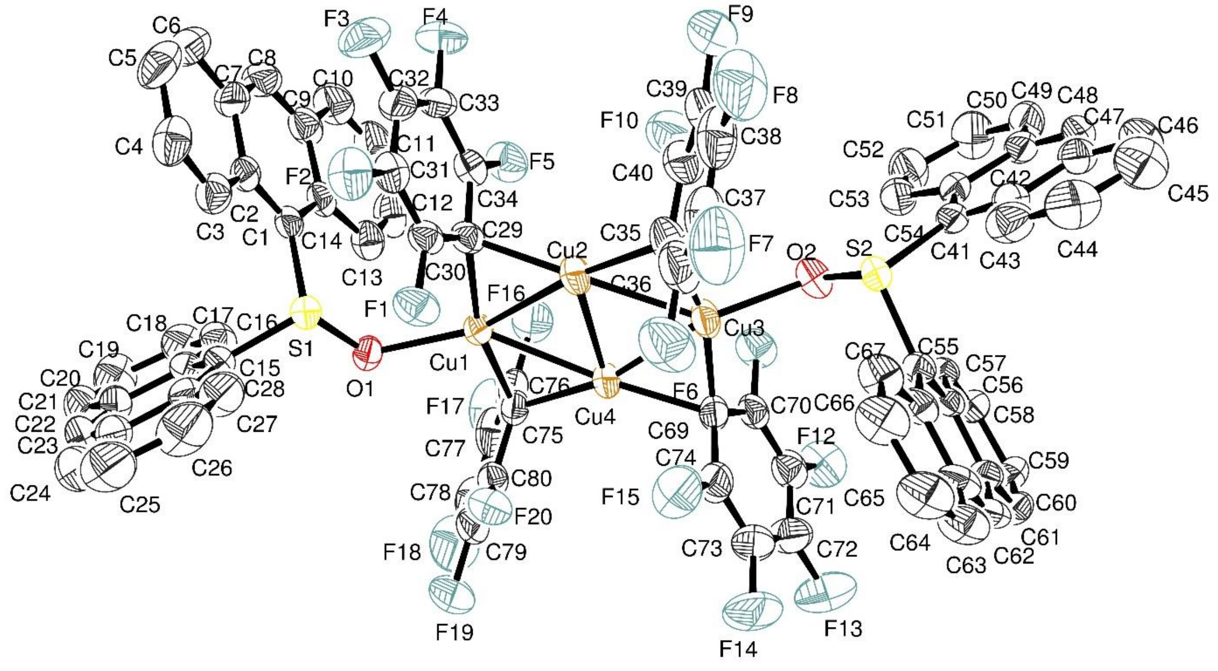

Figure S3 : Compound

Table S1. Crystal data and structure refinement for **8**.

Identification code	**8**
Empirical formula	C80 H36 Cu4 F20 O2 S2, 3(C6 H6)
Formula weight	1961.73
Temperature	193(2) K
Wavelength	1.54178 A
Crystal system, space group	Monoclinic, P 21/c
Unit cell dimensions	a = 16.0110(6) A alpha = 90 deg.
	b = 49.4068(18) A beta = 109.141(2) deg.
	c = 10.9222(4) A gamma = 90 deg.
Volume	8162.4(5) A^3
Z, Calculated density	4, 1.596 Mg/m^3
Absorption coefficient	2.511 mm^-1
F(000)	3944
Crystal size	0.200 x 0.180 x 0.040 mm
Theta range for data collection	3.055 to 68.390 deg.
Limiting indices	-19<=h<=19, -59<=k<=59, -13<=l<=13
Reflections collected / unique	123416 / 14964 [R(int) = 0.0491]
Completeness to theta = 67.679	99.9 %
Refinement method	Full-matrix least-squares on F^2
Data / restraints / parameters	14964 / 662 / 1281
Goodness-of-fit on F^2	1.204
Final R indices [I>2sigma(I)]	R1 = 0.0660, wR2 = 0.1406
R indices (all data)	R1 = 0.0685, wR2 = 0.1417
Largest diff. peak and hole	0.950 and -1.228 e.A^-3

Table S2. Atomic coordinates (x 10^4) and equivalent isotropic displacement parameters (A^2 x 10^3) for **8**.
U(eq) is defined as one third of the trace of the orthogonalized Uij tensor.

	x	y	z	U(eq)
C(1)	7587(3)	3189(1)	3710(4)	36(1)
C(2)	7975(3)	3065(1)	4934(4)	39(1)
C(3)	8539(3)	3202(1)	6054(5)	48(1)
C(4)	8863(4)	3075(1)	7214(5)	59(1)
C(5)	8664(4)	2800(1)	7357(6)	69(2)
C(6)	8148(4)	2662(1)	6324(6)	64(2)
C(7)	7779(3)	2788(1)	5090(5)	48(1)
C(8)	7223(3)	2648(1)	4037(6)	55(1)
C(9)	6812(3)	2769(1)	2839(5)	49(1)
C(10)	6229(4)	2619(1)	1774(7)	65(2)
C(11)	5826(4)	2733(1)	628(7)	70(2)
C(12)	5962(3)	3011(1)	447(6)	60(1)
C(13)	6508(3)	3164(1)	1424(4)	45(1)
C(14)	6978(3)	3051(1)	2653(4)	39(1)
C(15)	8944(3)	3546(1)	3626(5)	42(1)
C(16)	9198(3)	3407(1)	2677(5)	45(1)
C(17)	8613(3)	3264(1)	1628(5)	52(1)
C(18)	8904(4)	3124(1)	760(6)	68(2)
C(19)	9814(4)	3122(1)	900(6)	75(2)
C(20)	10391(4)	3266(1)	1841(6)	69(2)
C(21)	10117(3)	3413(1)	2771(6)	58(1)
C(22)	10701(3)	3561(1)	3763(6)	67(2)
C(23)	10450(3)	3705(1)	4654(6)	61(2)
C(24)	11061(4)	3862(1)	5674(8)	88(2)
C(25)	10819(5)	3999(1)	6563(8)	94(3)
C(26)	9919(5)	4000(1)	6492(7)	89(2)
C(27)	9299(4)	3856(1)	5554(6)	66(2)
C(28)	9537(3)	3702(1)	4612(5)	51(1)
C(29)	5956(3)	3632(1)	4221(4)	37(1)
C(30)	6574(3)	3680(1)	5440(4)	39(1)
C(31)	6773(3)	3500(1)	6451(4)	42(1)
C(32)	6350(3)	3254(1)	6268(4)	46(1)
C(33)	5749(3)	3192(1)	5081(5)	44(1)
C(34)	5559(3)	3379(1)	4099(4)	40(1)
C(35)	4104(3)	4048(1)	4582(5)	59(1)
C(36)	4335(4)	4239(1)	5543(7)	71(2)
C(37)	4106(4)	4223(2)	6681(6)	72(2)
C(38)	3658(4)	4005(2)	6869(6)	76(2)
C(39)	3437(4)	3802(1)	5968(7)	69(2)
C(40)	3625(4)	3829(1)	4848(5)	57(1)
C(42)	386(3)	4407(1)	3411(5)	46(1)
C(43)	824(4)	4572(1)	4522(5)	61(1)
C(44)	382(5)	4683(1)	5273(6)	73(2)
C(45)	-527(5)	4642(1)	4994(6)	76(2)

C(46)	-971(4)	4483(1)	3966(6)	64(1)
C(47)	-541(3)	4357(1)	3171(5)	48(1)
C(48)	-992(3)	4189(1)	2137(5)	49(1)
C(49)	-593(3)	4066(1)	1354(5)	46(1)
C(50)	-1085(3)	3898(1)	324(6)	60(1)
C(51)	-702(4)	3771(1)	-458(6)	68(2)
C(52)	220(4)	3802(1)	-194(6)	68(2)
C(53)	724(3)	3962(1)	772(6)	54(1)
C(54)	341(3)	4108(1)	1572(5)	45(1)
C(69)	3817(3)	4417(1)	1220(5)	46(1)
C(70)	3156(3)	4388(1)	27(5)	42(1)
C(71)	2905(3)	4586(1)	-880(5)	51(1)
C(72)	3304(4)	4835(1)	-617(6)	65(2)
C(73)	3954(4)	4879(1)	547(7)	69(2)
C(74)	4194(3)	4672(1)	1421(6)	60(2)
C(75)	5737(3)	4007(1)	909(4)	40(1)
C(76)	5468(3)	3837(1)	-158(5)	48(1)
C(77)	5744(4)	3866(1)	-1220(5)	60(1)
C(78)	6284(4)	4074(1)	-1256(5)	64(2)
C(79)	6578(3)	4249(1)	-235(5)	56(1)
C(80)	6308(3)	4212(1)	822(4)	42(1)
C(81)	1330(4)	2672(1)	3713(7)	73(2)
C(82)	1821(4)	2782(2)	4865(7)	89(2)
C(83)	1427(5)	2881(2)	5711(7)	101(3)
C(84)	519(5)	2871(2)	5388(8)	90(2)
C(85)	32(4)	2761(1)	4240(8)	81(2)
C(86)	435(4)	2661(1)	3406(7)	74(2)
C(87)	2243(5)	3506(1)	9295(7)	78(2)
C(88)	2761(5)	3453(1)	10543(8)	84(2)
C(89)	2673(5)	3219(2)	11145(7)	82(2)
C(90)	2051(4)	3029(1)	10484(7)	76(2)
C(91)	1529(4)	3082(1)	9211(7)	66(2)
C(92)	1622(4)	3319(1)	8616(6)	70(2)
C(93)	4054(4)	2524(1)	2344(6)	66(2)
C(94)	4262(4)	2288(1)	1898(6)	69(2)
C(95)	4071(4)	2046(2)	2378(7)	84(2)
C(96)	3651(4)	2053(2)	3338(8)	93(3)
C(97)	3456(4)	2300(2)	3738(7)	85(2)
C(98)	3648(4)	2532(2)	3258(6)	74(2)
Cu(1)	6019(1)	3818(1)	2633(1)	35(1)
Cu(2)	5011(1)	3922(1)	3825(1)	45(1)
Cu(3)	3744(1)	4209(1)	2760(1)	48(1)
C(41)	789(3)	4289(1)	2535(5)	45(1)
S(2)	1949(1)	4387(1)	3079(2)	33(1)
O(2)	2365(2)	4222(1)	2265(4)	42(1)
C(55)	1891(4)	4731(1)	2509(6)	34(1)
C(56)	1456(5)	4797(1)	1195(6)	31(1)
C(57)	1088(4)	4607(1)	167(6)	32(1)
C(58)	669(5)	4688(1)	-1064(7)	38(1)
C(59)	577(6)	4966(1)	-1414(7)	43(1)
C(60)	942(6)	5153(1)	-496(7)	42(2)
C(61)	1393(9)	5077(1)	816(7)	37(1)
C(62)	1785(8)	5273(1)	1759(8)	44(2)
C(63)	2234(9)	5207(1)	3036(7)	46(2)

C(64)	2634(7)	5411(1)	3987(8)	57(2)
C(65)	3129(7)	5344(2)	5204(8)	65(2)
C(66)	3240(6)	5071(2)	5580(7)	63(2)
C(67)	2837(5)	4866(1)	4746(7)	50(2)
C(68)	2318(5)	4927(1)	3438(6)	41(1)
S(2')	1848(2)	4354(1)	2166(4)	34(1)
O(2')	2600(4)	4258(2)	3303(7)	40(3)
C(55')	1831(7)	4716(1)	2052(11)	30(2)
C(56')	1408(15)	4818(2)	785(12)	31(2)
C(57')	1009(13)	4648(2)	-316(13)	33(2)
C(58')	554(14)	4756(3)	-1478(13)	37(3)
C(59')	462(18)	5040(3)	-1668(14)	42(3)
C(60')	790(20)	5208(3)	-648(14)	40(3)
C(61')	1300(30)	5105(2)	594(15)	37(2)
C(62')	1650(20)	5272(2)	1679(17)	43(3)
C(63')	2220(30)	5173(2)	2840(20)	47(2)
C(64')	2720(20)	5354(2)	3846(19)	55(3)
C(65')	3200(20)	5263(3)	5034(18)	61(3)
C(66')	3287(16)	4983(3)	5259(15)	58(3)
C(67')	2884(13)	4798(3)	4312(15)	50(3)
C(68')	2300(16)	4883(2)	3079(14)	42(2)
Cu(4)	4796(1)	4139(1)	1589(1)	43(1)
F(1)	7024(2)	3917(1)	5648(3)	53(1)
F(2)	7361(2)	3558(1)	7609(3)	61(1)
F(3)	6538(2)	3076(1)	7252(3)	71(1)
F(4)	5352(2)	2947(1)	4903(3)	67(1)
F(5)	4950(2)	3308(1)	2962(3)	55(1)
F(6)	4792(3)	4457(1)	5394(4)	94(1)
F(7)	4341(3)	4421(1)	7585(4)	108(2)
F(8)	3453(3)	3982(1)	7957(4)	109(2)
F(9)	3016(3)	3578(1)	6195(4)	97(1)
F(10)	3395(2)	3623(1)	3989(4)	80(1)
F(11)	2733(2)	4147(1)	-251(3)	55(1)
F(12)	2285(2)	4541(1)	-2039(3)	70(1)
F(13)	3060(3)	5033(1)	-1500(4)	104(1)
F(14)	4353(3)	5122(1)	794(5)	111(2)
F(15)	4843(2)	4726(1)	2562(4)	96(1)
F(16)	4918(2)	3628(1)	-165(3)	65(1)
F(17)	5470(3)	3690(1)	-2215(3)	89(1)
F(18)	6544(3)	4110(1)	-2297(4)	102(1)
F(19)	7131(2)	4452(1)	-257(4)	82(1)
F(20)	6628(2)	4384(1)	1832(3)	58(1)
O(1)	7238(2)	3685(1)	2523(3)	40(1)
S(1)	7846(1)	3543(1)	3726(1)	37(1)

Table S3. Bond lengths [A] and angles [deg] for **8**.

C(1)-C(2)	1.416(6)
C(1)-C(14)	1.418(6)
C(1)-S(1)	1.799(4)
C(2)-C(7)	1.428(6)
C(2)-C(3)	1.429(7)
C(3)-C(4)	1.355(7)
C(3)-H(3)	0.9500
C(4)-C(5)	1.415(8)
C(4)-H(4)	0.9500
C(5)-C(6)	1.344(9)
C(5)-H(5)	0.9500
C(6)-C(7)	1.424(7)
C(6)-H(6)	0.9500
C(7)-C(8)	1.386(7)
C(8)-C(9)	1.391(7)
C(8)-H(8)	0.9500
C(9)-C(10)	1.435(7)
C(9)-C(14)	1.446(6)
C(10)-C(11)	1.332(9)
C(10)-H(10)	0.9500
C(11)-C(12)	1.413(8)
C(11)-H(11)	0.9500
C(12)-C(13)	1.365(7)
C(12)-H(12)	0.9500
C(13)-C(14)	1.420(6)
C(13)-H(13)	0.9500
C(15)-C(16)	1.409(7)
C(15)-C(28)	1.409(7)
C(15)-S(1)	1.796(4)
C(16)-C(17)	1.411(7)
C(16)-C(21)	1.441(6)
C(17)-C(18)	1.373(7)
C(17)-H(17)	0.9500
C(18)-C(19)	1.415(8)
C(18)-H(18)	0.9500
C(19)-C(20)	1.340(9)
C(19)-H(19)	0.9500
C(20)-C(21)	1.428(8)
C(20)-H(20)	0.9500
C(21)-C(22)	1.386(8)
C(22)-C(23)	1.370(9)
C(22)-H(22)	0.9500
C(23)-C(24)	1.442(9)
C(23)-C(28)	1.446(7)
C(24)-C(25)	1.341(11)
C(24)-H(24)	0.9500
C(25)-C(26)	1.418(11)
C(25)-H(25)	0.9500
C(26)-C(27)	1.369(7)
C(26)-H(26)	0.9500
C(27)-C(28)	1.429(8)

C(27)-H(27)	0.9500
C(29)-C(34)	1.390(6)
C(29)-C(30)	1.395(6)
C(29)-Cu(1)	1.993(4)
C(29)-Cu(2)	2.024(4)
C(30)-F(1)	1.357(5)
C(30)-C(31)	1.371(6)
C(31)-F(2)	1.337(5)
C(31)-C(32)	1.373(7)
C(32)-F(3)	1.345(5)
C(32)-C(33)	1.372(7)
C(33)-F(4)	1.348(5)
C(33)-C(34)	1.372(6)
C(34)-F(5)	1.349(5)
C(35)-C(36)	1.369(8)
C(35)-C(40)	1.411(8)
C(35)-Cu(2)	1.991(5)
C(35)-Cu(3)	2.043(6)
C(36)-F(6)	1.344(7)
C(36)-C(37)	1.409(9)
C(37)-C(38)	1.347(10)
C(37)-F(7)	1.351(7)
C(38)-F(8)	1.338(7)
C(38)-C(39)	1.370(9)
C(39)-F(9)	1.358(7)
C(39)-C(40)	1.359(8)
C(40)-F(10)	1.349(6)
C(42)-C(47)	1.440(6)
C(42)-C(43)	1.441(7)
C(42)-C(41)	1.441(7)
C(43)-C(44)	1.360(8)
C(43)-H(43)	0.9500
C(44)-C(45)	1.399(9)
C(44)-H(44)	0.9500
C(45)-C(46)	1.364(8)
C(45)-H(45)	0.9500
C(46)-C(47)	1.417(7)
C(46)-H(46)	0.9500
C(47)-C(48)	1.397(7)
C(48)-C(49)	1.366(7)
C(48)-H(48)	0.9500
C(49)-C(50)	1.413(7)
C(49)-C(54)	1.449(6)
C(50)-C(51)	1.356(8)
C(50)-H(50)	0.9500
C(51)-C(52)	1.416(8)
C(51)-H(51)	0.9500
C(52)-C(53)	1.354(7)
C(52)-H(52)	0.9500
C(53)-C(54)	1.420(7)
C(53)-H(53)	0.9500
C(54)-C(41)	1.386(7)
C(69)-C(74)	1.385(6)
C(69)-C(70)	1.391(7)

C(69)-Cu(3)	2.007(5)
C(69)-Cu(4)	2.021(4)
C(70)-F(11)	1.355(5)
C(70)-C(71)	1.357(7)
C(71)-F(12)	1.347(6)
C(71)-C(72)	1.369(7)
C(72)-F(13)	1.339(6)
C(72)-C(73)	1.371(9)
C(73)-F(14)	1.348(6)
C(73)-C(74)	1.363(8)
C(74)-F(15)	1.361(6)
C(75)-C(76)	1.384(7)
C(75)-C(80)	1.390(6)
C(75)-Cu(4)	1.997(4)
C(75)-Cu(1)	2.016(4)
C(76)-F(16)	1.355(6)
C(76)-C(77)	1.377(7)
C(77)-F(17)	1.348(6)
C(77)-C(78)	1.354(8)
C(78)-F(18)	1.345(6)
C(78)-C(79)	1.364(8)
C(79)-F(19)	1.345(6)
C(79)-C(80)	1.372(6)
C(80)-F(20)	1.353(5)
C(81)-C(86)	1.361(8)
C(81)-C(82)	1.361(9)
C(81)-H(81)	0.9500
C(82)-C(83)	1.368(10)
C(82)-H(82)	0.9500
C(83)-C(84)	1.379(10)
C(83)-H(83)	0.9500
C(84)-C(85)	1.355(10)
C(84)-H(84)	0.9500
C(85)-C(86)	1.369(9)
C(85)-H(85)	0.9500
C(86)-H(86)	0.9500
C(87)-C(88)	1.369(10)
C(87)-C(92)	1.385(9)
C(87)-H(87)	0.9500
C(88)-C(89)	1.360(10)
C(88)-H(88)	0.9500
C(89)-C(90)	1.386(9)
C(89)-H(89)	0.9500
C(90)-C(91)	1.392(9)
C(90)-H(90)	0.9500
C(91)-C(92)	1.370(9)
C(91)-H(91)	0.9500
C(92)-H(92)	0.9500
C(93)-C(94)	1.350(9)
C(93)-C(98)	1.359(8)
C(93)-H(93)	0.9500
C(94)-C(95)	1.376(9)
C(94)-H(94)	0.9500
C(95)-C(96)	1.420(11)

C(95)-H(95)	0.9500
C(96)-C(97)	1.367(11)
C(96)-H(96)	0.9500
C(97)-C(98)	1.337(10)
C(97)-H(97)	0.9500
C(98)-H(98)	0.9500
Cu(1)-O(1)	2.099(3)
Cu(1)-Cu(2)	2.4374(8)
Cu(1)-Cu(4)	2.4896(8)
Cu(2)-Cu(3)	2.4353(9)
Cu(2)-Cu(4)	2.5867(10)
Cu(3)-O(2)	2.095(4)
Cu(3)-O(2')	2.116(5)
Cu(3)-Cu(4)	2.4498(9)
C(41)-S(2)	1.821(4)
C(41)-S(2')	1.895(5)
S(2)-O(2)	1.514(4)
S(2)-C(55)	1.803(5)
C(55)-C(68)	1.406(7)
C(55)-C(56)	1.412(8)
C(56)-C(57)	1.433(7)
C(56)-C(61)	1.441(7)
C(57)-C(58)	1.351(8)
C(57)-H(57)	0.9500
C(58)-C(59)	1.421(8)
C(58)-H(58)	0.9500
C(59)-C(60)	1.347(7)
C(59)-H(59)	0.9500
C(60)-C(61)	1.427(8)
C(60)-H(60)	0.9500
C(61)-C(62)	1.401(7)
C(62)-C(63)	1.382(8)
C(62)-H(62)	0.9500
C(63)-C(64)	1.438(7)
C(63)-C(68)	1.444(7)
C(64)-C(65)	1.347(9)
C(64)-H(64)	0.9500
C(65)-C(66)	1.403(10)
C(65)-H(65)	0.9500
C(66)-C(67)	1.373(9)
C(66)-H(66)	0.9500
C(67)-C(68)	1.429(8)
C(67)-H(67)	0.9500
S(2')-O(2')	1.497(5)
S(2')-C(55')	1.789(6)
C(55')-C(68')	1.399(8)
C(55')-C(56')	1.420(9)
C(56')-C(61')	1.433(7)
C(56')-C(57')	1.435(8)
C(57')-C(58')	1.349(10)
C(57')-H(57')	0.9500
C(58')-C(59')	1.421(9)
C(58')-H(58')	0.9500
C(59')-C(60')	1.348(8)

C(59')-H(59')	0.9500
C(60')-C(61')	1.427(9)
C(60')-H(60')	0.9500
C(61')-C(62')	1.401(7)
C(62')-C(63')	1.386(9)
C(62')-H(62')	0.9500
C(63')-C(64')	1.438(8)
C(63')-C(68')	1.451(8)
C(64')-C(65')	1.348(10)
C(64')-H(64')	0.9500
C(65')-C(66')	1.404(11)
C(65')-H(65')	0.9500
C(66')-C(67')	1.373(10)
C(66')-H(66')	0.9500
C(67')-C(68')	1.428(9)
C(67')-H(67')	0.9500
O(1)-S(1)	1.525(3)

C(2)-C(1)-C(14)	122.2(4)
C(2)-C(1)-S(1)	112.6(3)
C(14)-C(1)-S(1)	124.8(3)
C(1)-C(2)-C(7)	118.8(4)
C(1)-C(2)-C(3)	124.2(4)
C(7)-C(2)-C(3)	117.0(4)
C(4)-C(3)-C(2)	121.5(5)
C(4)-C(3)-H(3)	119.3
C(2)-C(3)-H(3)	119.3
C(3)-C(4)-C(5)	121.1(5)
C(3)-C(4)-H(4)	119.5
C(5)-C(4)-H(4)	119.5
C(6)-C(5)-C(4)	119.5(5)
C(6)-C(5)-H(5)	120.2
C(4)-C(5)-H(5)	120.2
C(5)-C(6)-C(7)	121.5(5)
C(5)-C(6)-H(6)	119.2
C(7)-C(6)-H(6)	119.2
C(8)-C(7)-C(6)	121.5(5)
C(8)-C(7)-C(2)	119.0(5)
C(6)-C(7)-C(2)	119.5(5)
C(7)-C(8)-C(9)	122.9(4)
C(7)-C(8)-H(8)	118.5
C(9)-C(8)-H(8)	118.5
C(8)-C(9)-C(10)	121.4(5)
C(8)-C(9)-C(14)	119.6(4)
C(10)-C(9)-C(14)	118.9(5)
C(11)-C(10)-C(9)	121.9(5)
C(11)-C(10)-H(10)	119.1
C(9)-C(10)-H(10)	119.1
C(10)-C(11)-C(12)	119.6(5)
C(10)-C(11)-H(11)	120.2
C(12)-C(11)-H(11)	120.2
C(13)-C(12)-C(11)	121.1(6)
C(13)-C(12)-H(12)	119.4
C(11)-C(12)-H(12)	119.4

C(12)-C(13)-C(14)	121.7(5)
C(12)-C(13)-H(13)	119.1
C(14)-C(13)-H(13)	119.1
C(1)-C(14)-C(13)	126.2(4)
C(1)-C(14)-C(9)	117.2(4)
C(13)-C(14)-C(9)	116.6(4)
C(16)-C(15)-C(28)	122.9(4)
C(16)-C(15)-S(1)	124.4(3)
C(28)-C(15)-S(1)	112.7(4)
C(15)-C(16)-C(17)	124.6(4)
C(15)-C(16)-C(21)	118.2(5)
C(17)-C(16)-C(21)	117.2(5)
C(18)-C(17)-C(16)	122.0(5)
C(18)-C(17)-H(17)	119.0
C(16)-C(17)-H(17)	119.0
C(17)-C(18)-C(19)	119.9(6)
C(17)-C(18)-H(18)	120.0
C(19)-C(18)-H(18)	120.0
C(20)-C(19)-C(18)	120.4(6)
C(20)-C(19)-H(19)	119.8
C(18)-C(19)-H(19)	119.8
C(19)-C(20)-C(21)	121.3(5)
C(19)-C(20)-H(20)	119.4
C(21)-C(20)-H(20)	119.4
C(22)-C(21)-C(20)	122.5(5)
C(22)-C(21)-C(16)	118.5(5)
C(20)-C(21)-C(16)	119.1(5)
C(23)-C(22)-C(21)	123.4(5)
C(23)-C(22)-H(22)	118.3
C(21)-C(22)-H(22)	118.3
C(22)-C(23)-C(24)	123.0(6)
C(22)-C(23)-C(28)	120.0(5)
C(24)-C(23)-C(28)	116.9(6)
C(25)-C(24)-C(23)	123.1(7)
C(25)-C(24)-H(24)	118.5
C(23)-C(24)-H(24)	118.5
C(24)-C(25)-C(26)	119.5(6)
C(24)-C(25)-H(25)	120.2
C(26)-C(25)-H(25)	120.2
C(27)-C(26)-C(25)	120.8(7)
C(27)-C(26)-H(26)	119.6
C(25)-C(26)-H(26)	119.6
C(26)-C(27)-C(28)	121.1(6)
C(26)-C(27)-H(27)	119.4
C(28)-C(27)-H(27)	119.4
C(15)-C(28)-C(27)	124.7(5)
C(15)-C(28)-C(23)	116.8(5)
C(27)-C(28)-C(23)	118.5(5)
C(34)-C(29)-C(30)	113.8(4)
C(34)-C(29)-Cu(1)	118.9(3)
C(30)-C(29)-Cu(1)	122.0(3)
C(34)-C(29)-Cu(2)	109.2(3)
C(30)-C(29)-Cu(2)	109.3(3)
Cu(1)-C(29)-Cu(2)	74.69(15)

F(1)-C(30)-C(31)	117.2(4)
F(1)-C(30)-C(29)	118.5(4)
C(31)-C(30)-C(29)	124.3(4)
F(2)-C(31)-C(30)	121.9(4)
F(2)-C(31)-C(32)	119.2(4)
C(30)-C(31)-C(32)	118.9(4)
F(3)-C(32)-C(33)	120.8(4)
F(3)-C(32)-C(31)	119.5(4)
C(33)-C(32)-C(31)	119.7(4)
F(4)-C(33)-C(32)	119.2(4)
F(4)-C(33)-C(34)	121.1(4)
C(32)-C(33)-C(34)	119.7(4)
F(5)-C(34)-C(33)	116.9(4)
F(5)-C(34)-C(29)	119.5(4)
C(33)-C(34)-C(29)	123.6(4)
C(36)-C(35)-C(40)	113.5(5)
C(36)-C(35)-Cu(2)	118.9(4)
C(40)-C(35)-Cu(2)	111.5(4)
C(36)-C(35)-Cu(3)	113.4(5)
C(40)-C(35)-Cu(3)	120.0(4)
Cu(2)-C(35)-Cu(3)	74.25(18)
F(6)-C(36)-C(35)	118.8(6)
F(6)-C(36)-C(37)	117.3(6)
C(35)-C(36)-C(37)	123.8(6)
C(38)-C(37)-F(7)	120.1(6)
C(38)-C(37)-C(36)	118.9(6)
F(7)-C(37)-C(36)	121.1(7)
F(8)-C(38)-C(37)	120.1(6)
F(8)-C(38)-C(39)	119.7(7)
C(37)-C(38)-C(39)	120.2(6)
F(9)-C(39)-C(40)	120.9(6)
F(9)-C(39)-C(38)	119.5(6)
C(40)-C(39)-C(38)	119.5(6)
F(10)-C(40)-C(39)	117.5(5)
F(10)-C(40)-C(35)	118.4(5)
C(39)-C(40)-C(35)	123.8(6)
C(47)-C(42)-C(43)	116.0(5)
C(47)-C(42)-C(41)	117.9(4)
C(43)-C(42)-C(41)	126.1(4)
C(44)-C(43)-C(42)	122.0(5)
C(44)-C(43)-H(43)	119.0
C(42)-C(43)-H(43)	119.0
C(43)-C(44)-C(45)	121.4(6)
C(43)-C(44)-H(44)	119.3
C(45)-C(44)-H(44)	119.3
C(46)-C(45)-C(44)	119.0(6)
C(46)-C(45)-H(45)	120.5
C(44)-C(45)-H(45)	120.5
C(45)-C(46)-C(47)	122.1(6)
C(45)-C(46)-H(46)	119.0
C(47)-C(46)-H(46)	119.0
C(48)-C(47)-C(46)	122.0(5)
C(48)-C(47)-C(42)	118.5(5)
C(46)-C(47)-C(42)	119.4(5)

C(49)-C(48)-C(47)	123.0(4)
C(49)-C(48)-H(48)	118.5
C(47)-C(48)-H(48)	118.5
C(48)-C(49)-C(50)	120.4(4)
C(48)-C(49)-C(54)	120.2(4)
C(50)-C(49)-C(54)	119.4(5)
C(51)-C(50)-C(49)	121.6(5)
C(51)-C(50)-H(50)	119.2
C(49)-C(50)-H(50)	119.2
C(50)-C(51)-C(52)	118.6(5)
C(50)-C(51)-H(51)	120.7
C(52)-C(51)-H(51)	120.7
C(53)-C(52)-C(51)	122.4(5)
C(53)-C(52)-H(52)	118.8
C(51)-C(52)-H(52)	118.8
C(52)-C(53)-C(54)	120.7(5)
C(52)-C(53)-H(53)	119.7
C(54)-C(53)-H(53)	119.7
C(41)-C(54)-C(53)	125.1(4)
C(41)-C(54)-C(49)	117.8(4)
C(53)-C(54)-C(49)	117.1(4)
C(74)-C(69)-C(70)	113.6(5)
C(74)-C(69)-Cu(3)	118.5(4)
C(70)-C(69)-Cu(3)	119.8(3)
C(74)-C(69)-Cu(4)	108.5(3)
C(70)-C(69)-Cu(4)	114.7(3)
Cu(3)-C(69)-Cu(4)	74.91(16)
F(11)-C(70)-C(71)	117.7(4)
F(11)-C(70)-C(69)	118.1(4)
C(71)-C(70)-C(69)	124.1(4)
F(12)-C(71)-C(70)	121.1(5)
F(12)-C(71)-C(72)	119.6(5)
C(70)-C(71)-C(72)	119.2(5)
F(13)-C(72)-C(71)	119.8(6)
F(13)-C(72)-C(73)	120.4(5)
C(71)-C(72)-C(73)	119.8(5)
F(14)-C(73)-C(74)	121.5(6)
F(14)-C(73)-C(72)	119.5(6)
C(74)-C(73)-C(72)	119.0(5)
F(15)-C(74)-C(73)	116.9(5)
F(15)-C(74)-C(69)	118.9(5)
C(73)-C(74)-C(69)	124.2(5)
C(76)-C(75)-C(80)	114.5(4)
C(76)-C(75)-Cu(4)	116.6(3)
C(80)-C(75)-Cu(4)	112.2(3)
C(76)-C(75)-Cu(1)	114.8(3)
C(80)-C(75)-Cu(1)	116.8(3)
Cu(4)-C(75)-Cu(1)	76.69(16)
F(16)-C(76)-C(77)	117.4(5)
F(16)-C(76)-C(75)	119.2(4)
C(77)-C(76)-C(75)	123.4(5)
F(17)-C(77)-C(78)	120.7(5)
F(17)-C(77)-C(76)	120.2(6)
C(78)-C(77)-C(76)	119.1(5)

F(18)-C(78)-C(77)	120.1(6)
F(18)-C(78)-C(79)	119.3(6)
C(77)-C(78)-C(79)	120.6(5)
F(19)-C(79)-C(78)	120.7(5)
F(19)-C(79)-C(80)	120.3(5)
C(78)-C(79)-C(80)	119.0(5)
F(20)-C(80)-C(79)	117.7(4)
F(20)-C(80)-C(75)	119.0(4)
C(79)-C(80)-C(75)	123.3(5)
C(86)-C(81)-C(82)	119.1(6)
C(86)-C(81)-H(81)	120.5
C(82)-C(81)-H(81)	120.5
C(81)-C(82)-C(83)	120.9(6)
C(81)-C(82)-H(82)	119.5
C(83)-C(82)-H(82)	119.5
C(82)-C(83)-C(84)	119.6(7)
C(82)-C(83)-H(83)	120.2
C(84)-C(83)-H(83)	120.2
C(85)-C(84)-C(83)	119.4(7)
C(85)-C(84)-H(84)	120.3
C(83)-C(84)-H(84)	120.3
C(84)-C(85)-C(86)	120.4(6)
C(84)-C(85)-H(85)	119.8
C(86)-C(85)-H(85)	119.8
C(81)-C(86)-C(85)	120.6(6)
C(81)-C(86)-H(86)	119.7
C(85)-C(86)-H(86)	119.7
C(88)-C(87)-C(92)	119.8(7)
C(88)-C(87)-H(87)	120.1
C(92)-C(87)-H(87)	120.1
C(89)-C(88)-C(87)	121.4(7)
C(89)-C(88)-H(88)	119.3
C(87)-C(88)-H(88)	119.3
C(88)-C(89)-C(90)	119.6(7)
C(88)-C(89)-H(89)	120.2
C(90)-C(89)-H(89)	120.2
C(89)-C(90)-C(91)	119.1(6)
C(89)-C(90)-H(90)	120.5
C(91)-C(90)-H(90)	120.5
C(92)-C(91)-C(90)	120.8(6)
C(92)-C(91)-H(91)	119.6
C(90)-C(91)-H(91)	119.6
C(91)-C(92)-C(87)	119.3(7)
C(91)-C(92)-H(92)	120.4
C(87)-C(92)-H(92)	120.4
C(94)-C(93)-C(98)	121.5(6)
C(94)-C(93)-H(93)	119.3
C(98)-C(93)-H(93)	119.3
C(93)-C(94)-C(95)	120.1(6)
C(93)-C(94)-H(94)	120.0
C(95)-C(94)-H(94)	120.0
C(94)-C(95)-C(96)	118.7(7)
C(94)-C(95)-H(95)	120.6
C(96)-C(95)-H(95)	120.6

C(97)-C(96)-C(95)	117.9(7)
C(97)-C(96)-H(96)	121.0
C(95)-C(96)-H(96)	121.0
C(98)-C(97)-C(96)	122.3(7)
C(98)-C(97)-H(97)	118.8
C(96)-C(97)-H(97)	118.8
C(97)-C(98)-C(93)	119.4(7)
C(97)-C(98)-H(98)	120.3
C(93)-C(98)-H(98)	120.3
C(29)-Cu(1)-C(75)	165.07(17)
C(29)-Cu(1)-O(1)	102.64(14)
C(75)-Cu(1)-O(1)	91.05(14)
C(29)-Cu(1)-Cu(2)	53.24(12)
C(75)-Cu(1)-Cu(2)	114.65(12)
O(1)-Cu(1)-Cu(2)	152.51(9)
C(29)-Cu(1)-Cu(4)	115.87(12)
C(75)-Cu(1)-Cu(4)	51.32(12)
O(1)-Cu(1)-Cu(4)	141.21(8)
Cu(2)-Cu(1)-Cu(4)	63.33(3)
C(35)-Cu(2)-C(29)	136.7(2)
C(35)-Cu(2)-Cu(3)	53.86(17)
C(29)-Cu(2)-Cu(3)	163.95(13)
C(35)-Cu(2)-Cu(1)	171.18(18)
C(29)-Cu(2)-Cu(1)	52.07(12)
Cu(3)-Cu(2)-Cu(1)	117.46(4)
C(35)-Cu(2)-Cu(4)	111.89(18)
C(29)-Cu(2)-Cu(4)	110.76(12)
Cu(3)-Cu(2)-Cu(4)	58.30(3)
Cu(1)-Cu(2)-Cu(4)	59.32(2)
C(69)-Cu(3)-C(35)	160.1(2)
C(69)-Cu(3)-O(2)	96.54(17)
C(35)-Cu(3)-O(2)	101.55(18)
C(69)-Cu(3)-O(2')	118.0(2)
C(35)-Cu(3)-O(2')	76.4(2)
C(69)-Cu(3)-Cu(2)	115.50(12)
C(35)-Cu(3)-Cu(2)	51.90(14)
O(2)-Cu(3)-Cu(2)	141.55(13)
O(2')-Cu(3)-Cu(2)	126.47(19)
C(69)-Cu(3)-Cu(4)	52.79(12)
C(35)-Cu(3)-Cu(4)	115.56(14)
O(2)-Cu(3)-Cu(4)	135.71(11)
O(2')-Cu(3)-Cu(4)	165.6(2)
Cu(2)-Cu(3)-Cu(4)	63.94(3)
C(54)-C(41)-C(42)	122.1(4)
C(54)-C(41)-S(2)	129.7(4)
C(42)-C(41)-S(2)	107.8(3)
C(54)-C(41)-S(2')	103.0(4)
C(42)-C(41)-S(2')	134.4(4)
O(2)-S(2)-C(55)	106.8(3)
O(2)-S(2)-C(41)	105.2(2)
C(55)-S(2)-C(41)	102.0(2)
S(2)-O(2)-Cu(3)	119.6(2)
C(68)-C(55)-C(56)	122.6(4)
C(68)-C(55)-S(2)	116.2(4)

C(56)-C(55)-S(2)	121.2(4)
C(55)-C(56)-C(57)	125.9(5)
C(55)-C(56)-C(61)	118.4(5)
C(57)-C(56)-C(61)	115.7(5)
C(58)-C(57)-C(56)	121.9(5)
C(58)-C(57)-H(57)	119.1
C(56)-C(57)-H(57)	119.1
C(57)-C(58)-C(59)	121.9(5)
C(57)-C(58)-H(58)	119.1
C(59)-C(58)-H(58)	119.1
C(60)-C(59)-C(58)	118.8(6)
C(60)-C(59)-H(59)	120.6
C(58)-C(59)-H(59)	120.6
C(59)-C(60)-C(61)	121.4(6)
C(59)-C(60)-H(60)	119.3
C(61)-C(60)-H(60)	119.3
C(62)-C(61)-C(60)	120.9(5)
C(62)-C(61)-C(56)	118.7(5)
C(60)-C(61)-C(56)	120.3(5)
C(63)-C(62)-C(61)	122.5(5)
C(63)-C(62)-H(62)	118.7
C(61)-C(62)-H(62)	118.7
C(62)-C(63)-C(64)	121.7(5)
C(62)-C(63)-C(68)	119.9(5)
C(64)-C(63)-C(68)	118.4(5)
C(65)-C(64)-C(63)	121.3(6)
C(65)-C(64)-H(64)	119.3
C(63)-C(64)-H(64)	119.3
C(64)-C(65)-C(66)	120.1(6)
C(64)-C(65)-H(65)	120.0
C(66)-C(65)-H(65)	120.0
C(67)-C(66)-C(65)	121.8(7)
C(67)-C(66)-H(66)	119.1
C(65)-C(66)-H(66)	119.1
C(66)-C(67)-C(68)	120.0(6)
C(66)-C(67)-H(67)	120.0
C(68)-C(67)-H(67)	120.0
C(55)-C(68)-C(67)	124.2(5)
C(55)-C(68)-C(63)	117.7(5)
C(67)-C(68)-C(63)	118.1(5)
O(2')-S(2')-C(55')	111.4(5)
O(2')-S(2')-C(41)	107.6(3)
C(55')-S(2')-C(41)	101.0(4)
S(2')-O(2')-Cu(3)	109.8(4)
C(68')-C(55')-C(56')	121.8(6)
C(68')-C(55')-S(2')	123.0(6)
C(56')-C(55')-S(2')	114.6(6)
C(55')-C(56')-C(61')	119.5(6)
C(55')-C(56')-C(57')	123.1(7)
C(61')-C(56')-C(57')	117.2(7)
C(58')-C(57')-C(56')	120.8(7)
C(58')-C(57')-H(57')	119.6
C(56')-C(57')-H(57')	119.6
C(57')-C(58')-C(59')	121.8(7)

C(57')-C(58')-H(58')	119.1
C(59')-C(58')-H(58')	119.1
C(60')-C(59')-C(58')	119.5(8)
C(60')-C(59')-H(59')	120.3
C(58')-C(59')-H(59')	120.3
C(59')-C(60')-C(61')	120.9(8)
C(59')-C(60')-H(60')	119.5
C(61')-C(60')-H(60')	119.5
C(62')-C(61')-C(60')	122.6(7)
C(62')-C(61')-C(56')	117.5(6)
C(60')-C(61')-C(56')	119.6(6)
C(63')-C(62')-C(61')	121.9(9)
C(63')-C(62')-H(62')	119.1
C(61')-C(62')-H(62')	119.1
C(62')-C(63')-C(64')	120.8(7)
C(62')-C(63')-C(68')	120.4(7)
C(64')-C(63')-C(68')	118.8(7)
C(65')-C(64')-C(63')	121.7(8)
C(65')-C(64')-H(64')	119.1
C(63')-C(64')-H(64')	119.1
C(64')-C(65')-C(66')	119.3(9)
C(64')-C(65')-H(65')	120.4
C(66')-C(65')-H(65')	120.4
C(67')-C(66')-C(65')	121.9(8)
C(67')-C(66')-H(66')	119.1
C(65')-C(66')-H(66')	119.1
C(66')-C(67')-C(68')	121.0(8)
C(66')-C(67')-H(67')	119.5
C(68')-C(67')-H(67')	119.5
C(55')-C(68')-C(67')	126.5(7)
C(55')-C(68')-C(63')	116.6(6)
C(67')-C(68')-C(63')	116.8(7)
C(75)-Cu(4)-C(69)	141.2(2)
C(75)-Cu(4)-Cu(3)	166.38(13)
C(69)-Cu(4)-Cu(3)	52.30(15)
C(75)-Cu(4)-Cu(1)	51.99(12)
C(69)-Cu(4)-Cu(1)	165.13(16)
Cu(3)-Cu(4)-Cu(1)	114.96(3)
C(75)-Cu(4)-Cu(2)	109.34(12)
C(69)-Cu(4)-Cu(2)	108.95(15)
Cu(3)-Cu(4)-Cu(2)	57.76(3)
Cu(1)-Cu(4)-Cu(2)	57.35(2)
S(1)-O(1)-Cu(1)	115.94(16)
O(1)-S(1)-C(15)	107.79(19)
O(1)-S(1)-C(1)	111.01(19)
C(15)-S(1)-C(1)	103.53(19)

Symmetry transformations used to generate equivalent atoms:

Table S4. Anisotropic displacement parameters (A^2 x 10^3) for **8**.
The anisotropic displacement factor exponent takes the form:
-2 pi^2 [h^2 a*^2 U11 + ... + 2 h k a* b* U12]

	U11	U22	U33	U23	U13	U12
C(1)	29(2)	36(2)	46(2)	-1(2)	17(2)	3(2)
C(2)	35(2)	44(2)	44(2)	4(2)	23(2)	10(2)
C(3)	45(3)	53(3)	47(3)	3(2)	17(2)	8(2)
C(4)	57(3)	77(4)	41(3)	4(3)	16(2)	19(3)
C(5)	84(4)	74(4)	54(3)	25(3)	30(3)	35(3)
C(6)	79(4)	53(3)	71(4)	20(3)	40(3)	20(3)
C(7)	51(3)	41(2)	63(3)	8(2)	31(2)	11(2)
C(8)	59(3)	38(2)	76(4)	4(2)	34(3)	2(2)
C(9)	41(2)	42(2)	71(3)	-12(2)	26(2)	-3(2)
C(10)	58(3)	48(3)	93(5)	-18(3)	27(3)	-10(2)
C(11)	49(3)	66(4)	87(5)	-34(3)	13(3)	-11(3)
C(12)	47(3)	70(4)	58(3)	-16(3)	9(2)	2(2)
C(13)	37(2)	49(3)	45(3)	-11(2)	7(2)	-1(2)
C(14)	30(2)	43(2)	49(3)	-5(2)	19(2)	2(2)
C(15)	31(2)	41(2)	50(3)	10(2)	9(2)	-3(2)
C(16)	41(2)	47(3)	54(3)	16(2)	24(2)	4(2)
C(17)	47(3)	66(3)	48(3)	6(2)	22(2)	1(2)
C(18)	74(4)	79(4)	60(3)	1(3)	36(3)	2(3)
C(19)	80(4)	94(5)	65(4)	10(3)	44(4)	22(4)
C(20)	56(3)	87(4)	83(4)	18(4)	46(3)	16(3)
C(21)	38(3)	66(3)	71(4)	28(3)	18(3)	8(2)
C(22)	34(3)	72(4)	92(4)	32(3)	17(3)	3(2)
C(23)	39(3)	54(3)	72(4)	20(3)	-7(3)	-9(2)
C(24)	49(3)	71(4)	114(6)	19(4)	-11(4)	-16(3)
C(25)	81(5)	67(4)	95(5)	1(4)	-26(4)	-26(4)
C(26)	93(5)	52(3)	91(5)	-18(3)	-13(4)	-12(3)
C(27)	60(3)	50(3)	68(4)	-5(3)	-7(3)	-11(2)
C(28)	43(3)	38(2)	65(3)	9(2)	7(2)	-3(2)
C(29)	37(2)	40(2)	39(2)	0(2)	19(2)	-2(2)
C(30)	39(2)	36(2)	46(2)	-5(2)	22(2)	-3(2)
C(31)	40(2)	52(3)	36(2)	-6(2)	15(2)	3(2)
C(32)	52(3)	51(3)	42(3)	10(2)	23(2)	6(2)
C(33)	48(3)	36(2)	56(3)	3(2)	27(2)	-6(2)
C(34)	34(2)	46(2)	41(2)	-6(2)	15(2)	-3(2)
C(35)	49(3)	76(4)	62(3)	-16(3)	31(3)	5(3)
C(36)	56(3)	74(4)	90(5)	-14(3)	36(3)	4(3)
C(37)	68(4)	92(5)	64(4)	-11(3)	30(3)	26(3)
C(38)	65(4)	101(5)	63(4)	-7(4)	24(3)	31(4)
C(39)	47(3)	79(4)	93(5)	18(4)	37(3)	13(3)
C(40)	57(3)	62(3)	62(3)	-3(3)	33(3)	4(3)
C(42)	41(2)	41(2)	52(3)	7(2)	12(2)	-1(2)
C(43)	56(3)	52(3)	63(3)	6(3)	4(3)	-9(2)
C(44)	96(5)	66(4)	60(4)	-9(3)	30(3)	-6(3)
C(45)	84(5)	84(4)	64(4)	-9(3)	31(3)	-5(4)
C(46)	61(3)	73(4)	67(4)	3(3)	34(3)	0(3)

C(47)	43(2)	47(3)	53(3)	10(2)	13(2)	-2(2)
C(48)	32(2)	49(3)	67(3)	13(2)	17(2)	0(2)
C(49)	33(2)	35(2)	66(3)	8(2)	9(2)	-3(2)
C(50)	38(3)	45(3)	89(4)	-8(3)	9(3)	-4(2)
C(51)	58(3)	58(3)	82(4)	-28(3)	12(3)	-11(3)
C(52)	60(3)	55(3)	92(4)	-21(3)	30(3)	-3(3)
C(53)	45(3)	37(2)	81(4)	-8(2)	23(3)	-5(2)
C(54)	34(2)	31(2)	70(3)	7(2)	16(2)	2(2)
C(69)	29(2)	33(2)	75(3)	-2(2)	14(2)	1(2)
C(70)	35(2)	37(2)	61(3)	-11(2)	24(2)	-2(2)
C(71)	48(3)	58(3)	54(3)	-2(2)	26(2)	2(2)
C(72)	76(4)	51(3)	75(4)	11(3)	34(3)	-1(3)
C(73)	57(3)	39(3)	110(5)	-4(3)	23(3)	-13(2)
C(74)	33(2)	41(3)	93(4)	-7(3)	4(3)	-4(2)
C(75)	34(2)	42(2)	45(2)	7(2)	16(2)	11(2)
C(76)	39(2)	47(3)	53(3)	3(2)	10(2)	12(2)
C(77)	54(3)	82(4)	45(3)	-6(3)	19(2)	25(3)
C(78)	68(4)	88(4)	49(3)	18(3)	35(3)	28(3)
C(79)	44(3)	67(3)	66(3)	23(3)	32(3)	11(2)
C(80)	36(2)	48(3)	45(3)	8(2)	18(2)	8(2)
C(81)	65(4)	82(4)	78(4)	-4(3)	31(3)	5(3)
C(82)	50(3)	121(6)	83(5)	-14(4)	4(3)	21(4)
C(83)	80(5)	138(7)	70(4)	-26(4)	4(4)	36(5)
C(84)	100(6)	91(5)	92(5)	-6(4)	50(5)	22(4)
C(85)	59(4)	70(4)	117(6)	-7(4)	36(4)	-2(3)
C(86)	65(4)	70(4)	83(4)	-7(3)	19(3)	-7(3)
C(87)	86(5)	56(3)	99(5)	-7(3)	41(4)	2(3)
C(88)	77(4)	71(4)	102(6)	-24(4)	25(4)	-8(3)
C(89)	74(4)	87(5)	82(5)	-10(4)	22(4)	16(4)
C(90)	82(4)	64(4)	101(5)	1(4)	58(4)	9(3)
C(91)	61(3)	61(3)	88(5)	-16(3)	39(3)	-1(3)
C(92)	71(4)	61(4)	77(4)	-16(3)	23(3)	13(3)
C(93)	57(3)	85(4)	57(3)	13(3)	17(3)	-1(3)
C(94)	52(3)	90(5)	66(4)	-6(3)	21(3)	-4(3)
C(95)	53(3)	82(5)	100(5)	-10(4)	2(4)	2(3)
C(96)	58(4)	109(6)	95(5)	36(5)	3(4)	-36(4)
C(97)	58(4)	134(7)	63(4)	7(4)	19(3)	-13(4)
C(98)	56(3)	102(5)	67(4)	-3(4)	23(3)	4(3)
Cu(1)	33(1)	36(1)	39(1)	4(1)	17(1)	4(1)
Cu(2)	38(1)	51(1)	54(1)	-2(1)	25(1)	6(1)
Cu(3)	32(1)	44(1)	73(1)	-6(1)	24(1)	4(1)
C(41)	29(2)	35(2)	68(3)	13(2)	10(2)	-2(2)
S(2)	28(1)	41(1)	29(1)	-1(1)	9(1)	0(1)
O(2)	34(2)	57(3)	34(2)	-13(2)	12(2)	-7(2)
C(55)	29(2)	35(2)	36(3)	-3(2)	8(2)	-5(2)
C(56)	31(2)	32(2)	34(3)	-1(2)	17(3)	-3(2)
C(57)	35(3)	33(3)	33(3)	3(2)	16(3)	-1(2)
C(58)	40(3)	35(3)	39(3)	0(3)	13(3)	1(3)
C(59)	50(3)	38(3)	40(3)	7(2)	14(3)	9(3)
C(60)	48(4)	31(3)	49(3)	4(2)	22(3)	7(3)
C(61)	40(3)	33(2)	44(3)	1(2)	21(3)	-3(2)
C(62)	48(4)	36(3)	55(3)	-5(2)	28(3)	-13(2)
C(63)	50(3)	44(3)	52(3)	-13(2)	27(3)	-18(3)
C(64)	68(4)	48(3)	61(3)	-23(3)	28(3)	-23(3)

C(65)	74(4)	59(4)	61(3)	-30(3)	20(3)	-28(4)
C(66)	62(3)	65(4)	51(3)	-25(3)	5(3)	-23(3)
C(67)	45(3)	53(3)	44(3)	-20(3)	5(3)	-16(3)
C(68)	35(2)	42(2)	44(3)	-14(2)	12(3)	-13(2)
S(2')	25(2)	33(2)	43(3)	7(2)	8(2)	6(2)
O(2')	22(5)	47(5)	53(6)	13(5)	15(4)	11(4)
C(55')	27(4)	31(3)	35(4)	-4(3)	14(4)	-6(3)
C(56')	31(4)	34(3)	34(4)	5(3)	19(4)	-2(3)
C(57')	36(4)	31(4)	35(5)	6(4)	16(4)	4(4)
C(58')	41(4)	35(5)	36(5)	9(4)	12(4)	6(5)
C(59')	48(5)	32(5)	45(5)	8(4)	13(4)	9(5)
C(60')	46(5)	33(4)	44(4)	7(4)	19(4)	7(5)
C(61')	41(4)	31(3)	44(4)	2(3)	22(4)	-2(4)
C(62')	46(5)	38(4)	52(4)	-5(4)	26(4)	-10(4)
C(63')	49(4)	42(4)	53(4)	-16(4)	23(4)	-18(4)
C(64')	64(5)	47(4)	58(5)	-22(4)	25(4)	-19(5)
C(65')	68(5)	53(5)	58(5)	-31(5)	17(5)	-20(5)
C(66')	62(5)	53(5)	52(5)	-28(4)	9(5)	-16(5)
C(67')	48(4)	49(5)	47(5)	-21(4)	6(4)	-15(4)
C(68')	39(4)	42(4)	45(4)	-12(3)	12(4)	-11(4)
Cu(4)	32(1)	41(1)	59(1)	3(1)	21(1)	7(1)
F(1)	58(2)	46(2)	60(2)	-16(1)	24(1)	-16(1)
F(2)	58(2)	79(2)	39(2)	-8(1)	6(1)	3(2)
F(3)	94(2)	64(2)	59(2)	26(2)	30(2)	10(2)
F(4)	79(2)	46(2)	79(2)	3(2)	30(2)	-23(2)
F(5)	48(2)	61(2)	50(2)	-6(1)	7(1)	-13(1)
F(6)	85(3)	80(2)	127(3)	-33(2)	49(2)	-21(2)
F(7)	95(3)	121(3)	103(3)	-43(3)	25(2)	18(3)
F(8)	107(3)	158(4)	81(3)	8(3)	57(2)	40(3)
F(9)	85(3)	104(3)	121(3)	39(3)	61(2)	12(2)
F(10)	81(2)	70(2)	97(3)	-9(2)	40(2)	-13(2)
F(11)	54(2)	47(2)	65(2)	-19(1)	22(1)	-14(1)
F(12)	80(2)	88(2)	44(2)	-4(2)	21(2)	-3(2)
F(13)	142(4)	70(2)	101(3)	34(2)	43(3)	-4(2)
F(14)	101(3)	45(2)	172(4)	4(2)	23(3)	-30(2)
F(15)	57(2)	63(2)	127(3)	-9(2)	-26(2)	-16(2)
F(16)	59(2)	57(2)	74(2)	-10(2)	15(2)	-3(1)
F(17)	88(3)	116(3)	58(2)	-30(2)	19(2)	25(2)
F(18)	119(3)	143(4)	71(2)	27(2)	69(2)	34(3)
F(19)	71(2)	91(2)	103(3)	33(2)	56(2)	-2(2)
F(20)	55(2)	57(2)	65(2)	2(1)	24(1)	-12(1)
O(1)	34(2)	43(2)	42(2)	9(1)	12(1)	8(1)
S(1)	33(1)	38(1)	40(1)	1(1)	12(1)	2(1)

S6 NMR Spectra

$[Cu(C_6F_5)]_4(Tol_2SO)_2$ 7:

^{1}H NMR ($CDCl_3$, 500 MHz)

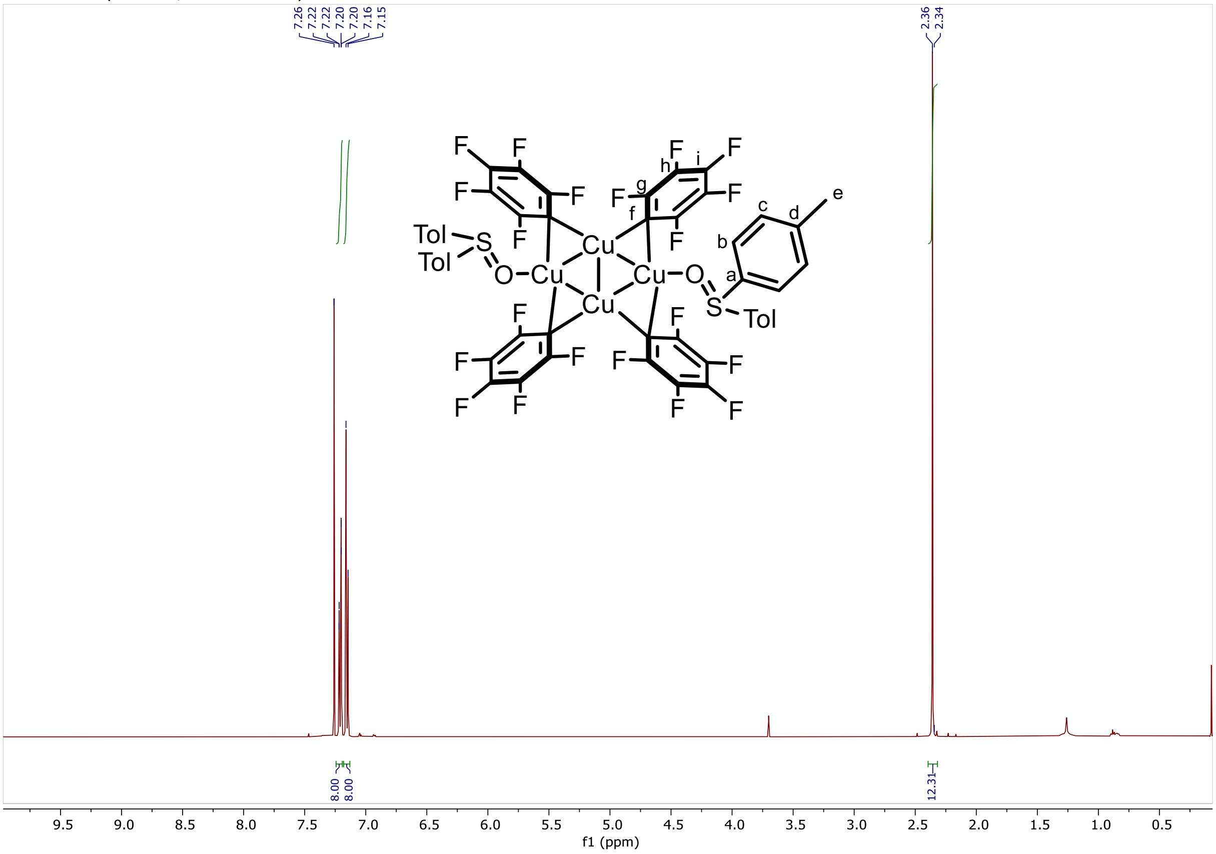

^{13}C{^{1}H} NMR ($CDCl_3$, 126 MHz)

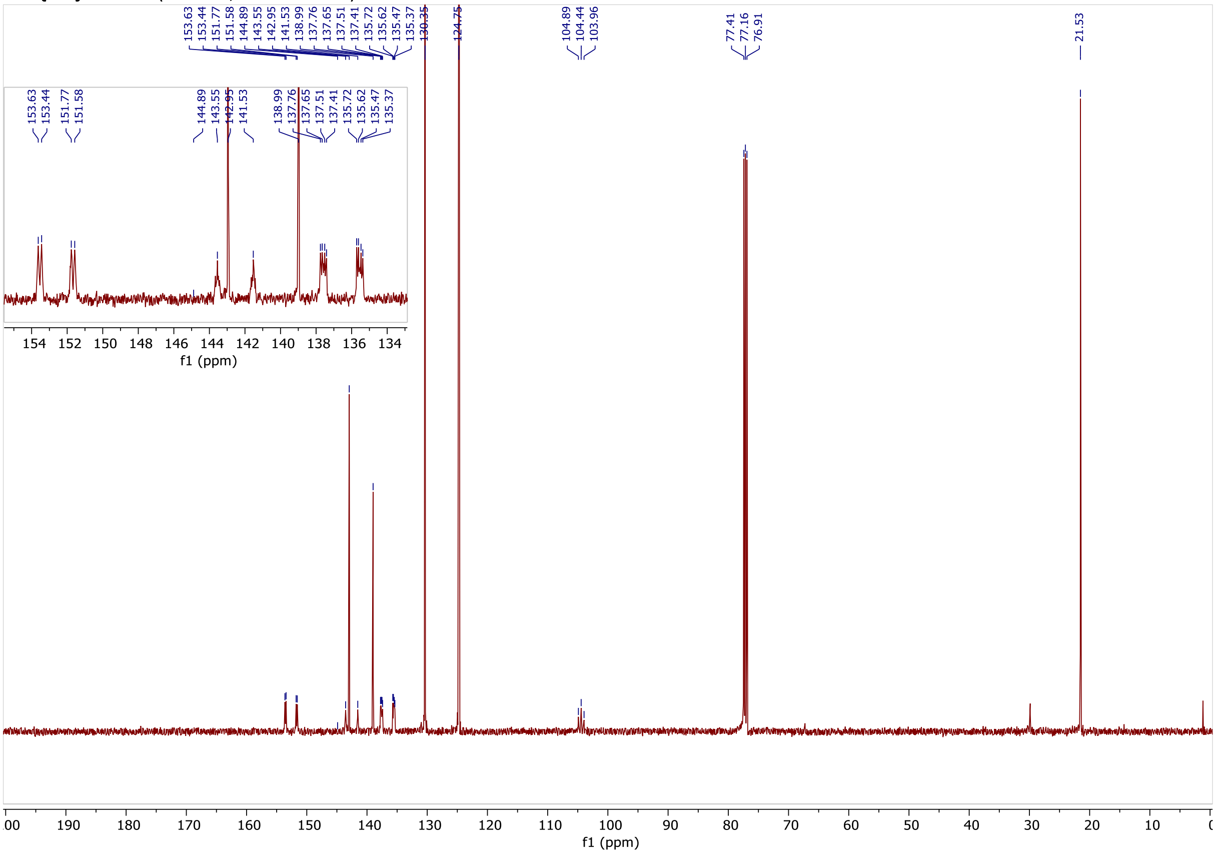

HSQC $^{19}F/^{13}C\{^{1}H\}$ ($CDCl_3$)

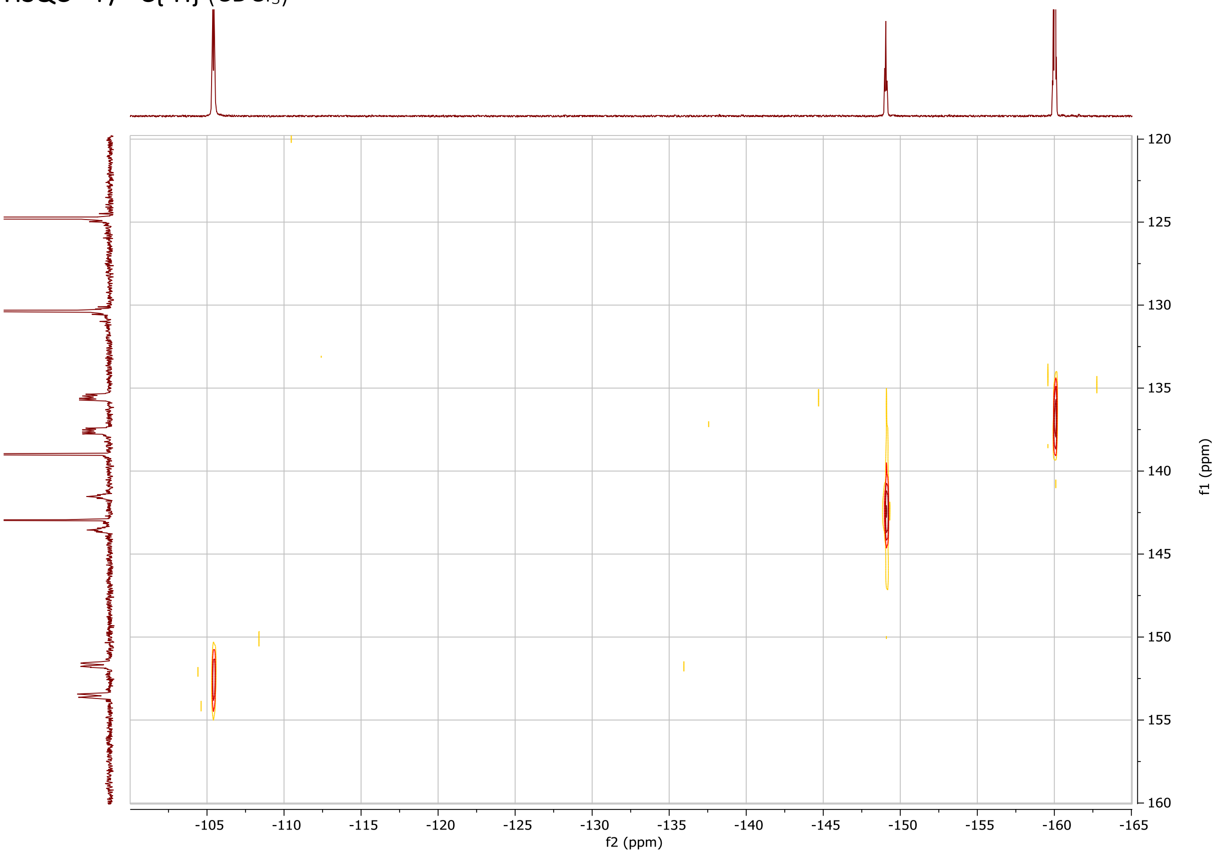

^{19}F NMR ($CDCl_3$, 282 MHz)

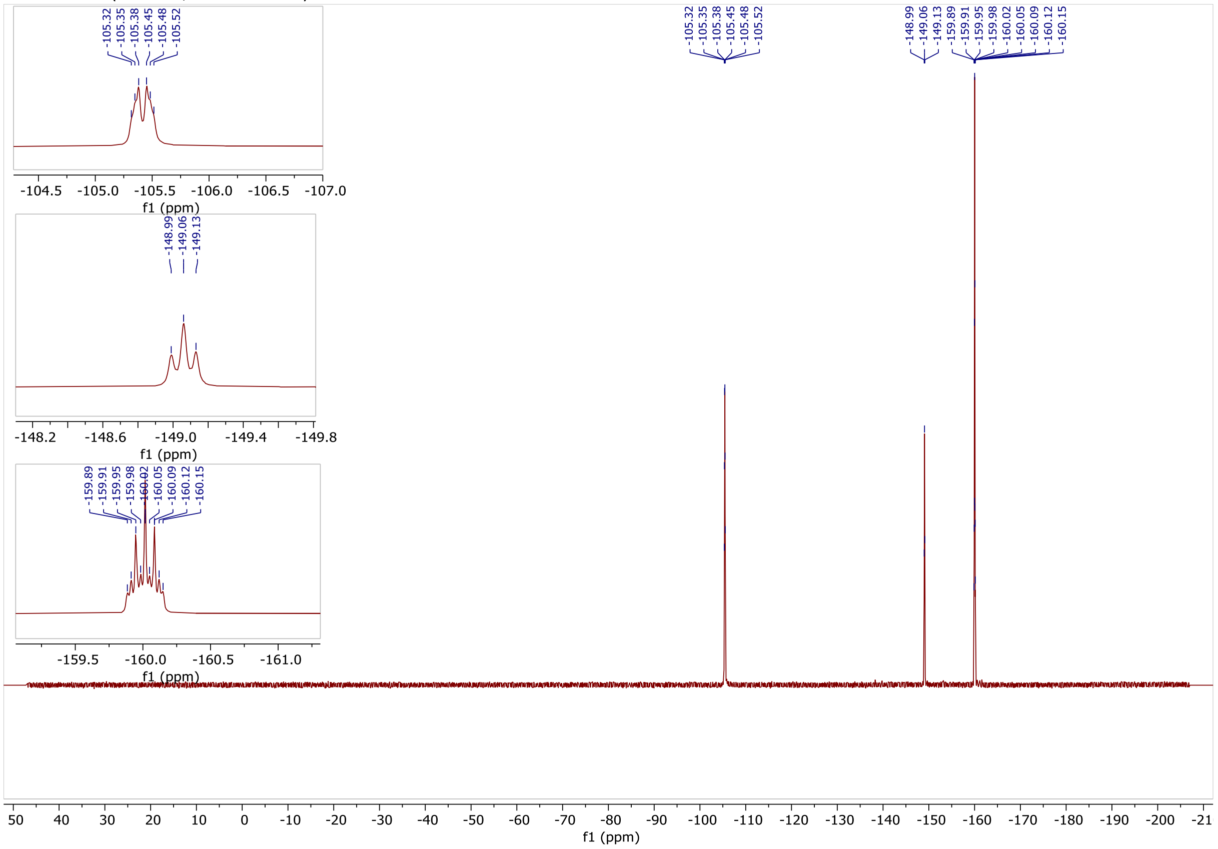

[Cu(C_6F_5)]$_4$(DBTO)$_2$ 6:

^{1}H NMR ($CDCl_3$, 600 MHz)

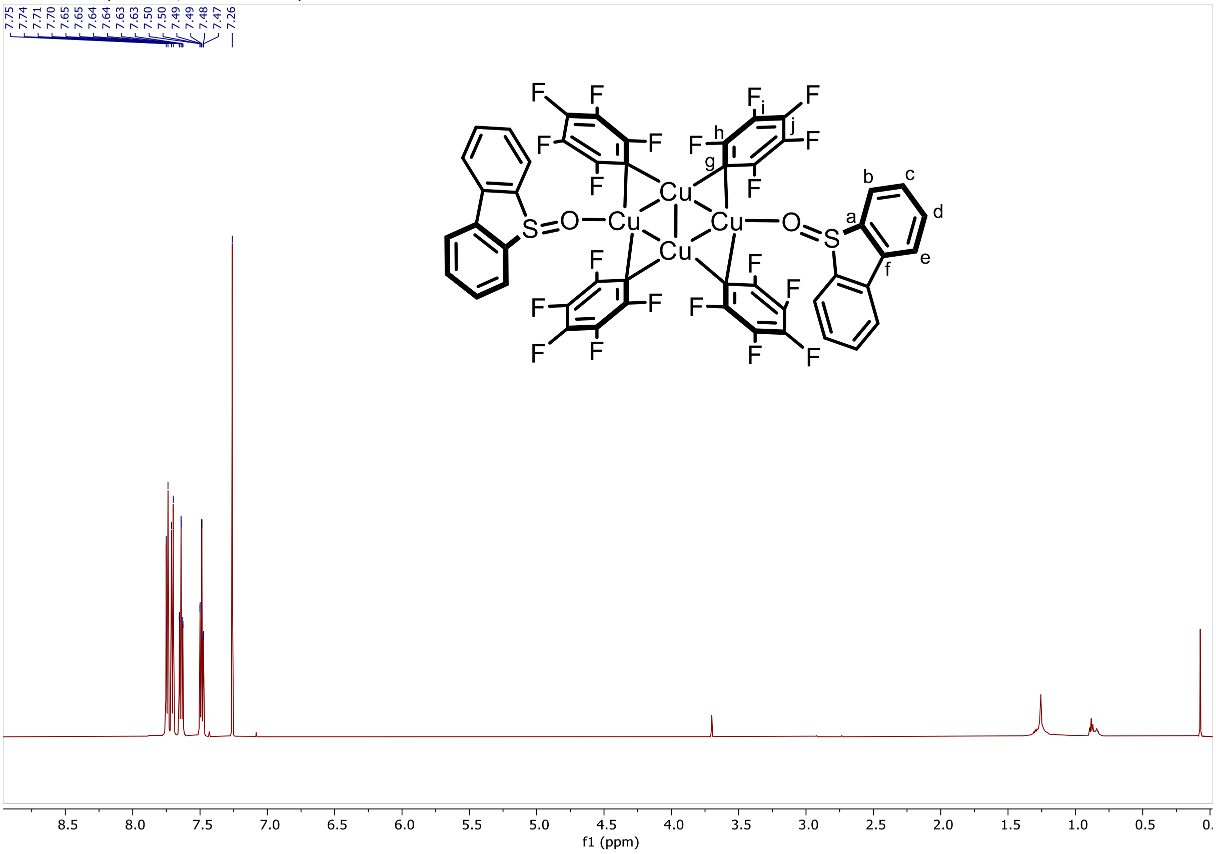

^{13}C{^{1}H} NMR ($CDCl_3$, 151 MHz)

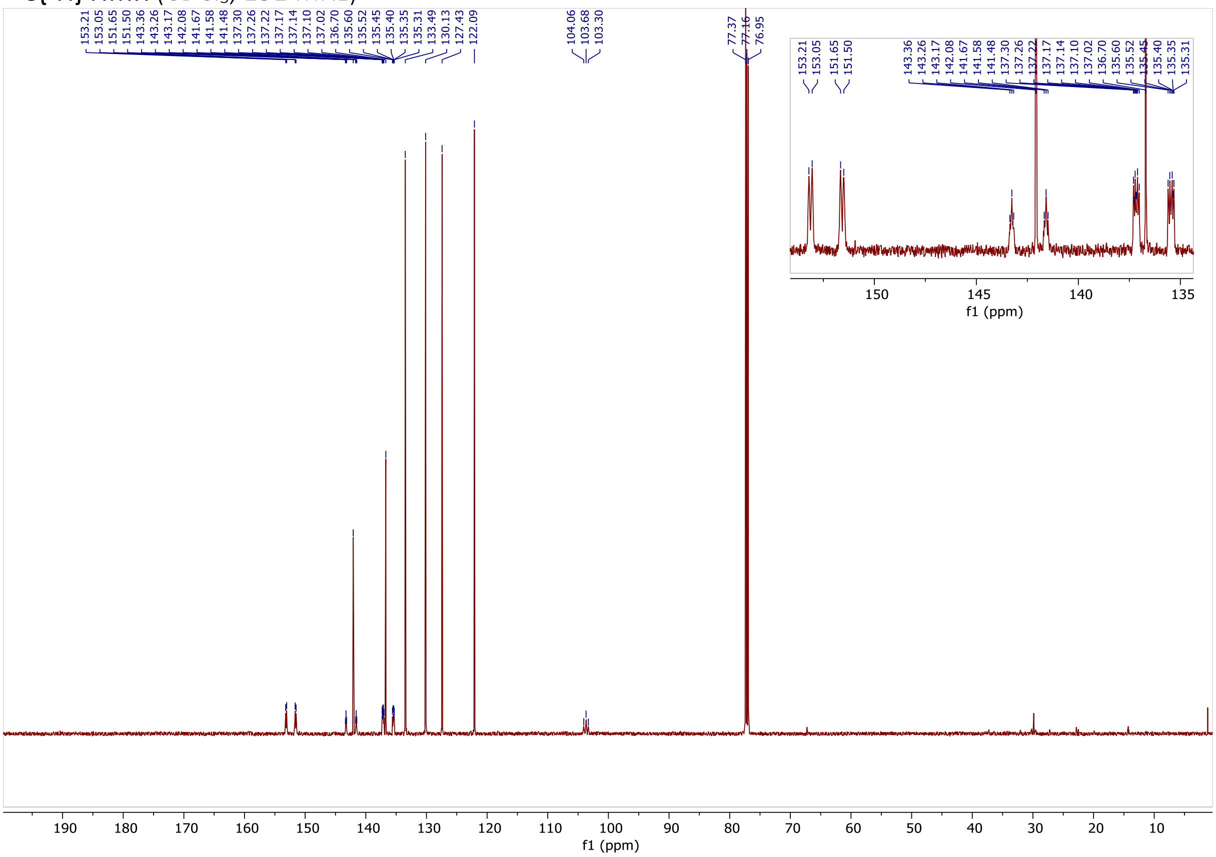

^{19}F NMR ($CDCl_3$, 282 MHz)

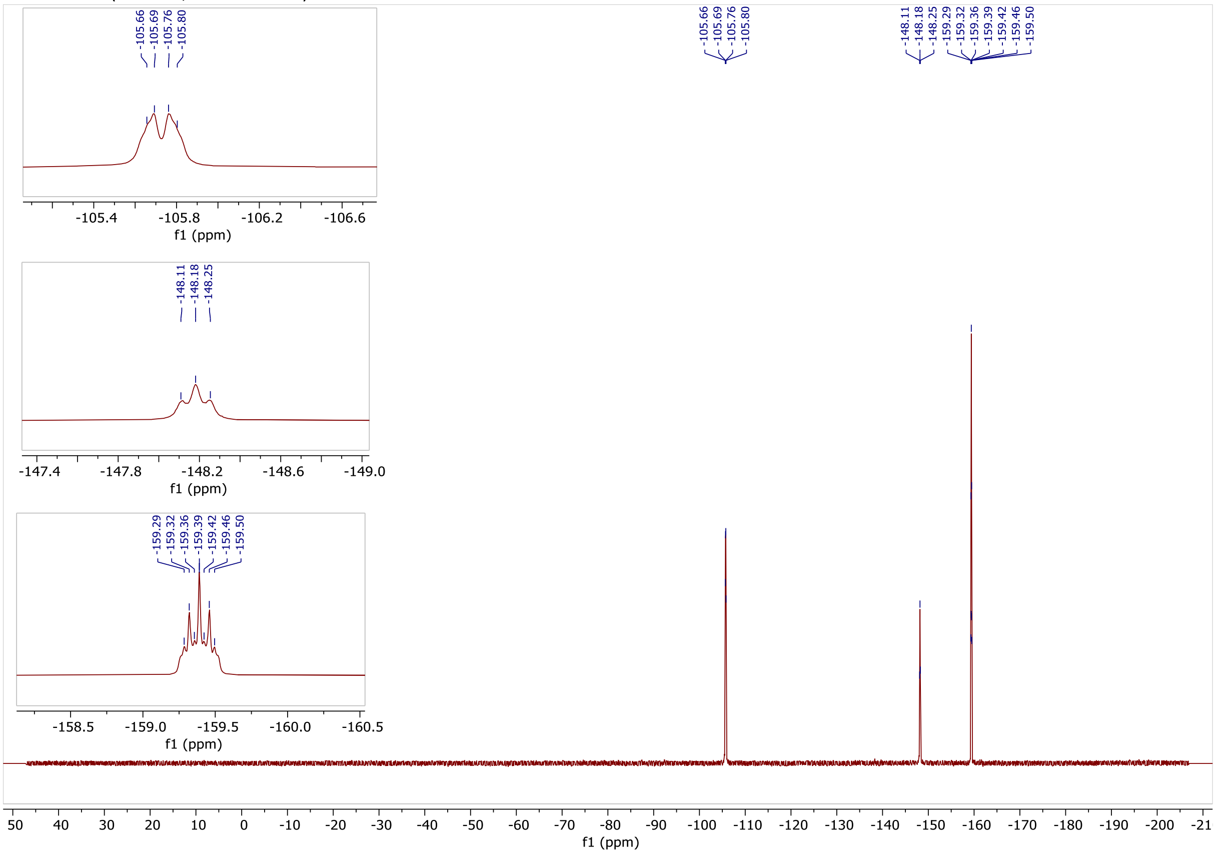

$[Cu(C_6F_5)]_4(Anthra_2SO)_2$ 8:

^1H NMR (CD_2Cl_2, 600 MHz)

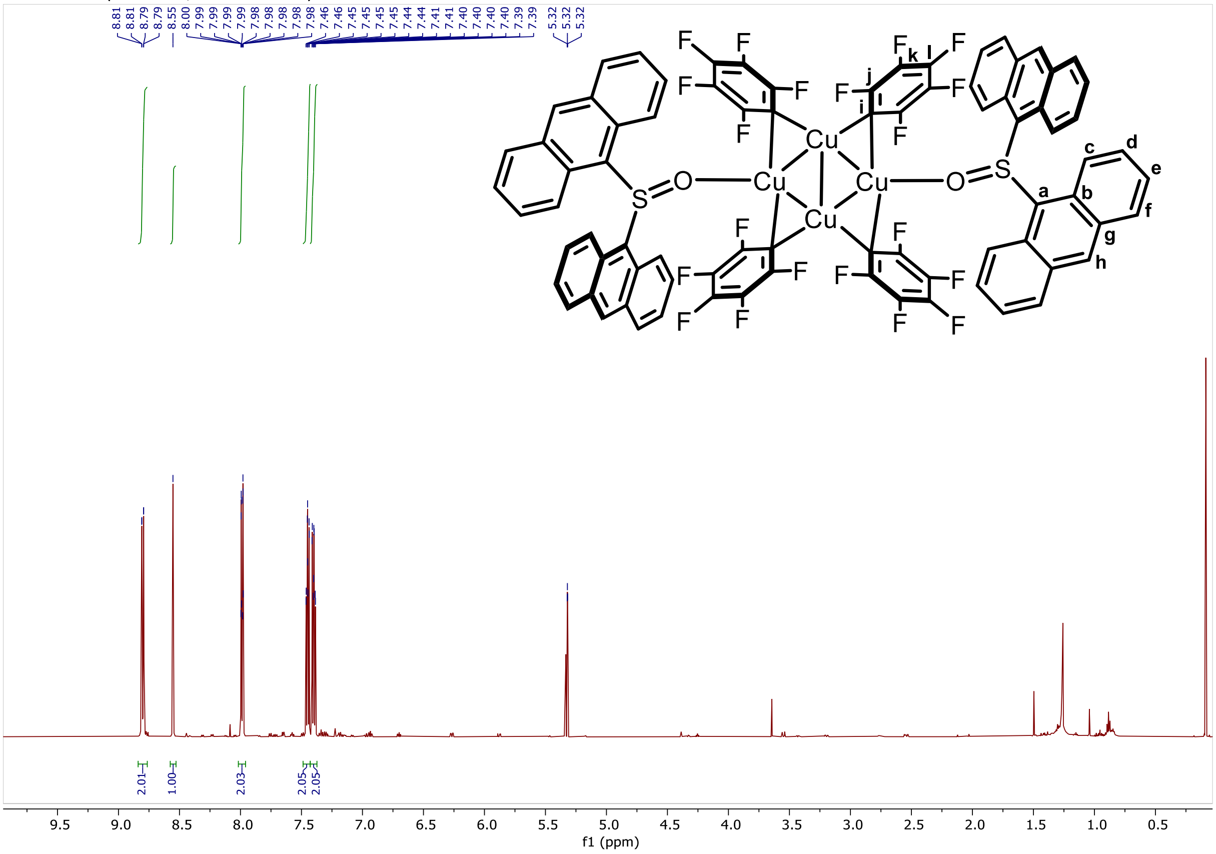

$^{13}C\{^1H\}$ NMR (CD_2Cl_2, 151 MHz)

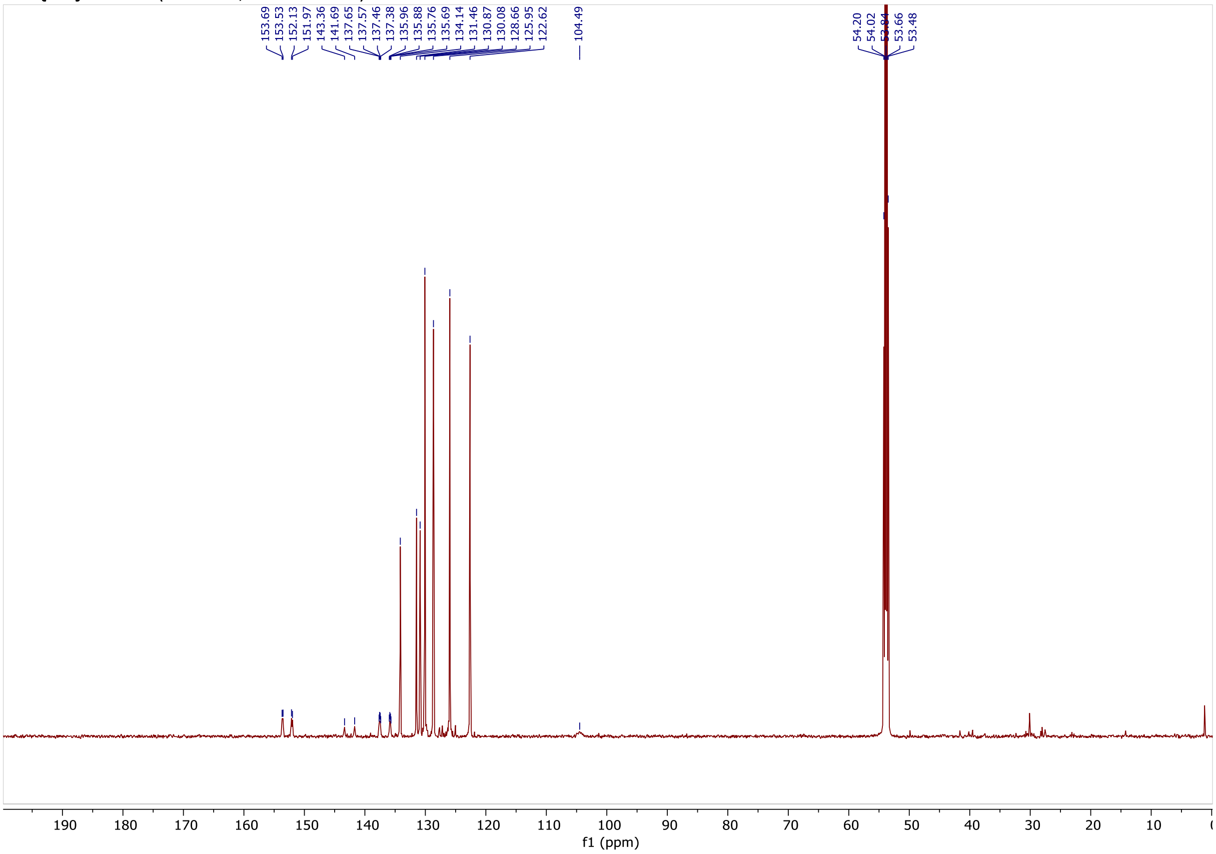

^{19}F NMR (CD_2Cl_2, 282 MHz)

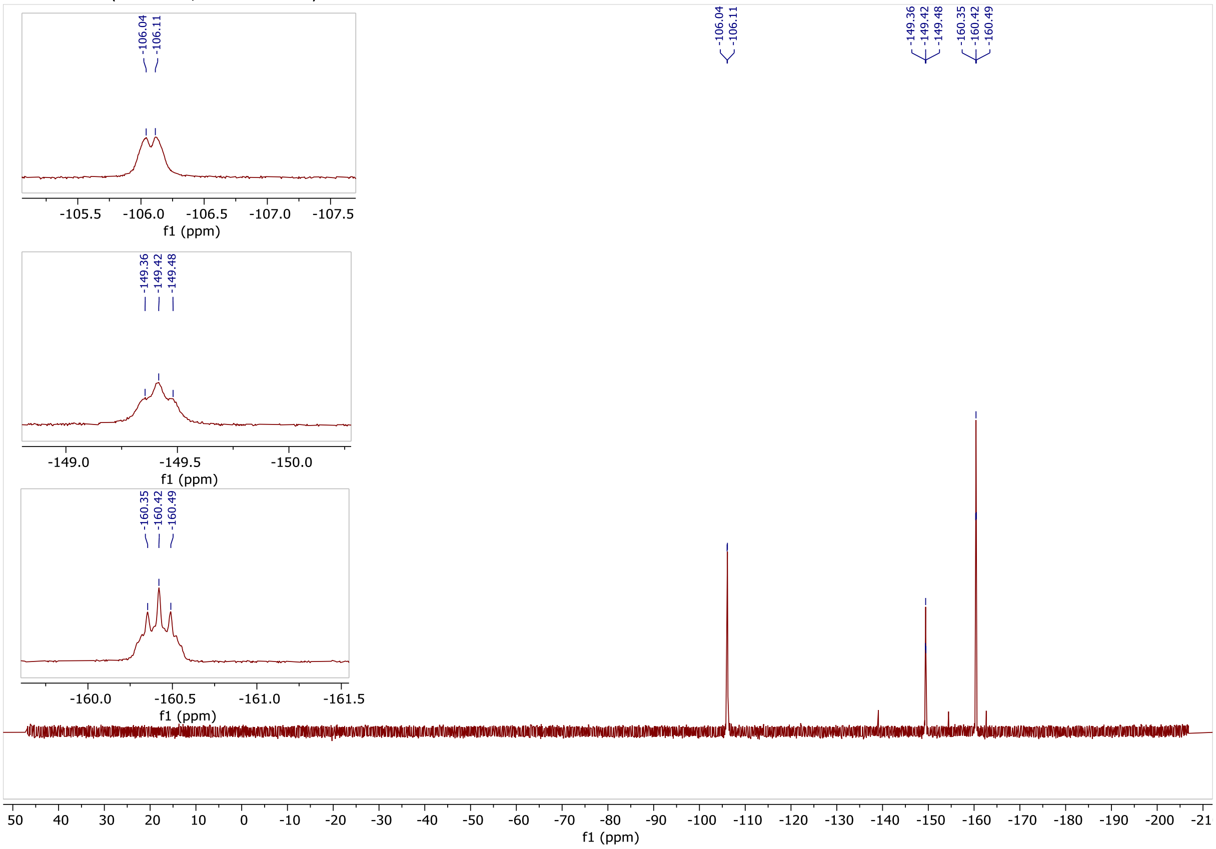

9-(perfluorophenyl)anthracene 10:

^{1}H **NMR** ($CDCl_3$, 600 MHz)

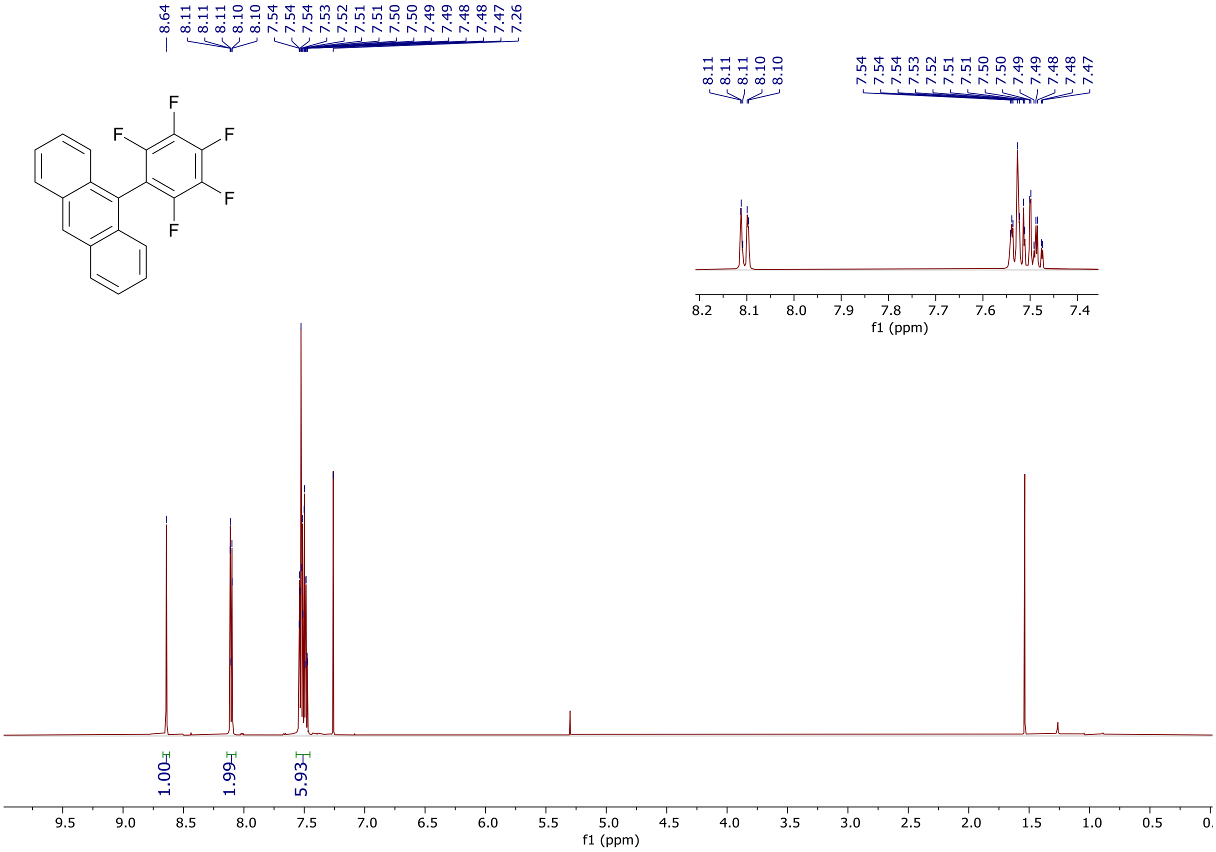

^{13}C{^{1}H} **NMR** (CD_2Cl_2, 151 MHz)

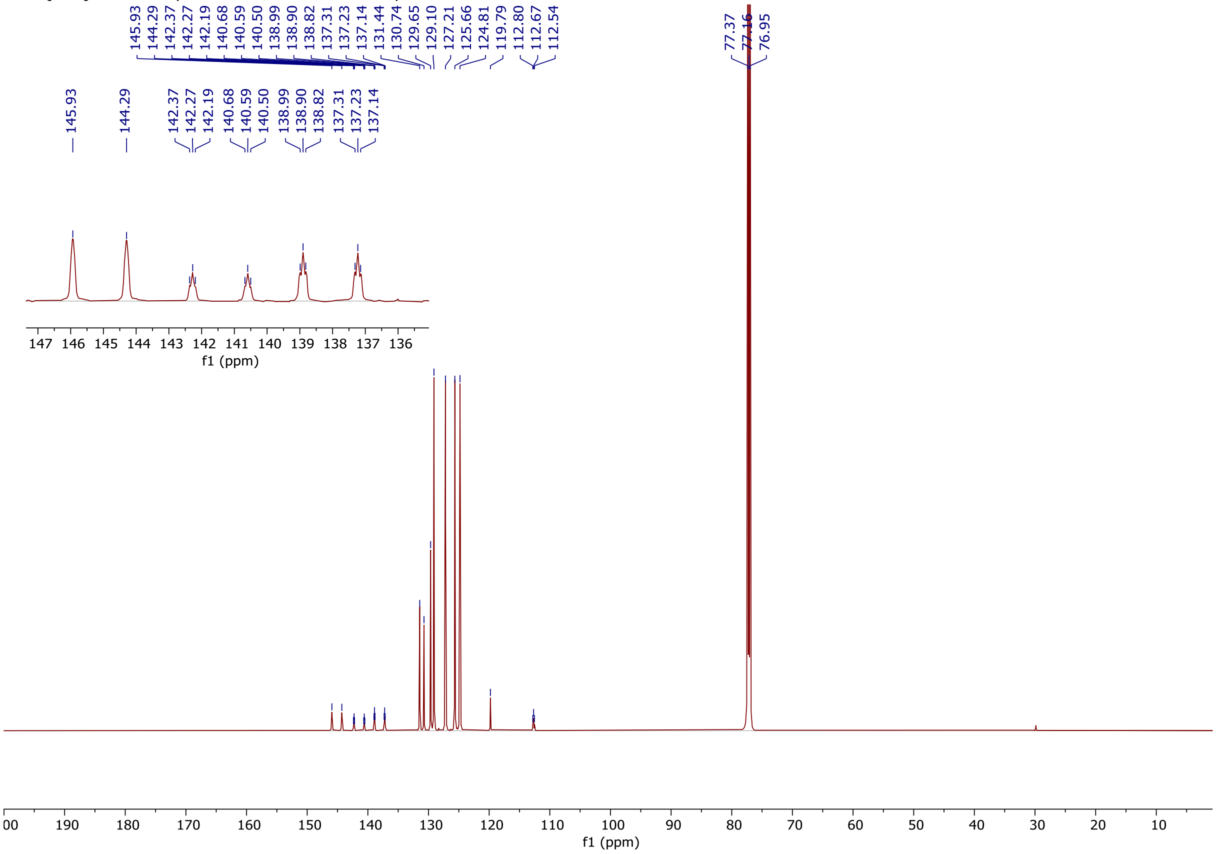

^{19}F NMR (CD_2Cl_2, 282 MHz)

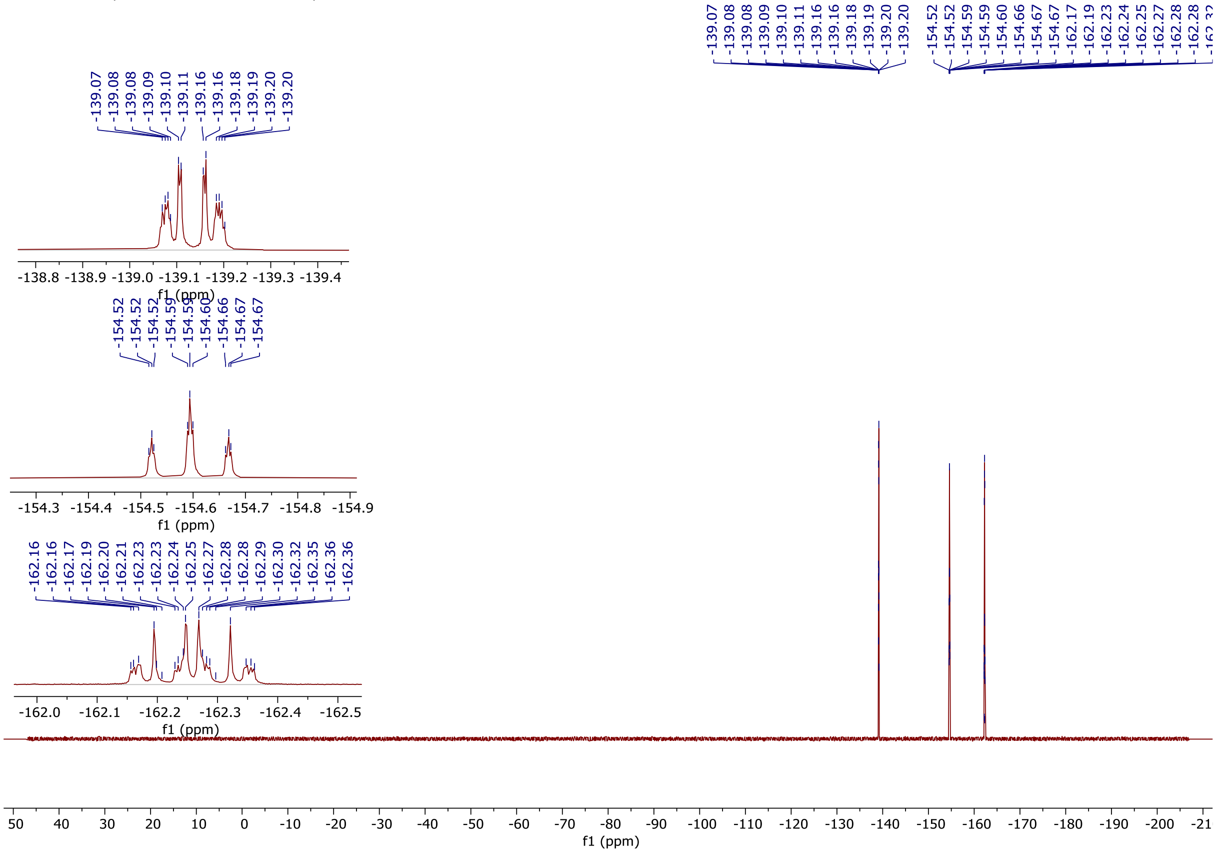

Anthracen-9-yl(perfluorophenyl)sulfane 11:

^{1}H NMR ($CDCl_3$, 600 MHz)

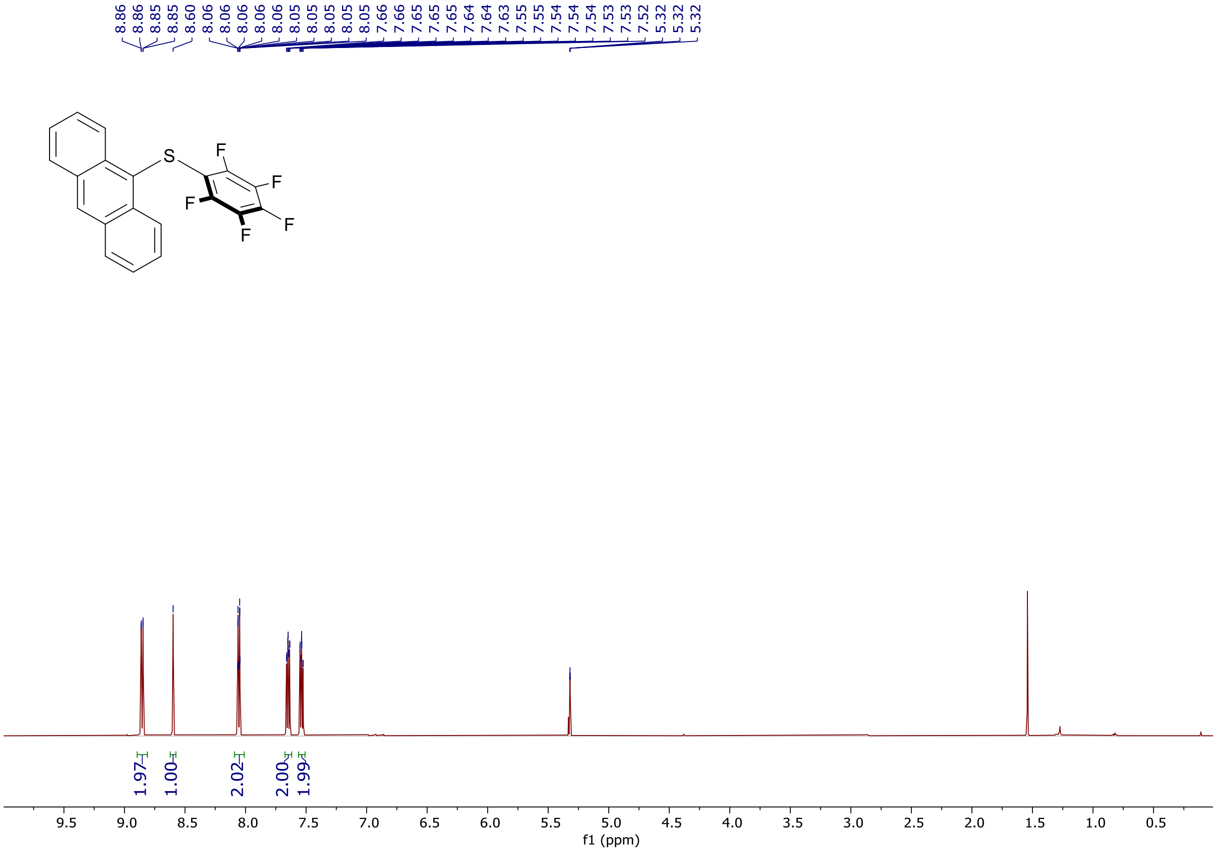

^{13}C{^{1}H} NMR (CD_2Cl_2, 151 MHz)

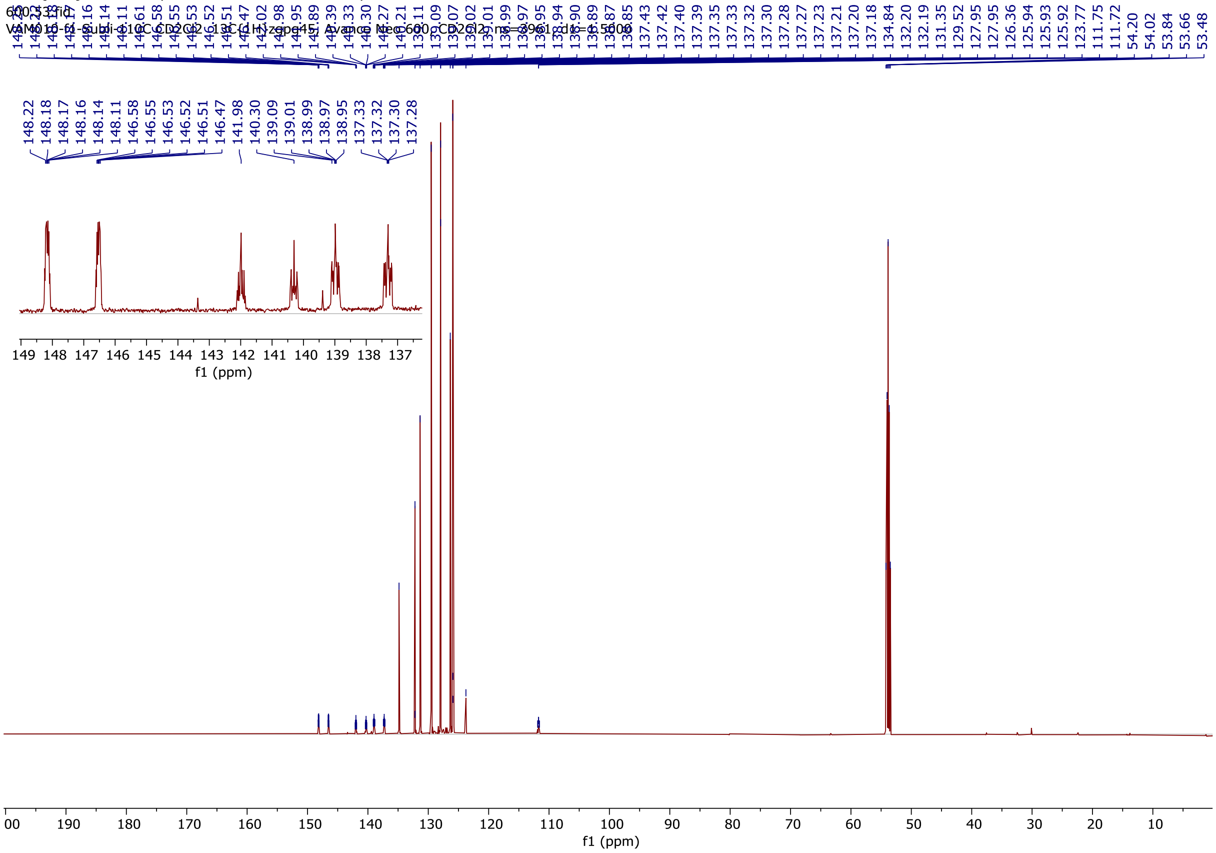

^{19}F NMR (CD_2Cl_2, 282 MHz)

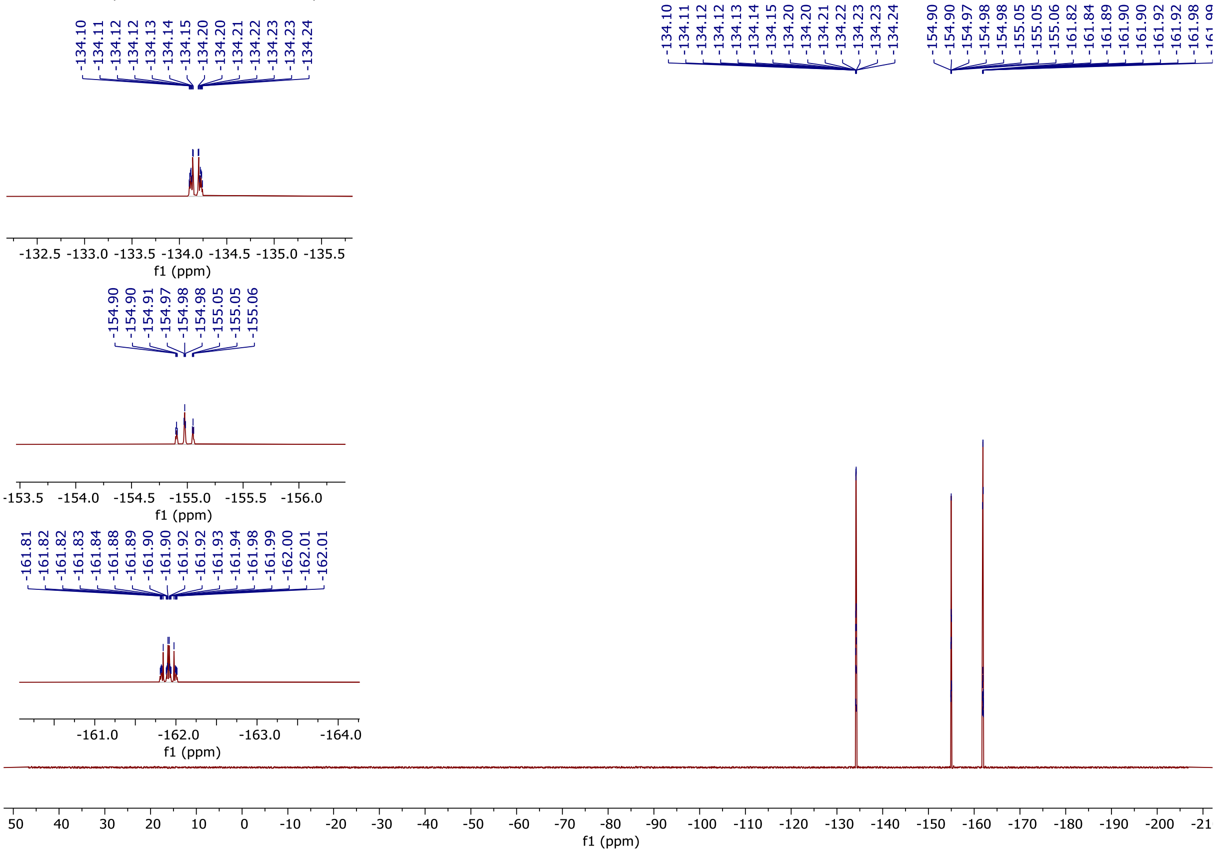

perfluoro-1,1'-biphenyl 9:

^{19}**F NMR** (CD_2Cl_2, 282 MHz)

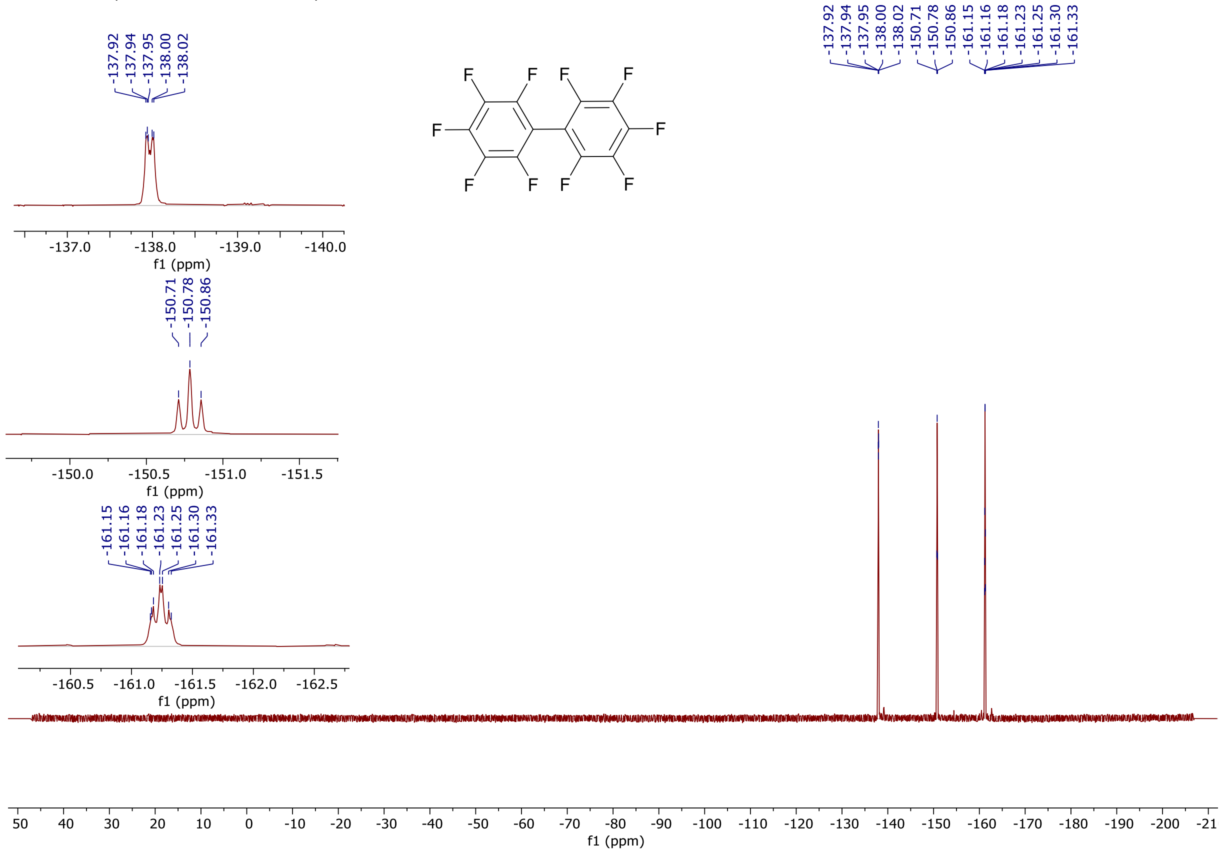

Bis(perfluorophenyl)sulfane 12:

^{19}F NMR (CD_2Cl_2, 282 MHz)

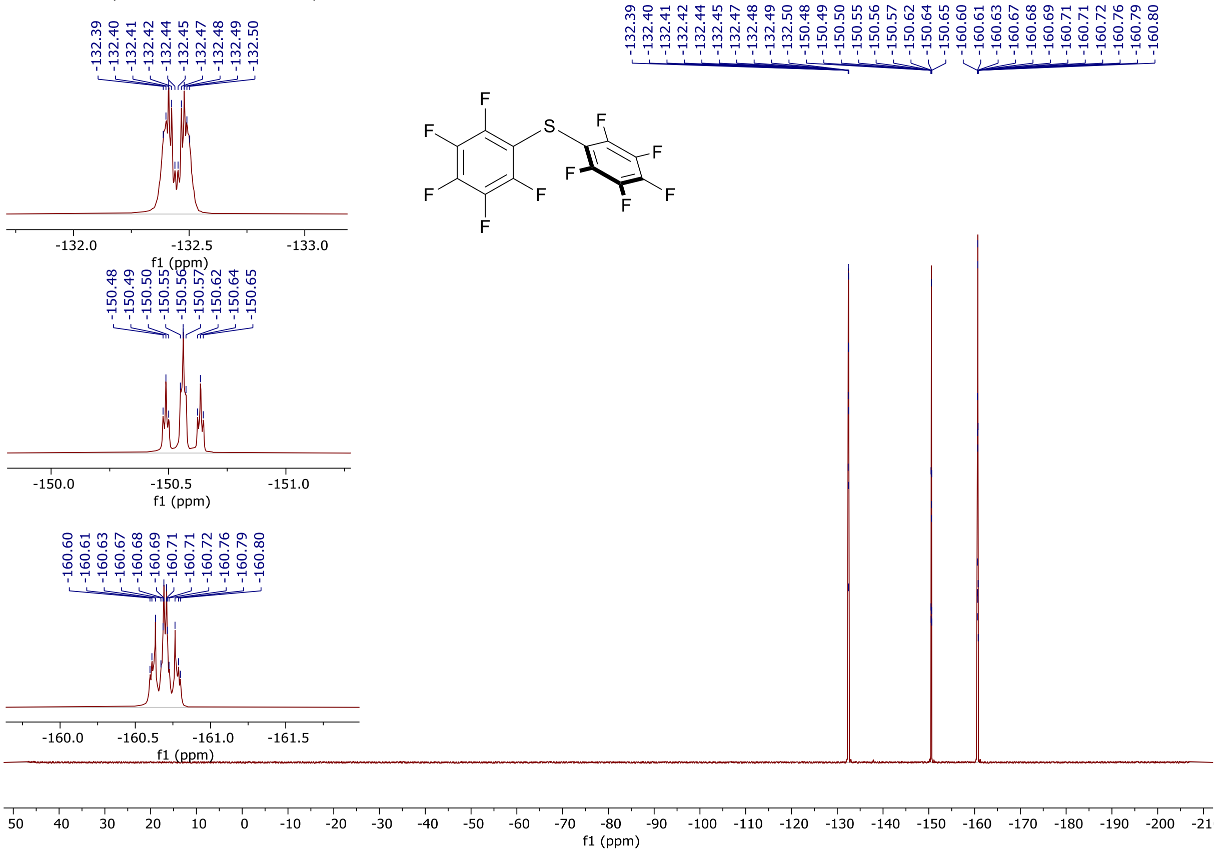

S7 IR Spectra

$[Cu(C_6F_5)]_4(DBTO)_2$ 6:

IR (neat)

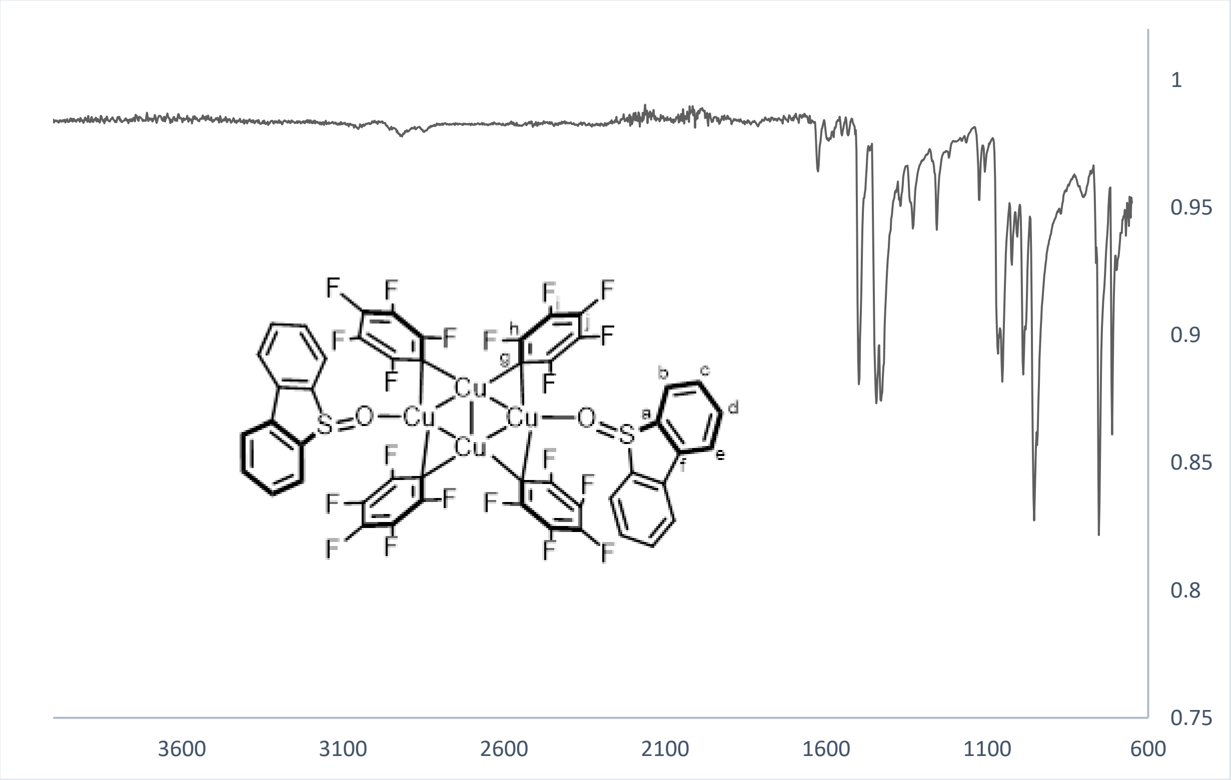

$[Cu(C_6F_5)]_4(Tol_2SO)_2$ 7:

IR (neat)

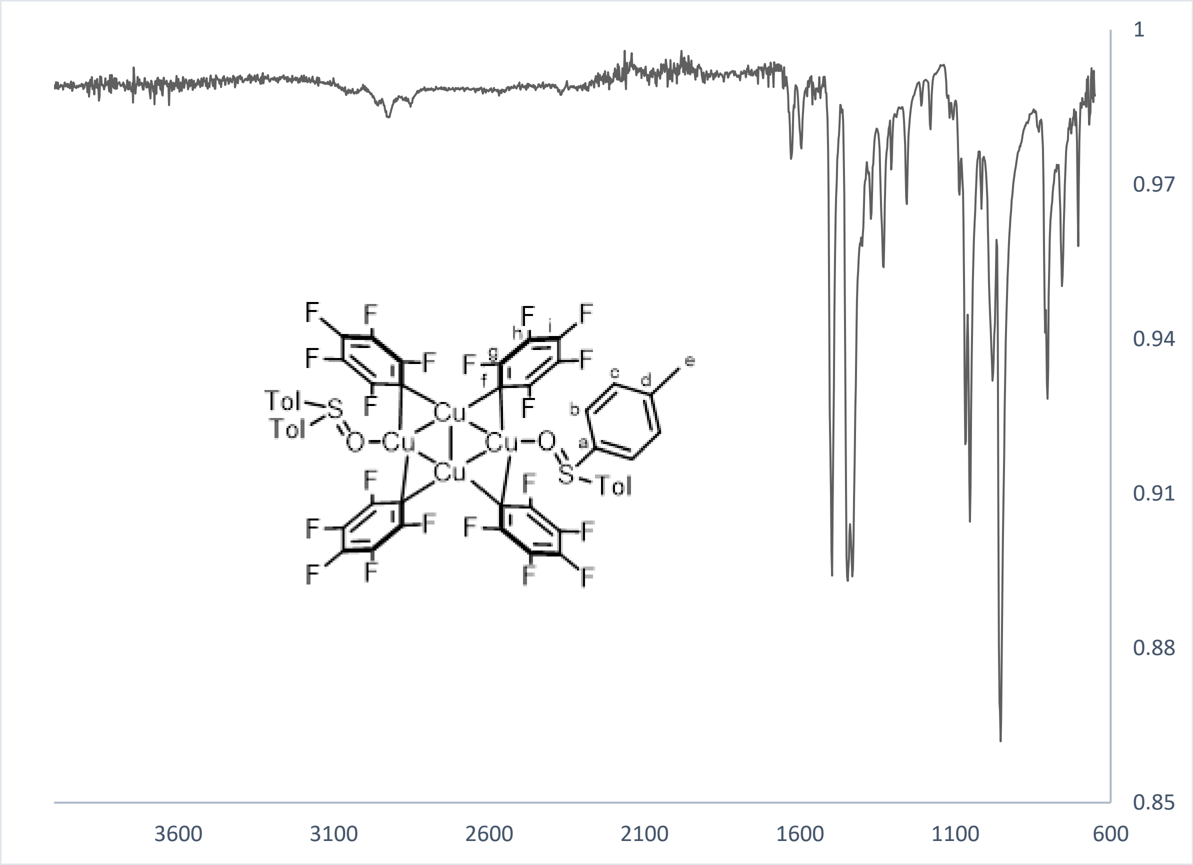

[Cu(C_6F_5)]$_4$(Anthra$_2$SO)$_2$ 8:

IR (neat)

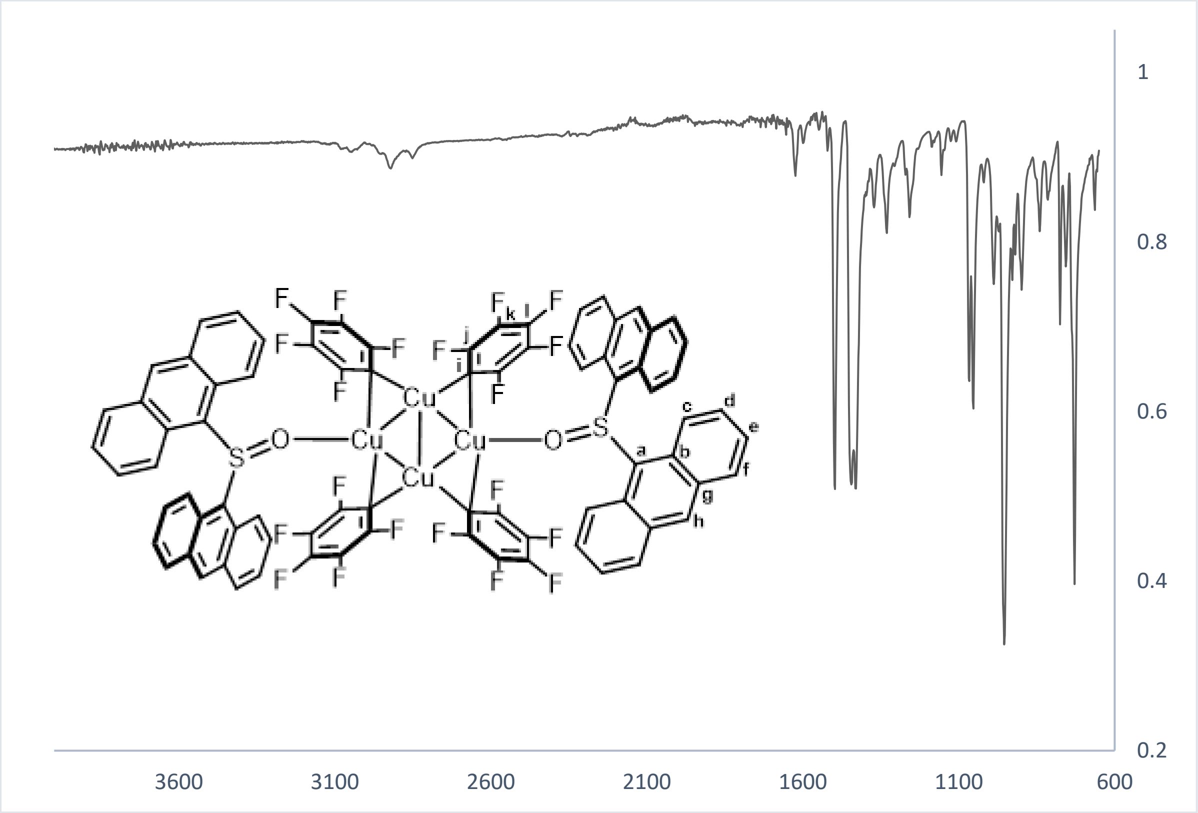

Supplement: Supplementary file 1 [file molecules-29-03332-s001.zip › molecules-3088538-supplementary.pdf]
